# How International Health System Austerity Responses to the 2008 Financial Crisis Impacted Health System and Workforce Resilience – A Realist Review

**DOI:** 10.34172/ijhpm.2022.7420

**Published:** 2022-12-24

**Authors:** Padraic Fleming, Louise Caffrey, Sara Van Belle, Sarah Barry, Sara Burke, Jacki Conway, Rikke Siersbaek, David Mockler, Steve Thomas

**Affiliations:** ^1^Centre for Health Policy and Management, Trinity College Dublin, The University of Dublin, Dublin 2, Ireland.; ^2^School of Social Work and Social Policy, Trinity College Dublin, The University of Dublin, College Green, Dublin 2, Ireland.; ^3^FWO, Institute of Tropical Medicine, Antwerp, Belgium.; ^4^Everlake, 5 Marine Terrace, Dun Laoghaire, Dublin, Ireland.; ^5^Library Reader Services, Trinity College Dublin, The University of Dublin, St James Hospital, Dublin 8, Ireland.

**Keywords:** Austerity, Health System Resilience, Preparedness, Governance, Communication, Value-Driven Decision-Making

## Abstract

**Background:** The Great Recession, following the 2008 financial crisis, led many governments to adopt programmes of austerity. This had a lasting impact on health system functionality, resources, staff (numbers, motivation and morale) and patient outcomes. This study aimed to understand how health system resilience was impacted and how this affects readiness for subsequent shocks.

**Methods:** A realist review identified legacies associated with austerity (proximal outcomes) and how these impact the distal outcome of health system resilience. EMBASE, CINAHL, MEDLINE, EconLit and Web of Science were searched (2007–May 2021), resulting in 1081 articles. Further theory-driven searches resulted in an additional 60 studies. Descriptive, inductive, deductive and retroductive realist analysis (utilising excel and Nvivo) aided the development of context-mechanism-outcome configurations (CMOCs), alongside stakeholder engagement to confirm or refute emerging results. Causal pathways, and the interplay between context and mechanisms that led to proximal and distal outcomes, were revealed. The refined CMOCs and policy recommendations focused primarily on workforce resilience.

**Results:** Five CMOCs demonstrated how austerity-driven policy decisions can impact health systems when driven by the priorities of external agents. This created a real or perceived shift away from the values and interests of health professionals, a distrust in decision-making processes and resistance to change. Their values were at odds with the realities of implementing such policy decisions within sustained restrictive working conditions (rationing of staff, consumables, treatment options). A diminished view of the profession and an inability to provide high-quality, equitable, and needs-led care, alongside stagnant or degraded working conditions, led to moral distress. This can forge legacies that may adversely impact resilience when faced with future shocks.

**Conclusion:** This review reveals the importance of transparent, open communication, in addition to co-produced policies in order to avoid scenarios that can be detrimental to workforce and health system resilience.

## Background

 In just over a decade, health systems internationally have dealt with two major global shocks, namely the Great Recession following the 2008 financial crisis and the coronavirus disease 2019 (COVID-19) pandemic. This research was conducted as part of a wider programme of research (RESTORE – Resilience to Reform) exploring the legacy of the austerity period and how this changed health systems for good or ill, while examining the causality of how shocks to the system challenge or even facilitate reform.^1^ Understanding the legacy of shocks is important for predicting and being prepared for future shocks, maximising absorptive capacity alongside the management of system stresses, with the potential to transform and evolve – ideally into something better.^2^ This is particularly relevant during times of global instability due to ongoing shocks related to COVID-19 and the war in Ukraine, with possible consequential economic shocks that could shrink resources for the healthcare system.^3^

 There has been much recent interest in, and literature on, the concept of health system resilience though not always consensus.^4-6^ Most definitions focus on the health system response to a shock and how the system can absorb, adapt and transform to cope with sudden changes. In essence, the focus is on how the system can bounce back from a shock without undermining performance and even, potentially, improving resilience. Nevertheless, very little is written or understood about the legacy of shocks on health system performance after the initial shock has receded, limiting our ability to learn from and be better prepared for future shocks. Indeed a better understanding of legacies may lead to consensus around the concept of health system resilience. Given this need for a deeper understanding, a theory-driven realist review was chosen to synthesise existing literature, analysing the interplay between context and underlying mechanisms to understand how outcomes of interest are achieved.^7^

 For the purposes of this review, health system resilience is defined as the ability to prepare for, manage (absorb, adapt and transform) and learn from shocks.^2^ It is further conceptualised using the 4-stage shock lifecycle framework, acknowledging that health system resilience is multifaceted including the ability of the health system to (1) prepare for; (2) respond to initial onset; (3) manage (absorb, adapt and transform); and (4) learn from shocks to improve health system performance,^2^ with particular emphasis on the final stage, and how legacies impact health system resilience. This addresses a gap in existing literature reviews. For example, Barasa et al^8^ emphasised the importance of workforce supply and motivation, information, leadership, coordination and adequate material resources for health system resilience but did not address the focus on the legacy of, and learning from, shocks. This may be because the authors were keen to emphasise that resilience concerns everyday stresses facing health systems, particularly in low- and middle-income countries. Nevertheless, shock legacy may impact on future effective absorption of new shocks, alongside the management of stresses. Another review of 71 empirical studies on health system resilience from 2008 to 2019, found that most studies only addressed aspects of resilience related to absorptive and adaptive capacities, with legitimacy of institutions and transformative resilience seldom addressed.^5^ Furthermore the review highlighted that the interpretation of resilience, within the peer-reviewed health literature, often lacks theoretical underpinnings, a further justification for this realist review.

###  Aim and Objectives

 The aim of this realist review was to understand health system legacies of the Great Recession following the 2008 financial crisis; the underlying mechanisms and their theoretical origins; and how these influenced and impacted health system resilience, and its ability to respond to future shocks, with five objectives:

1. Identify health system legacies associated with the Great Recession;
2. Within these legacy issues, identify proximal outcomes that impact the distal outcome of health system resilience;
3. Develop evidence-informed context-mechanism-outcome configurations (CMOCs) that are closely aligned to the primary data identified;
4. Develop theoretically driven middle-range theories, that are testable through further research;
5. Present a final programme theory on the impact of legacy issues on health system resilience and how it relates to current and future shocks in order to understand and mitigate their impacts on health system resilience.

## Methods

 A realist review, which is inherently mixed-methods by design, was undertaken in line with Pawson and Tilley’s theory driven approach, aiming to understand why certain outcomes occur, for whom, in what circumstances and to what extent.^9-11^ Thus, realist reviews consider the context in which certain mechanisms are activated or triggered to achieve given outcomes. This interplay is referred to as the CMOC. According to Greenhalgh and Manzano,^12^ context is (1) an observable feature (space, place, people, things) that triggers mechanisms, and while relatively static, can set in motion a chain reaction of events or (2) context is relational and dynamic, shaping mechanisms in an emergent way depending on a specific intervention (or policy, in the case of this review). Recent literature, unpacking context and how it is used in realist research^12,13^ also points to the importance of legacies in implementation science, by acknowledging the effect of past actions or actors and how these impact subsequent actors and their activities.^14^

 Mechanisms may be defined as underlying entities, processes or structures,^15^ that are sensitive to context, often hidden and generate outcomes.^7^ For socially complex interventions, such as health systems reform, mechanisms take account of resources or opportunities and how people reason and respond with these resources, which lead to certain outcomes. These relational or individual-cognitive processes can drive stakeholders agency and actions – including those delivering the intervention.^13^

 There was a 6-stage approach taken to this review:

 1. Initial programme theory (IPT) development;

- Based on expertise within research team and wider stakeholder group
- Conceptually expanded, by developing ‘if, then, because’ statements, based on expert stakeholder discussion

2. Formal literature searching based on IPT;

 3. Data screening and extraction;

 4. Data analysis and preliminary CMOC development;

- Discussion with expert stakeholders to confirm, refine or refute emerging theories

5. Additional literature searches to refine CMOCs;

 6. Development of refined CMOCs and final programme theory;

- Confirmation of rationale with expert stakeholders.

The review was conducted over a 17 month period (January 2021–May 2022), in line with the study protocol published on PROSPERO^16^ and guided by the Realist And Meta-narrative Evidence Syntheses Evolving Standards (RAMESES).^7^

###  Stakeholder Engagement and Patient and Public Involvement

 Stakeholder engagement involved (1) the wider research team (n = 12) consisting primarily of national (n = 7) and international (n = 3) academics – in policy analysis, health sciences, economics, social science and implementation science, as well as government representatives (n = 2), and (2) an advisory group (n = 8) consisting of government representatives (n = 3), general practitioner representation (n = 1) and international experts (n = 2) in realist methods and health services research, as well as Public Patient Involvement (PPI) representatives (n = 2). Online stakeholder engagements were held using Zoom on three occasions. Additionally, preliminary findings were presented and discussed with the broader RESTORE advisory committee, including Ireland’s Health Service Executive and Department of Health representatives and healthcare staff, and finally at two public fora, the RESTORE annual workshop^17^ and a national Population and Health-services research conference,^18^ where feedback was captured and incorporated into ongoing analysis.

###  Stage 1. Initial Programme Theory Development

**Figure 1:**
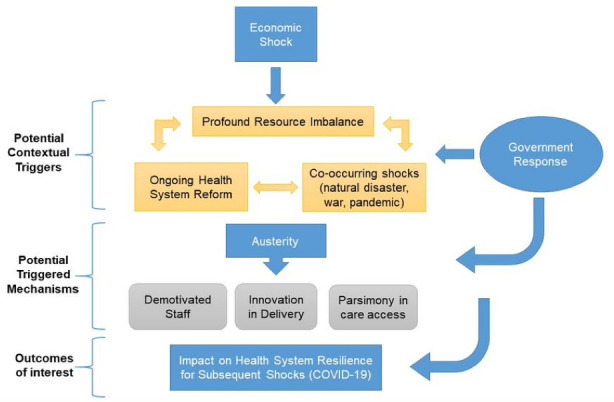
Initial Programme Theory. Abbreviation: COVID-19, coronavirus disease 2019.

 The project team, consisting of PF and ST, developed an IPT to (1) outline potential causal or descriptive relationships between key concepts within the known literature and (2) provide structure to the review findings. Guided by the RAMESES standards^7^ the IPT set out: the key components (functions, strategies or activities) of the programme; the expected or potential outcomes; and the components that potentially contribute to particular outcomes (Figure 1).

 The IPT drew on expertise within the research team, wider stakeholder and advisory groups (including national and international policy analysts, implementation scientist, social scientist, economist, health services researcher and PPI representatives), who were consulted in an initial stakeholder workshop (February 24, 2021). There was consensus on the IPT at a macro level, with recommendations to develop statements to further develop sub-components using realist language. The overarching contextual factors were related to the economic crisis and the years of austerity that followed, in addition to ongoing reforms or co-occurring shocks to the health system. These contextual factors triggered potential mechanisms, both positive (innovation in delivery) and negative (demotivated staff), which ultimately impact the distal outcome of health system resilience. Following an initial stakeholder consultation (February 24, 2021) the project team formulated ‘if, then, because’ statements for eight possible proximal outcomes that could potentially impact the distal outcome of health system resilience including: access; decision-making; demotivated staff (example below); innovation; public dissatisfaction; political instability; population health; short-termism; and new public management. These were constructed in realist terms, considering possible contexts, mechanisms and outcomes (see Supplementary file 1).

 “ ***If **[salaries are reduced; workload is increased; staff are lost] **then** there will be [less flexibility; lower productivity; poorer performance; less willingness to innovate; lower quality of care] **because** [staff demotivated; disengaged; burnt out].”*

###  Stage 2. Formal Literature Searching Based on IPT

**Figure 2:**
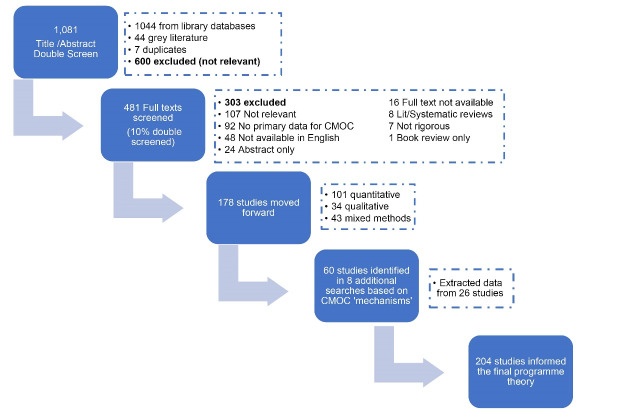
PRISMA Diagram for Formal and Additional Searches. Abbreviations: PRISMA, Preferred Reporting Items for Systematic Reviews and Meta-Analyses; CMOC, context-mechanism-outcome configuration.

 With a library specialist (DM), an initial search strategy (resulting in 1081 articles) was developed and conducted in line with the published protocol,^16^ from 2007 to May 2021 across 5 databases (EMBASE, CINAHL, MEDLINE, EconLit, Web of Science) and a separate grey literature search utilising ‘Google Scholar’ and the ‘Publish or Perish’ tool^19^ that retrieves and analyses citations based on total citations and h-index (Figure 2). The inclusion and exclusion criteria were largely based on relevance to the IPT and the need to be focused on health systems.

####  Inclusion Criteria

- The study must be related to health systems.
- The study must be focusing on the 2008 financial crisis.
- The focus must be on austerity and its impact/effects.
- The study must relate to key stakeholders – patients, public perception or engagement/disengagement with health service, staff, management, health officials, policy-makers, decision-makers.

####  Exclusion Criteria

- Studies focusing on the impact to industry eg, pharma.
- Studies focusing on private healthcare (ONLY) with no consideration for knock-on impact for public system.
- Exclude if focus of austerity is NOT focused on one of the following:
  ■ Public sector budgets/health system financing
  ■ Staff/workforce (and related issues eg, migration)
  ■ Access to care
  ■ Impact on population health/social issues (eg, homelessness, poverty, food insecurity)
  ■ Delivery of care/Innovation
  ■ Public reaction to health system/government
- Literature reviews that duplicate some or all data in primary studies (*Additional to published protocol*^16^).

Initially, there were no restrictions on study design, however the research, to be included, must have collected primary data or conducted secondary data analysis leading to insights relevant to this study. Literature reviews were subsequently excluded due to the duplication of data from already identified primary studies.

###  Stage 3. Data Screening and Extraction

 Search results were managed using Endnote X9, removing duplicates and locating full text articles. Title/abstract and full text screening were conducted utilising COVIDENCE review management tool.^20^ PF screened titles and abstracts for relevance to the research aims and objectives, resulting in 481 articles for full text screening based on inclusion and exclusion criteria and for trustworthiness, ie, sufficient evidence and rigour to warrant inclusion. The studies also needed to provide data that could be interpreted as context, mechanism or outcome, and if not were excluded on this basis. ST blind-screened 10% of title/abstracts and full text articles to ensure consistency. Disagreements at both stages were discussed and resolved by referring to the study protocol and IPT. 303 full-texts were excluded primarily based on relevance (35%) or because no primary data was collected to inform CMOC development (30%) (Figure 2). Departing from a traditional literature review, some excluded studies were still used to inform the research, based on information gleaned during full-text screening. Of the excluded studies, 93 were aligned with the IPT (although did not present any primary data), while 22 of those excluded studies further informed the IPT, raising new concepts such as the impact on training opportunities, inappropriate skill mix, weakened mental health and non-compliance with medication. After full text screening, 178 studies moved forward to stage four.

###  Stage 4. Data Analysis and Preliminary CMOC Development

 Data from included studies were extracted to Excel (quantitative) and NVivo release 1.6.1 (qualitative) for analysis. Descriptive analyses were conducted for quantitative data providing categorisation and overview of results, while deductive (based on IPT), inductive (data driven) and retroductive analysis was conducted on qualitative data. Retroduction refers to the process of identifying mechanisms or hidden causal forces that lead to observed patterns or themes within the data,^21^ with a view to developing middle range theories, in the form of CMOCs. Middle range theories involve interpretation of the primary data but the theories (CMOCs) are close enough to the original data to be testable.^22^ The process of retroduction was used to formulate five broad CMOCs, which were written and rewritten by the research team, as the CMOCs were compared and contrasted through a process of juxtaposition, reconciliation, adjudication and consolidation of the data.^23^ CMOC summaries were developed to demonstrate where the themes were drawn from in the identified literature (see Supplementary file 2). The preliminary CMOC analysis was presented to the stakeholder group on October 27, 2021, in order to confirm, refine or refute the middle-range theories.

###  Stage 5. Additional Literature Searches to Refine CMOCs 

 Additional iterative searches were conducted to further inform and refine the CMOCs emerging from the retroductive analysis, with a particular focus on the identified mechanisms and their theoretical origins. To achieve this, the searches drew from a broader literature base, without necessarily being limited to specific contextual factors such as ‘austerity’ or the financial crisis. Google Scholar was used to identify additional literature, utilising the Publish or Perish 7 tool.^19^ The terms used to search the literature were intentionally specific to the identified gaps in the formal search strategy:

1. Title (TI): transparency AND health;
2. TI: values AND health, Keywords (KW): burn-out OR austerity;
3. TI: autonomy OR empowerment AND healthcare professional OR doctor OR nurse;
4. TI: street level bureaucrats AND health; KW: resilience;
5. TI: moral distress AND health AND resilience;
6. TI: moral distress AND health; KW: austerity;
7. TI: ethical decisions AND health AND moral;
8. TI: health literacy OR financial AND health-seeking behaviour.

Sixty additional studies were screened for relevance, particularly with a view to identifying underlying theoretical frameworks to further refine CMOCs and develop a final programme theory. Data were extracted from 26 studies and through a process of abstraction, the relevant theories were integrated into the CMOCs, with summaries drawn up to indicate the relevant literature (Supplementary file 3).

###  Stage 6. Development of Refined CMOCs and Final Programme Theory

 The final stage of the process was to refine the preliminary CMOCs to develop a final programme theory, which was primarily conducted by PF, ST and LC. A clear chain of CMOCs was emerging from the analytical process, where the outcome from one CMOC presented the context for the next. The juxtaposition of the CMOCs revealed links between four of the five CMOCs, offering insights into governance changes and the knock-on effect on closely aligned proximal outcomes of interest. The fifth CMOC, based on analysis from the initial formal search, was orientated towards patient outcomes while the other four preliminary CMOCs were more aligned with health systems outcomes and the aims and objectives of the review. It was therefore decided to exclude the fifth CMOC from the refined CMOCs and final programme theory, as it was beyond the scope of this review. A final stakeholder meeting, involving disciplines from economics, social sciences, health sciences, policy analysis, and implementation science, was held on May 5, 2022 to confirm the rationale for this decision and to discuss the refined CMOCs and final programme theory.

## Results

###  Study Characteristics

 The initial search and screening process resulted in 178 studies for inclusion in the first stage of analysis (developing preliminary CMOCs). 101 studies were quantitative in nature, 34 qualitative and 42 mixed methods. Two-thirds (67%) of studies related to Southern European countries (Spain, n = 22; Portugal, n = 17; Italy, n = 11; Greece, n = 27; and Cyprus, n = 1) and other European countries particularly affected by austerity (Ireland, n = 15; UK, n = 15; and Iceland, n = 5; Multiple, n = 6). The remaining third was distributed internationally (See Supplementary file 4).

###  Quantitative Data

**Figure 3:**
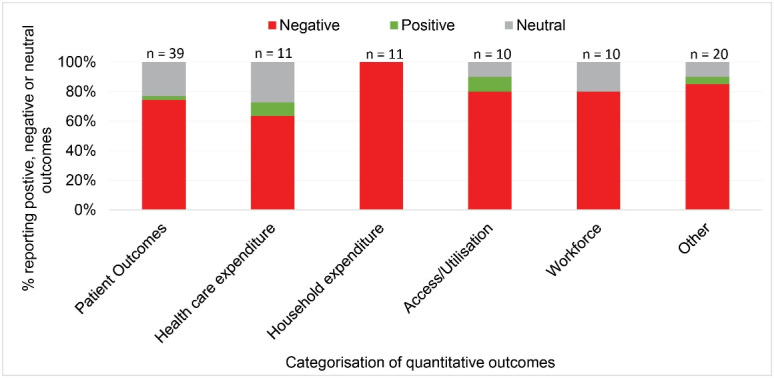
Quantitative Outcomes and Impact of Economic Crisis.

 The quantitative studies provided insights into the proximal outcomes of interest during the years that followed the economic crisis, while also providing insights into the changing contexts for the system, workforce and service users. Six main categories included patient outcomes (n = 39), healthcare expenditure (n = 11), household expenditure (n = 11), access/utilisation (n = 10), workforce (n = 10) and other (n = 20) (Figure 3). The other category represented a range of outcomes such as public perception, service delivery, prescription medication and infectious disease. The economic crisis predominantly resulted in negative outcomes reported (79%), with 17% of studies reporting neutral outcomes and 4% positive.

 Examples of negative patient outcomes included increased mortality associated with reduced resources and quality of care (staff, infrastructure, delayed discharges)^24-29^; increased suicide/suicidal attempts^30-33^; increased mental health problems^34-36^; and unmet need^37-41^ (see Supplementary file 4). These adverse impacts were often directly or indirectly associated with decreased healthcare expenditure^42-47^; increased household expenditure often associated with increased out-of-pocket payments for healthcare^48-55^; reduced access to or utilisation of care^56-62^; and reduced and strained workforce.^63-71^ The positive results included reduced inequalities in male amenable mortality due to increased health expenditure in deprived areas,^72^ achievement of long-standing policy goals of increased public-patient discharges resulting in reimbursement – due to introduction of information systems,^73^ and decreased prescription medication use due to introduction of co-payments^74^ – although the latter was reported positively in terms of achieving policy goals, the consequences of these changes were unknown and possibly negative in the longer term.

###  Qualitative Data

 Based on the emerging outcomes of particular interest, the qualitative and mixed methods studies (n = 64) were read and re-read, and sorted into nine categories. Using NVivo analysis software, inductive, deductive and retroductive coding focussed on studies (n = 36) that provided insights into the hidden mechanisms that were potentially influencing the outcomes of interest reported across the body of work, namely: workforce (n = 13), access to care (n = 10), decision-making (n = 8), impact (n = 3) and service delivery (n = 2).

###  Realist Analysis of Quantitative and Qualitative Data

 The realist analysis of quantitative and qualitative data led to the development of five preliminary CMOCs. This included presentation of results to stakeholders, followed by in-depth discussion. Taking this feedback on board, the CMOCs were written and re-written, debated and discussed among the core research team (PF, ST, LC, SVB, SBur, SBar and JC), drawing on available evidence and multi-disciplinary expertise. The five preliminary CMOCs can be seen in Box 1, while the fifth (patient-focused) CMOC was later excluded from the refined CMOCs and final programme theory.

**Box 1. Preliminary Context-Mechanism-Outcome Configurations**

** CMOC 1. Top-Down Governance**

The austerity era following the 2008 financial crisis set a context where health systems decision-making was highly influenced by outside agents (eg, Troika).^75-79^ The lack of transparency* about these outside influences, compounded by poor communication from policy-makers/management and lack of co-production with frontline staff,^80-82^ led to a lack of ownership and buy-in from those delivering care and a distrust of the decision-making agenda.^80,83,84^

** CMOC 2. Powerless and Detached**

In the context where achieving efficiencies was the top of the agenda, monitoring was increased, for example, the introduction of information systems to track activity and spending.^76,82,84-87^ Health professionals perceived a loss of autonomy*^77,85,87-90^ and decision-making power*,^77,84^ leading to a sense of powerlessness^86,91^ and detachment and ultimately a resistance to change and conflict between front line workers and policy decision makers/management.^76,81,84^

** CMOC 3. Perceived Value Shift**

In context of restrictive fiscal policies (staffing, consumables, treatment options, available time with patient), a perceived value shift* is evident for health professionals, from patient-focused to economic,^84,87,88,90,92^ with long-term consequences resulting in a diminished view of the profession, apathy and burnt-out among health professionals.^81,84,87,89,93-96^

** CMOC 4. Working the System (Access)**

A new context of street-level bureaucracy emerged where health professionals began to circumvent policy to deliver care (legal, informal referrals, treat regardless of ability to pay)^77,80,94,97-100^ due to a sense of professional/moral duty or ethical decision-making*, solidarity with patients or fellow health professionals.^90,94,97,101^ Ultimately this led to strain on frontline workers, increased emergency department use but indicative of more stable health outcomes than originally predicted.^102^

** CMOC 5. Health-Seeking Behaviour Change**

In the context of mounting financial concerns and pressures for members of the public with the introduction/increase in out-of-pocket payments, health-seeking behaviour change*,^100,103^ compounded by issues related to health literacy*,^75,92,99,103,104^ led to reduction in primary care usage, increased emergency care, medication mismanagement, delayed treatment.^6,75,80,99,105^

-----------------

Abbreviation: CMOC, context-mechanism-outcome configuration.

* Mechanisms that informed additional searches.

###  Refined Context-Mechanism-Outcome Configurations 

**Figure 4:**
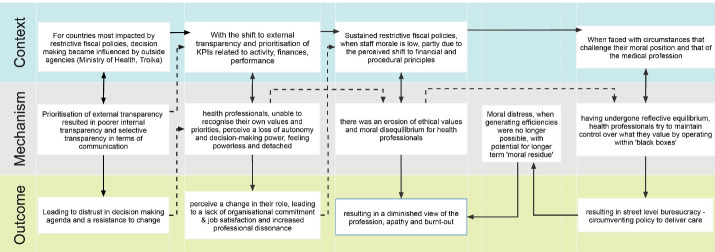
Refined Context-Mechanism-Outcome Configurations and Their Interactions. Abbreviation: KPIs, key performance indicators.

 At this point in the review, the analysis began to focus on workforce resilience, given the richness of the available data within this category. As previously indicated, eight mechanism-driven additional searches were conducted, primarily based on the underlying mechanisms identified through the formal search strategy, and the preliminary CMOCs developed. These iterative searches sought to identify additional data from a broader literature base, while also identifying the theoretical basis of emerging themes (Box 2). Theory-driven data extraction, followed by further iterative rounds of realist analysis, led to a set of five refined CMOCs, discussed below and graphically represented in Figure 4. As indicated previously, the fifth preliminary CMOC was excluded from the refined CMOCs.

**Box 2. Theoretical Concepts Informing Refined Context-Mechanism-Outcome Configurations**

**Transparency**

*Internal Transparenc*y: “an outcome of communication behaviors within an organization that reflects the degree to which employees have access to the information requisite for their responsibilities.”^106^

*External Transparency*: “is communication to the environment outside the organization. It aims at ensuring outsiders, normally a specific group, are aware of certain organizational activities.”^106^

*Selective Transparency:* “is generated by key performance indicators, and this enables managers to control the activities of the managed.”^107^

**Reflective Equilibrium**

A method that “is used in ethical decision-making to reflect on a perspective or judgment in order to reach a justified moral position between competing moral judgments. In reflective equilibrium: …we ‘test’ various parts of our system of beliefs against the other beliefs we hold, looking for ways in which some of these beliefs support others, seeking coherence among the widest set of beliefs, and revising and refining them at all levels when challenges to some arise from others…a person who holds a principle or judgment in reflective equilibrium with other relevant beliefs can be said to be justified in believing that principle or judgment.”^108^

**Moral Disequilibrium**

“Moral disequilibrium results when health professionals, faced with challenges to their values and moral integrity, are unable to adapt to maintain their moral integrity. Moral disequilibrium is part of the everyday experience of being a health professional, and like all experiences will vary from person to person and in intensity, from mild (eg, moral discomfort) to severe (eg, moral distress, moral injury).”^109^

**Moral Distress**

“Moral distress is the painful psychological disequilibrium that results from recognizing the ethically appropriate action, yet not taking it, because of such obstacles as lack of time, supervisory reluctance, an inhibiting medical power structure, institution policy, or legal considerations.”^110^

**Moral Residue**

“Past distress may remain a dormant part of a person’s subjectivity and re-emerge or become (re)enacted in the narrations of those past distressing experiences (this can be related to what Hardingham [2004] called ‘moral residue’).”^111^

**Lipsky’s Street Level Bureaucrats – Interpreted for Healthcare Professionals**

“Lipsky’s theory: (1) SLBs are well-intentioned and at least initially committed to public service. Over time the constraints of their work environments may erode their ideals and negatively impact service delivery; (2) SLBs operate within severely resource-constrained environments where they face a paucity of personal, organisational, and community resources; (3) SLBs in their client-facing role end up interpreting policies and therefore shape what service delivery and public policy become on the ground. Because SLBs often need to justly apply meagre resources with policies that may be conflicting, their role makes discretion inevitable; and (4) As part of their service patterns, SLBs develop coping mechanisms to deal with the pressures and the resource constraints of their jobs.”^112^

**Black Box Activity**

“There are therefore black boxes (Glanville, 2003) within the system where action is taken independently of the manager’s control.…Within black boxes, interpersonal and professional practices arise that maintain the viability of the organization by absorbing the variety generated by the activities that occur within the black box… black boxes enable multiple conversations to take place, generating multiple perspectives and interventions with which to meet the challenges generated by continual external change.”^107^

-----------------

Abbreviation: SLBs, Street Level Bureaucrat.

####  Refined CMOC 1. Top-down Governance and Transparency

 In the aftermath of the financial crisis, some countries were more burdened by the restrictive fiscal policies that followed, for example, Ireland, Greece, Spain and Portugal, where decision-making was increasingly influenced by outside agents. In the case of Ireland, at a national level the Health Service Executive became increasingly influenced by government including the Department of Health and the Department of Public Expenditure and Reform. At a European level, Troika represented a decision group formed by the European Commission, European Central Bank and the International Monetary Fund. These restrictive fiscal policies had a direct impact on national policy and practice, with external agents closely monitoring progress to meet strict measures to reduce costs, increase efficiency, while continuing to deliver care.

 In order to demonstrate progress to external agents there was a shift towards external transparency,^106^ with performance monitoring and data collection highly influenced by the interests of these external agents.^76,85,88,94,103,113-115^ This had a diminishing effect on internal transparency – the degree to which employees have access to information necessary to perform their duties^106^ – which was previously driven by the interests and priorities of internal agents (healthcare staff). The lack of transparency was further compounded by poor communication from management and a lack of co-production with frontline staff – in terms of deciding what activities or efficiencies to prioritise in order to meet the targets set. On the contrary, the managerial-led prioritisation led to selective transparency – enabling management to control the activities of frontline staff.^107^

 These developments led to a distrust in the decision-making process and a resistance to change among health professionals. In such low-trust organisations a range of behaviours are predictably common, such as high absenteeism, limited learning, low accountability, low creativity and reactionary thinking.^116^

 “*As a doctor, I find it hard to accept that reporting our work to outside organizations is more important than finding the right treatment for a patient. It seems that nowadays, everything is more important than the patient”*^84^ (CMOC 1-3).

####  Refined CMOC 2. Perceived Value Shift, Loss of Autonomy and Decision-Making Power 

 The cultural shift, within the working environment, to external transparency and the prioritisation of key performance indicators, set by outside agents, related (for example) to activity, finances and performance, became a contextual factor in the chain of events following the onset of the economic crisis.

 Healthcare professionals were unable to see their input, values and interests reflected in the dialogue and data flows, reflecting instead situational values – specific to austerity, changes in the balance of organised forces, the interests of influential external agents and ideological positions.^117^ With diminished internal transparency, health professionals perceived a change in their role and an associated perceived loss of autonomy and decision-making power, feeling powerless and detached.

 This led to a lack of organisational commitment and job satisfaction^116^ and increased professional dissonance – a discomfort arising from conflicting professional values and job expectations.^118^

 “*The monitoring of public hospitals’ performance and of drugs prescription through compulsory electronic procedures intended to limit doctor’s autonomy”*^85^ (CMOC 1 and 2).

 “*Such constraints dictated their ability and capacity to perform and deliver their services, suggesting tension between professional autonomy and organizational structure. Consequently, austerity appears to have changed the participants’ perception of their autonomy. Their ability to make decisions freely was curtailed by the requirement to meet targets and create cost efficiency savings”*^90^ (CMOC 1-3).

 “*The healthcare providers felt powerless about supporting migrant healthcare with such low capacity in the system. They felt that they were ineffective with regard to their ability to bring changes to the system to improve migrant healthcare. They thought themselves as being the final recipients of political decisions without any scope for active participation in these decision-making processes”*^91^ (CMOC 2 and 4).

####  Refined CMOC 3. Low Staff Morale and Moral Disequilibrium

 With low staff morale amidst sustained restrictive fiscal policies, such as rationing of staff, consumables, treatment options and available time with patients, alongside the perceived shift to financial and procedural principles, there was an erosion of ethical values for healthcare professionals who were experiencing moral disequilibrium – unable to maintain their values and moral integrity.^106,109^ This resulted in a diminished view of the profession, apathy and burnout.

 “*I think in times of austerity the ethics of decision-making becomes even more important. Because very often one is having to make difficult decisions between spending areas or projects and so it’s important when one is making most decisions one takes into account what is equitable” *Member of European Parliament^101^ (CMOC 2 and 3).

 “*…trying to keep patients in hospital longer while trying to fulfil your duty of care…making sure they get the right treatment within your control…I think every aspect, on every front, there are challenges there that weren’t [there] five or ten years ago.”*^90^

 “*Others responded by abusing their leave; a senior manager remarked on the increase in absenteeism and sick leave. One staff member admitted: ‘I just go to the doctor and book myself off so that I can rest.’”*^81^

####  Refined CMOC 4. Working the System and “Black Boxes” 

 When faced with circumstances that challenged their moral position and that of the medical profession, such as policies that excluded or limited access to care for vulnerable populations, health professionals underwent a process of reflective equilibrium ie, ethical decision-making that tests system beliefs with other beliefs sets.^108^ In an effort to maintain control of what they value, health professionals operated within black boxes, utilising professional discretion – where action is taken independently without the approval (or sometimes the knowledge of management), in an effort to balance system needs with service user needs.^107^

 This led to street level bureaucracy (SLB)– a sociological concept of how public servants interpret government policy (based on organisational context and personal interests) and subsequently administer their duties and implement policy,^112,119,120^ in the case of this review revealing how health professionals circumvented policy to deliver care.^120^ This included formal interventions, such as legal challenges; or more informal approaches such as referrals to friends/colleagues outside the official system; or treating patients regardless of their ability to pay. Operating within black boxes also resulted in management becoming removed and less aware of the issues within units around them,^106^ further impacting poor communication outlined in CMOC 1.

 “*I mean, I feel I’m being interfered and manoeuvred, but actually when a patient is sitting in front of me, if I need to operate on that patient, a lack of funding or anything like that has not stopped me from doing it – I’ve always been able to do that”*^77^ (CMOC 1-4).

 “*l[the girl] couldn’t breathe (…) they didn’t want to give her an appointment because she didn’t have a health card (…)[another nurse intervened] you can’t refuse the girl medical treatment…When that law was passed [RDL 16/2012] [restricting access to care] (…) in the outpatient centre we had a meeting and said that we’d attend to everyone”*^100^ (CMOC 3 and 4).

 “*Oh, it’s a no-brainer for me, I see everyone, that’s a given, and we try to help everybody here…I believe, myself and all my colleagues, that no one is going to be kept outside those doors and no one will remain unattended”*^97^ (CMOC 4).

####  Refined CMOC 5 - Street Level Bureaucracy and Moral Distress

 SLB presented the context for the final mechanism. Health professionals experience moral distress – a painful psychological imbalance resulting from not being in a position to take ethically appropriate action, because in the case of austerity, generating efficiencies are no longer possible.^110,112,121,122^ Once again, this results in a diminished view of the profession, apathy and burnout, starting to generate a cyclical and destructive interplay between CMOCs.^109^ Moreover, if moral distress is not treated appropriately, can result in ‘moral residue,’ where past moral distress can lie dormant until the next shock, when it resurfaces unexpectedly.^111,121^

###  Final Programme Theory 

 While the final programme theory is built upon five separate, but interconnected, CMOCs (Figure 4), all contextual factors relate to austerity-driven policies and their knock-on effect on the organisational functioning of health systems. With this in mind, the countries with which this programme theory relate to are those most impacted by efficiency-driven restrictive fiscal policies and the knock-on effects that these fiscal policies have on the health system and specifically components that relate to organisation theory, namely organisation design, leadership, managerial strategies, culture, communication, behaviours, motivation and efficiency.^123^ The CMOCs outlined in the results section demonstrate how policy decisions can impact each of these organisational components by altering managerial and associated communication strategies, while moving from a culture of communication and policy implementation that is primarily driven by internal transparency to one that is influenced by external transparency. This has the potential to create a real or perceived shift towards the fiscal interests of agents outside the health sector, rather than the values and interests of health professionals working within the system to deliver best practice, and who are striving to continuously maintain or improve quality of care and patient outcomes.

 The organisational and working environment shifted towards increased scrutiny on day-to-day activities, performance metrics and financial efficiencies, which was associated with growing discontent among staff who were trying to balance the needs of the organisation with the needs of service users and patients. In parallel health professionals were attempting to maintain a set of professional and personal values that increasingly appear at odds with policy decisions and the realities of implementing such policies within sustained and increasing restrictive working conditions (rationing of staff, consumables, treatment options). The juxtaposition of a diminished view of the profession, due to the aforementioned tensions, an inability to provide high-quality care in an equitable, needs-led way, alongside stagnant or degraded working pay and conditions, led to moral distress. Moral distress, if unattended can turn to moral residue, forging legacies that may adversely impact resilience when faced with future shocks or challenges. Staff can, effectively be psychologically triggered by past experiences impacting how they perceive and respond to future challenges. Pre-emptive action to mitigate breakdown in communication, lack of ownership in policy and practice decisions, low job satisfaction, professional dissonance, and poor quality of care has the potential to avoid situations where health professionals experience moral disequilibrium or moral distress. These concepts will be discussed within the context of the wider international literature, followed by recommendations for policy and practice.

## Discussion

 In practice, insufficient attention has been paid to linking recovery and learning from one shock to preparedness for the next. Understanding what to be prepared for and subsequently how best to be prepared requires a nuanced approach based on a deep understanding of how and why health systems are impacted by the shock. Traditionally, once a shock has passed, decision-makers understandably tend to focus once again on dealing with day-to-day system stresses. However, health system resilience is not time bound or limited to discrete shocks, with legacies carried from one shock to another. As revealed in this review, the health workforce was a key priority in the academic literature following the 2008 economic crisis and once again, the COVID-19 pandemic highlighted the vulnerability of the health workforce, having to work under extreme, uncharted and unrelenting conditions.^124,125^ The findings of this review are particularly relevant at this time, given that austerity is becoming a default response after the acute phase of the COVID-19 pandemic,^3^ with the propensity for global financial markets to, once again, influence national policies, including health.

 This review demonstrated how and why proximal outcomes closely related to the 2008 economic crisis – such as lack of ownership and distrust in policies, resistance to change, conflict with management, low morale, staff burnout and apathy – can potentially have a lasting legacy for health system performance, particularly in terms of resilience for future shocks. The CMOCs uncovered hidden mechanisms at play, such as a shift from internal to external transparency, undermining health workers’ role in priority setting, a workforce that felt powerless and detached, a health system that was perceived to have lost its patient-focused values in favour of economic targets and efficiencies, and a workforce who felt challenged by this value shift, seemingly resulting in first moral disequilibrium and finally moral distress – with the potential for moral residue. Below we discuss how such negative outcomes from shocks can be mitigated.

###  Mitigating Moral Distress and Moral Residue

 Legacies from previous shocks that impede workforce resilience can remain invisible or hidden, especially for those with lived experience at the frontline. One such concept relates to ‘moral residue,’ where past moral distress can lie dormant until the next shock, when it resurfaces unexpectedly.^111^ When moral distress re-emerges it can trigger reactions to policy decisions and practice-changes that may be influenced from past experiences rather than purely related to present day circumstances. In a general sense, moral distress within the workforce can be mitigated in several ways, by promoting and nurturing passion and job satisfaction,^109^ ‘good enough practice’ (given the supply and demand constraints),^118^ having access to information, receiving support, having access to resources necessary to do the job, and having the opportunity to learn and grow.^116^ More targeted strategies can also be adopted to mitigate shock-specific challenges, considering the three causes of moral distress – individual, institutional or other external factors.^126^ During COVID-19, for example, individual factors included: perceptions of ability/skills to manage; physical, emotional and financial resources to care for both their own families and patients. Institutional factors included: availability of personal protective equipment, beds and respirators, while other external factors included: restrictive health regulations and changing and uncertain national public health policies.^126^ Targeted strategies can be implemented to achieve moral equilibrium for the workforce, when faced with challenges to their values and moral integrity.

###  Achieving Moral Equilibrium

 Ong, proposed a three-phase framework to achieve moral equilibrium, first by identifying moral disequilibrium and the moral values involved, second by finding a resolution to achieve ‘good enough’ moral equilibrium, and finally an evolutionary phase where there is a growth in moral understanding and moral resilience.^109^ At an organisational level, when substantial ethical and social values are not explicitly named as guiding principles in policy development, this can impact accountability, transparency, consistency, and public, political and professional understanding of decisions made.^127^ One way to achieve professional fulfillment, vital in achieving high quality system improvements, is to facilitate ‘value shops’ whereby shared values are generated by mobilising resources and activities to resolve particular challenges within the system.^128^ These guiding principles and shared values then need to be translated into tangible implementation strategies, allowing managers to demonstrate their shared understanding and frontline workers to recognise and be reassured that co-production at policy level is being translated into practice. At an individual level however, moral understanding can be blocked by lack of self-confidence, doubts about self-knowledge, fears, personality, lack of courage to challenge status-quo, fear of conflict and a sense of powerlessness.^109^ Creating a workplace environment that mitigates these organisational and individual challenges has the potential to promote constructive, co-produced and well-communicated decisions that can be made between policy-makers, management instructing implementation and frontline staff implementing policy.

###  Cyclical Flow of Information 

 The concept of transparent communication is another key mitigation strategy for many of the legacies identified through this review, for example lack of ownership and distrust of decisions made; resistance to change and conflict; and perceived value shift at management level. When considering communication breakdown, much of the literature tends to focus on a top-down flow of information and communication. In line with the idea of top down information flow, Griffiths posits that selective transparency is generated by key performance indicators, enabling managers to control the activities of the managed, changing the balance between specified autonomy and imposed transparency.^107^ In one sense, governments may have been trying to scrutinise black box activity of frontline workers, exposing details of a functioning system that get lost in day-to-day operations, in an effort to identify potential inefficiencies. However, information flows in multiple directions both downstream and upstream. The actions taken by government to reveal black box activity inadvertently resulted in reduced internal transparency (information required by employees to do their job) and increased black box activity, as theorised in this review – when frontline workers circumvented policy to deliver care. This in effect potentially weakened communication flow upstream, in addition to an already weakened downstream information flow, as widely acknowledged in the literature. Furthermore, a risk of opening up black boxes is to reduce the flexibility that is provided by the opaqueness of the boxes – allowing parallel conversations to take place, generating multiple views and actionable tasks with which to meet the day-to-day implementation challenges arising.^107^ This process is crucial to sustain a complex social system and therefore must be respected. To facilitate this respectful interaction and to further mitigate the breakdown in communication, policy and practice decisions need to be co-produced, focusing on enhanced communication flow, and perhaps more importantly, enhanced understanding of the information communicated.^106^

###  Empowerment Through Internal Transparency

 Enhanced internal transparency can be achieved by ‘layering’ access to information, ensuring that people get the information they need to do their job, while avoiding information overload or potential security breaches. Indeed, according to Kanter’s theory of organisational empowerment, it is the mandate of management to ensure employees have the necessary information, support and resources.^116^ Furthermore, information needs to be meaningful and useful, which sometimes requires intermediaries to decipher the information for non-experts to understand.^106^ To date, the literature often focuses on co-production between frontline staff and patients, or may acknowledge how decision-makers try to gather as much information as possible to inform their decisions – by listening to patients, colleagues, professionals,^101^ but there is little detail on the quality or generalisability of the information gathered to inform these decisions. Furthermore, the literature focuses less on how decisions are co-produced with frontline staff, who arguably have the most rounded view of organisation and patient needs. To establish trust, employees have to have faith that organisational action will benefit staff and end users, with trust being built on open communication – sharing critical information; perceptions and feelings; and co-produced decisions.^116^ Further research is required to develop effective communication strategies that are co-produced by both internal and external agents, while upholding the agreed ethical and social values that lead to the desired organisational and patient outcomes.

###  Strengths and Limitations

 Although not a systematic review, the process was very systematic and transparent, with detailed recording of each stage of the review process, including recording and transcriptions of three sub-group meetings with stakeholders from various academic and professional disciplines, alongside PPI representation. Screening was undertaken by two reviewers and the CMOCs were developed collaboratively with several authors with differing disciplines (economics, social science, health sciences), and further validated by broader stakeholder involvement. Another strength of the review was the scale, with over 200 studies informing the preliminary and refined CMOCs.

 Limitations of this study relate to the broad nature of the research questions, having to focus on one specific aspect of the literature (workforce), that being the area with the most peer-reviewed published material to inform a realist analysis. As a result, not all proximal outcomes could be explored and developed fully, allowing scope for further research and realist analysis in these areas.

 While the evidence gathered was varied geographically, the refined CMOCs focused on literature from European countries most adversely impacted by austerity following the 2008 financial crisis, namely Greece, Ireland, Italy, Spain, Portugal, and the United Kingdom, therefore the findings may not be generalisable for all countries, particularly low- and middle-income countries.

 The intention of a realist review is to develop testable theories; however the plausibility of the findings could be tested with further empirical data collection, which is planned as part of the wider RESTORE project, under which this research was conducted.

 Finally, while every effort was made to include a broad range of stakeholders, it is possible that a different set of disciplines may have interpreted the findings differently. Nevertheless, the findings are closely related to a large body of work spanning over a decade, and the findings should provoke further debate and discussion among a broad range of disciplines.

###  Recommendations

 Building on the refined CMOCs and final programme theory presented in this paper, recommendations are outlined below to provide guidance for how health systems can be better prepared to respond to shocks in order to minimise potential adverse effects.

1. If sustained restrictive fiscal policies are required, open and transparent communication is key – with clear and agreed ethical and social values guiding policy and practice – to encourage buy-in and ownership of decisions.
2. When service delivery rationing and efficiencies are required, communication strategies should be developed to facilitate information flow in both directions, providing frontline workers with the necessary information to do their job, while offering decision-makers insights into the challenges faced at the frontline due to changing context (rationing).
3. When communication is facilitated in a useful and meaningful way, flowing both downstream and upstream, this will allow deeper understanding and nuanced solutions that are aligned with the guiding values and principles, ensuring frontline workers recognise their ongoing dual role in policy and practice.
4. When decisions makers, managers and frontline staff have a shared understanding and co-produced value-set, then policy implementation strategies are more likely to be successful because there is not a perceived conflict between the end-goals of each group.
5. When frontline activity requires flexibility and agility to respond to diverse and changing needs, managers should expect and trust the need for professional discretion (‘black box’ activity), with management facilitating shared ownership and values to meet both organisational and patient outcomes.
6. When staff are dealing with unprecedented circumstances and all efforts are focused on delivering care, management should focus on identifying causes of moral distress in order to determine solutions that allow good enough outcomes given the strained circumstances. These may differ from the normal best practice.

## Conclusion

 Health worker resilience is clearly impacted by shocks, with legacies carried from one shock to another. This realist review demonstrates how resilience can be undermined by poorly communicated and non-transparent policy implementation, leading to low morale, professional dissonance and moral distress, as health workers try to maintain the standards previously associated with a thriving and well-resourced health system, while in parallel implementing restrictive fiscal policy in light of a rapidly deteriorating economic conditions. By unpacking the workforce response, in countries most acutely impacted by the era of austerity, this review reveals the importance of transparent, open communication, in addition to co-produced policies in order to avoid scenarios that can be detrimental to health system resilience. This review provides insights for future policy-makers and analysts alike, in order to robustly design policy development and implementation strategies that harnesses the strengths and values of all stakeholders, from government, to health workers and ultimately end-users of the health system.

## Acknowledgements

 The authors would like to thank various stakeholders, who were critical to the realist review process - providing rich and insightful feedback, particularly Professor Carolyn Tuohy – University of Toronto; Professor Susan Smith – Trinity College Dublin; and Ms. Laura de Burca – PPI representative. We would also like to acknowledge the Health Research Board for funding this research as part of the RESTORE project.

## Ethical issues

 Not applicable.

## Competing interests

 Authors declare that they have no competing interests.

## Authors’ contributions

 PF: Conception and design; acquisition of data; analysis and interpretation of data; drafting of the manuscript; administrative, technical, or material support. LC: Conception and design; analysis and interpretation of data; critical revision of the manuscript for important intellectual content. SVB: conception and design; analysis and interpretation of data; critical revision of the manuscript for important intellectual content. SBar: Conception and design; analysis and interpretation of data; critical revision of the manuscript for important intellectual content. SBur: Conception and design; analysis and interpretation of data; critical revision of the manuscript for important intellectual content. JC: Conception and design; analysis and interpretation of data; critical revision of the manuscript for important intellectual content. RS: Conception and design; critical revision of the manuscript for important intellectual content. DM: Conception and design; acquisition of data. ST: Conception and design; analysis and interpretation of data; critical revision of the manuscript for important intellectual content; obtaining funding; supervision.

## Funding

 This work was supported by the Health Research Board [grant number RLA-2020-001]. The Health Research Board had no role in the design and conduct of the study, data collection, data management, data analysis and interpretation, preparation, review and approval of the manuscript.

## Supplementary files


Supplementary file 1. Developing If, Then, Because Statements.

Supplementary file 2. Preliminary CMOC Summaries.

Supplementary file 3. Theoretical Lines of Enquiry From Additional Searches.

Supplementary file 4. Study Descriptives.
