# Supplementary material for: How International Health System Austerity Responses to the 2008 Financial Crisis Impacted Health System and Workforce Resilience – A Realist Review
Source: Int J Health Policy Manag. 2022 Dec 24;12:7420. doi: 10.34172/ijhpm.2022.7420 (PMC10125082; doi:10.34172/ijhpm.2022.7420)
Supplement: Supplementary file 1 — Developing If, Then, Because Statements. [file ijhpm-12-7420-s001.pdf]

**Article title:** How International Health System Austerity Responses to the 2008 Financial Crisis Impacted Health System and Workforce Resilience – A Realist Review

**Journal name:** International Journal of Health Policy and Management (IJHPM)

**Authors' information**: Padraic Fleming[1]*, Louise Caffrey[2], Sara Van Belle[3], Sarah Barry[1], Sara Burke[1], Jacki Conway[4], Rikke Siersbaek[1], David Mockler[5], Steve Thomas[1]

[1]Centre for Health Policy and Management, Trinity College Dublin, The University of Dublin, Dublin 2, Ireland.

[2]School of Social Work and Social Policy, Trinity College Dublin, The University of Dublin, College Green, Dublin 2, Ireland.

[3]FWO, Institute of Tropical Medicine, Antwerp, Belgium.

[4]Everlake, 5 Marine Terrace, Dun Laoghaire, Dublin, Ireland.

[5]Library Reader Services, Trinity College Dublin, The University of Dublin, St James Hospital, Dublin 8, Ireland.

(*Corresponding author: Padraic.fleming@tcd.ie)

**Supplementary file 1**. Developing If, Then, Because Statements

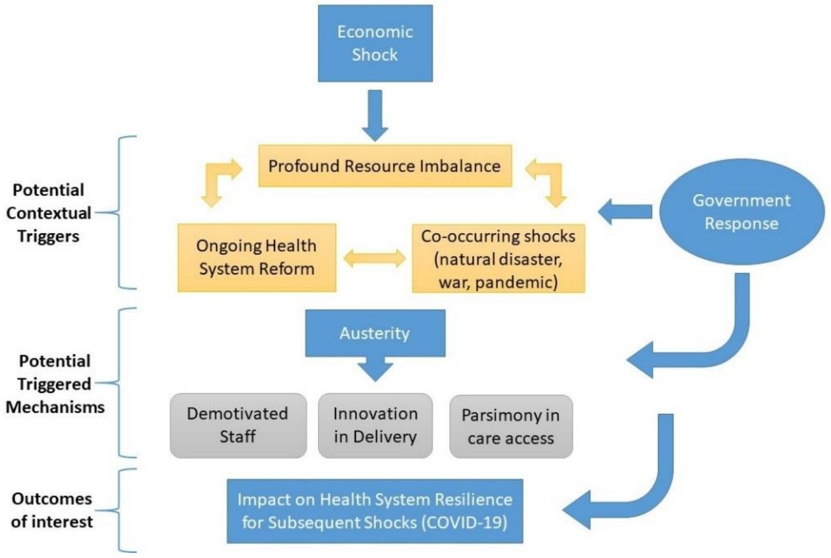


Figure S1 - Initial Programme Theory

Using realist language: Interventions are introduced to change context. I propose that Austerity is the 'interventional strategy' adopted by governments to change the context.  Changing context, which then (intentionally or unintentionally) triggers new mechanisms, that lead to particular outcomes (proximal outcomes in the first instance) and ultimately to the distal outcome of interest. So we need to understand how, when, for whom, to what extent did austerity change context.

Start with the outcome of interest and work backwards. Our outcome of interest is twofold. 1. More resilient health system and 2. Less resilient health system.  This is out distal outcome. The following statements relate to proximal outcomes.

We also need to consider time, which of these statements come first and how do they influence subsequent statements or are all these completely independent?

First stage of our screening is to decide which CMOCs stay in our initial programme theory and which are rejected as less relevant, based on the available evidence.

# If, then, because statements

Overarching context is reduced public sector budgets due to austerity

## Human resources

Austerity leads to reduced public sector budgets and

- Reductions in salary
- reduced staffing/increase workload,
- loss of leadership/senior personnel
- adverse impact on training / education of workforce (Glasper 2015)
- inappropriate skill mix (e.g. too many doctors and not enough nurses) (Notara 2010)

which may cause demotivation/disengagement/burnout of staff

which leads to

- less flexibility,
- lower productivity,
- poorer performance,
- less willingness to innovate,
- lower quality of care

in shocks

ITB1 - **If** [salaries are reduced; workload is increased; staff are lost] **then** there will be [less flexibility; lower productivity; poorer performance; less willingness to innovate; lower quality of care] **because** [staff are demotivated; disengaged; burnt out].

***Note based on feedback from PPI representatives:***

Weakened mental health leads to be more exposed to next shock (Antunes, 2019)

## Access for households

Austerity leads to reduced public sector budgets and

- transfer of access/treatment costs onto households
- Reduced financial protection of vulnerable groups (sick, old, poor) through higher co-pays

Which leads to restrictions in accessing healthcare

- Worse care for vulnerable groups in a shock
- Higher inequalities around accessing essential care in a shock
- Unequal outcomes in a shock

- Increased mortality for older populations (85+)
- Increased mortality in general (Rajimil 2019)
- Migrants locked out of health system (in a number of papers tbc, Theocharous 2014)

ITB2 - **If** [access to healthcare / treatment costs is transferred to households; financial protection for vulnerable groups is removed] **then** there will be [worse care for vulnerable groups; Higher inequalities around accessing essential care; Unequal outcomes] **because** [fewer people can easily access care when needed; people are forced onto long public waiting lists; ignore symptoms and don't access care].

## Population Health

Austerity leads to job losses, reduced income, inaccessible and unaffordable healthcare, and reduced social protections, social inequalities (many full texts e.g. Mattheys)

This leads to:

- Increased poverty (people with disabilities and children are being disproportionately affected – Taylor-Robinson, 2014)
- Increased homelessness
- Increased mental health problems
- Increased suicide / attempted suicide rates/self-harm
- Increased morbidity (Increase in communicable diseases, Theocharous 2014) /mortality (Rajimil 2019)
- Non-compliance with medication because it's too expensive (Fraeyman 2015)
- Increase in communicable diseases (didn't capture author)… (and financial constraints mean there's inability to have preventative measures in place, Meletis 2015)
- Increased probability of Lower birth weights (Olafsson, 2016)

Which then means that the health system is under more pressure, since these populations tend to have higher health needs and dependency on emergency departments (e.g. in the case of homelessness - risks of infectious disease, physical harm, food insecurity, multiple morbidities and premature mortality).

ITB3 - **If** [healthcare is made inaccessible or unaffordable through reduced income through job losses, coupled with reduced social protections] **then** [the health system will experience more pressure in terms of demand, particularly in emergency departments], **because** [the population will experience increases in poverty, homelessness, mental health problems and increased suicide/attempted suicide and self-harm].

ITB4 - **If** [priorities are made in terms of health care delivery; synergies between services slow down or cease (e.g. registration, drug procurement and supply, laboratory network, human resources, and financing] **then** [diseases of inequality, e.g. HIV/AIDS, and diseases of poverty e.g. tuberculosis will be exacerbated] **because** [integrated programme delivery is neglected i.e. epidemiological surveillance, programme monitoring and evaluation, community awareness of health-seeking behaviour, risk behaviour modification, infection control, treatment scale-up (first-line treatment regimens), drug-resistance surveillance, containing and countering drug-resistance (second-line

treatment regimens), research and development, global advocacy and global partnership]. (Maher 2010 – Metrics review)

## Innovation

Austerity leads to reduced public sector budgets and

- Better efficiency and/or
- More innovation in service delivery
- Cost savings and less financial waste through generic pharmaceuticals (Nune, 2020)
- Move towards international best practice for reimbursement of public hospitals based on internationally recognised diagnosis related groups (DRGs) (Polyzos, 2013).

And then two competing or conflicting pathways:

- Less slack or room for efficiencies in a subsequent shock and/or
- Culture of innovation enhanced allowing a better response to a shock
- growing dependence on alternative sources of income beyond local or national commissioners of their services. (Exworthy 2021)

ITB5 - **If** [efficiencies and innovation are enhanced in response to reduced public sector budgets] **then** [the health system will become less flexible and malleable] **because** [there is less 'slack' / room for further efficiencies during subsequent shocks].

ITB6 - **If** [efficiencies and innovation are enhanced in response to reduced public sector budgets] **then** [the health system will become more flexible and malleable, allowing a better response to a subsequent shock] **because** [there is an enhanced culture of innovation].

## Public dissatisfaction and political instability

Austerity leads to reduced public spending and job losses, producing unhappy public

Which produces political instability and change that can lead to

- Political extremism and populism
- Or new cooperation/coalition of opposing parties

If extremism

- Populist leadership less able to cope with governance challenges of shocks

If collaboration

- New politics allows unity and reform

Overly influenced by the middle-class bourgeoisie "the bourgeoisie do have an overweening influence upon the state. The bourgeoisie's ownership of the means of production provides the foundation for its influence because the state is obliged to rely on it to manage the supply of goods and services and the creation of wealth. That power is further reinforced by the infiltration of the bourgeoisie into the organs of state. The level of influence has accelerated rapidly over recent

decades. One of the consequences of this has been that healthcare systems have become rich pickings for the evermore confident bourgeoisie" (Porter, 2013)

Context = Fiscal governance at central (EU) level (Greer 2016 & Greer 2014), "E.U. leaders have created a large and invasive mechanism for monitoring and shaping member state policy in the name of the SGP that will fail on its own terms, neither being implemented nor producing the economic growth that is supposed to remedy the European Union's social problems. (Greer 2016)" (Also see Helderman, 2015)

More policy analysis of EU governance structures re. health policy (Fierlbeck, 2014)

ITB7 - **If** [public is unhappy due to job losses and reduced public spending] **then** [a new form of political leadership transpires, who are less able to cope with governance challenges associated with shock] **because** [political extremism and populism resulted from public outcry and attendant political instability].

ITB8 - **If** [public is unhappy due to job losses and reduced public spending] **then** [new politics based on cooperation/coalition of opposing parties emerges] **because** [unity and reform resulted from public outcry and attendant political instability].

## Empowerment

ITB9 - **If** [healthcare workers are given the opportunity; there is a change in the decision-making culture] then [new / better forms of healthcare delivery can be implemented in a timely and efficient way] because [people are motivated to succeed/improve/innovate; previous bureaucratic obstacles and challenges are (temporarily) removed]

## Short termism

Austerity leads to pressure on (public sector?) decision makers to cut costs and manage reduced budgets

This leads to:

- Short-termism in decision-making
- Reduced infrastructure and investment
- Reduced quality of care
- Reduced resources for education institutions for health professionals (Zabalegui, 2010)

Which then means that

- Some services/resources/experiences are forever lost to the system (irreversible?)
- Service provision is less able to deal with demands of an epidemic
- Best practice may not be utilised in favour of cheaper (outdated) procedures (e.g. surgical) (Karidis, 2011)

ITB10 - **If** [pressure is mounted on (public sector) decision makers to cut costs and manage reduced budgets] **then** [some services/resources are forever lost to the system; Service provision is less able

to deal with demands of an epidemic] **because** [of short-termism in decision-making; reduced infrastructure and investment; reduced quality of care; loss of skilled expertise/staff].

# Other considerations

## Communication

Poor insufficient data leading to bad policy decisions - (Gorantis 2014, and to lesser degree Repullo Labrador 2019)

## Population landscape

Co-morbidities. Populations with high levels of co-morbidities. Aging populations.

Solidarity theory as underlying theory of resilience (Saltman 2015)

## Systemic weaknesses

Lack of systematic financial structures, (e.g. under the counter payments); Public health insurance (Liaropoulos 2012)

Strong social protection mechanisms (both formal and informal) can mitigate some negative effects of recession on health. A great example of an informal mechanism for social protection in Cyprus and Greece is that volunteer Organizations have established Medical centers spread around the country, offering health services, free of charge to all those in need, whereas many volunteers are working intensively, raising funds and supporting directly chronic patients with palliative, psychological or supporting care, mainly at home (Theocharous 2014)

Privatisation of (elderly) care (Schwiter, 2018)

# Additional feedback from advisory group

- The impact of the economic shock on a previously disorganised system i.e. the state of the system before the shock as this might be an exacerbating factor? I imagine that a well organised, well-functioning system would weather an economic shock and subsequent austerity measures better.
- The political positioning/priorities in effect at the time of the economic shock (i.e. lack of real political will to address the shortcomings in the health system pre the shock; the lack of protection from the Troika decisions; and lack of prioritisation to protect the most vulnerable – leading to demoralisation of the leadership).
- Our history/political culture of effective implementation - good policies/poor implementation resulting in disaffection of the workforce where change initiatives are concerned.
- In general, how does the pre-existing eco-system of interventions (context) interact with austerity to produce varied outcomes that contribute to resilience (or not)?
    - What constitutes austerity will vary across countries. When austerity is introduced specifically what is eliminated and what is created? What is protected, what is sacrificed? and how might this make a difference to resilience?

- How do interactions of cuts within other parts of the open system impact the health system (e.g. social security)? Or existing/evolving systems of staff organisation – existence, strength and culture of unions? And does this matter for resilience?

- What is introduced alongside austerity e.g. managerialist (New Public Management style) approaches which may influence staff perceptions, roles, responsibilities… ratio of front-line workers to managers… perceived locus of expertise for system management…

IBT11 - **If** [managerialist (NPM style) approaches are introduced as a result of austerity] **then** [staff perceptions, roles, responsibilities will be altered/influenced] **because** [of changes in the ratio of frontline workers to managers].

- How does the existing risk culture interact e.g. Compliance focused, individualised risk, defensive risk cultures V's learning-centred cultures with shared responsibility? What are the perceived risks or goals that the wider system is responding to and how do these evolve through recession?
- Relative prioritisation of staff well-being versus other goals e.g. prioritisation of patient/service focused targets
- How do mechanisms for feedback in the system (existing and developed through the crisis) influence outcomes? Ability to detect impact of shock (austerity) on the system quickly and respond through adaptation especially considering unexpected emergent effects through interactions of policies across the open system
- Extent of central control V's devolvement – as an existing context and as a context that might evolve (turning it into a mechanism). One theory might be that the extent to which the system adapts to crisis depends on the extent to which it devolves responsibility and resources for action because in a crisis those at the sharp end are likely to be most immediately best informed on what is needed in their context and how to make it happen (could also do rival theory here – centralisation, strong, decisive, unified leadership…)
- How does the context of leadership interact with austerity?
    - E.g. heroic/designated versus distributed leadership
    - Transactional versus transformational leadership
- Existing context of resources and financing
- Prior experience of recession and responses to it (perhaps affecting expertise but also shaping public and staff responses to policies and what is perceived as permissible politically) – may create historical path dependence that continues into future shocks
- For whom? Are there differential effects within the health system for different parts or people and why? Considering all stakeholders here – staff, service-users, parts of the system. Is resilience across the whole system or bits of it/some people?
- How do societal or organisational values affect how austerity is implemented (thinking particularly here of Lipsky's Street level bureaucrats ideas) and does this matter for resilience e.g. because of ability to respond, or inaction, innovation or stagnation.. (again definitely rival theories here)
- Existing economic models e.g. neoliberal versus social democratic (on a scale)
- How do existing socio-technical systems e.g. IT recording systems and staff surveillance systems interact
- Existing social networks and loci of formal and informal power – sharing information, responses, solutions… or centre point for obstruction, facilitation, staff and other stakeholder discontent to galvanise…or ….
