# Supplementary material for: How International Health System Austerity Responses to the 2008 Financial Crisis Impacted Health System and Workforce Resilience – A Realist Review
Source: Int J Health Policy Manag. 2022 Dec 24;12:7420. doi: 10.34172/ijhpm.2022.7420 (PMC10125082; doi:10.34172/ijhpm.2022.7420)
Supplement: Supplementary file 2 — Preliminary CMOC Summaries. [file ijhpm-12-7420-s002.pdf]

Article title: How International Health System Austerity Responses to the 2008 Financial Crisis Impacted Health System and Workforce Resilience – A Realist Review

Journal name: International Journal of Health Policy and Management (IJHPM)

Authors' information: Padraic Fleming[1]*, Louise Caffrey[2], Sara Van Belle[3], Sarah Barry[1], Sara Burke[1], Jacki Conway[4], Rikke Siersbaek[1], David Mockler[5], Steve Thomas[1]

[1]Centre for Health Policy and Management, Trinity College Dublin, The University of Dublin, Dublin 2, Ireland.

[2]School of Social Work and Social Policy, Trinity College Dublin, The University of Dublin, College Green, Dublin 2, Ireland.

[3]FWO, Institute of Tropical Medicine, Antwerp, Belgium.

[4]Everlake, 5 Marine Terrace, Dun Laoghaire, Dublin, Ireland.

[5]Library Reader Services, Trinity College Dublin, The University of Dublin, St James Hospital, Dublin 8, Ireland.

(*Corresponding author: Padraic.fleming@tcd.ie)

Supplementary file 2. Preliminary CMOC Summaries

Contents

Appendix 2 – Preliminary CMOC summaries 1
Summary of Context Mechanism Outcome Configurations (CMOCs) developed 2
CMOC 1 - Value shift: 2
CMOC 2 - Power game: 2
CMOC 3 - Fighting the system (access): 2
CMOC 4 - Health behaviour change: 3
More detailed CMOCs 3
CMOC 1 – Value shift 3
Notes on CMOC1 3
Extracts of text to support CMOC 1 3
Value shift 3
Deminished view of profession 5
CMOC2 – Power game 8
Notes on CMOC2 9
Extracts of text to support CMOC 2 9
Powerless 9
Lack of autonomy 9
Control of decision making 11
Resistance to change 11

CMOC3 – Fighting the system (Access) 12
Note on CMOC 3 12
Extracts of text to support CMOC 3 12
Circumvent policy to deliver care 12
CMOC 4 – Health Behaviour Change 15
Notes on CMOC4 15
Extracts of text to support CMOC 4 16
Health seeking behaviour 16
Medication mismanagement 16
Health illiteracy 18
Under-developed themes/theories 19
Patient outcomes 19
Extracts from literature organised by theme 20
Blame game 20
Distrust 20
Ethical decisions 22
Financial accountability 22
Governance 23
Lack of transparency 25
Communication 26
How decisions perceived / framed 27
Informed decision making 28
Political ramifications 29
Performance monitoring 31
Opportunity to reform 32

Summary of Context Mechanism Outcome Configurations (CMOCs) developed

CMOC 1 - Value shift: In context of restrictive fiscal policies (staffing, consumables, treatment options), a value shift is evident for health professionals, from patient-focused to economic, with long-term consequences resulting in a diminished view of the profession, apathetic and burnt-out.

CMOC 2 - Power game: When health professionals feel a loss of autonomy and decision-making power, a sense of powerlessness and detachment led to resistance to change and a conflict between front line workers and policy decision makers / management.

CMOC 3 - Fighting the system (access): A sense of professional / moral duty or ethical decision making, solidarity with patients or fellow health professionals led to health professionals

circumventing policy to deliver care (legal, informal referrals, treat regardless of ability to pay), but ultimately lead to strain on frontline workers, increases ER use but more stable health outcomes that originally predicted.

CMOC 4 - Health behaviour change: With the introduction/increase in OOPs, health-seeking behaviour change, compounded by health illiteracy, led to reduction in primary care usage, increased emergency care, medication mismanagement, delayed treatment

More detailed CMOCs

CMOC 1 – Value shift

In the context of staff shortages (Workforce x 8, Access studies x 3, Decision making x 2, impact x 3, service delivery x 2) and pressure from decision makers, management duties were imposed on senior clinical staff (while junior staff were substituting for senior positions). Health professionals found themselves having to make decisions they never had to make before (driven by restrictive policies aiming to achieve efficiencies in terms of Human resources, consumables and materials, or transportation AND/OR a need to prioritise/ration treatments/medications due to policy-driven efficiencies or populations inability to pay). Combined with worsening work conditions (pressure, decreasing pay, longer working hours, stress etc., challenging work environment).

Shift from patient-focused values to economic values (mechanism) (Barradas, 2017; Heras-Mosteiro, 2016 (minority of respondents); Tucker, 2020; Pellinan, 2018; Thomas, 2013 (loosely))

Overworked, stressed, burnt-out staff, who had a diminished view of their own profession (viewing it as a conveyor belt and themselves as 'workers' rather than 'professionals' and began to become apathetic about the healthcare delivery / the wider health system.

Notes on CMOC1

(inappropriate task shifting – Training (not trained to be businesspeople))

Clinicians vs nurses – is there a difference in value sets?

Failure of system to show adaptive resilience – physiotherapist bed blocker study (inappropriate high-cost setting)

Shift away from patient-focused values looks like: shorter consultation times (conveyor belt); lack of doctor-patient trust (no time to build trust); breakdown in communication (Barradas, 2017)

This shift is also true for policymakers, although perhaps they were less patient-focused in the first place. Regardless, policy decisions have downstream impact on health workers in terms of restrictive policies, which also feed into the same CMOC.

Extracts of text to support CMOC 1

Value shift

Barradas, 2017

the effects of economic scarcity and mandatory cost control often emerged as important issues, either as causes for change in medical practice or as a factor which deteriorated the trust

relationship established between health professionals and patients (a decisive dimension of the welfare state)

There is one thing that worries me very much, which is the loss of confidence in the doctor, in the system. I can be an excellent technician… But if I don't convey trust it's complicated… because the person [thinks], 'what does it matter if he is an excellent technician, if he doesn't talk to me'… Obviously there is a political aspect to disinvestment in the SNS. But, I think that the doctors are widely blamed… I had a director of services who, in a discussion on how long a pre-surgery consult should take, said that five minutes was enough. If a person has consultations every five minutes, that says it all! And sick people, they can't even come in! Not even sit down… I'm a fabulous technician. My consultations take a minute. Now the patient, what opinion of us will he take away?… This massification imposed by the system, by the politicians, the managers, the service directors… The pressure to always produce more… It is cheap, it's free, isn't it? And people want to consume, and this leads to that person who had the appointment… But who leaves frustrated (Physician 2).

The change in the patterns of medical assistance in recent decades has been associated with the weakening of a humanistic approach to patients in the name of cost-efficiency. The corporate interests of healthcare companies have actively promoted this change. In the final analysis, this can cause irreversible damage to the trust relationship the Portuguese population has in its public health system

Pellinan, 2018

Clinical professionals reported an increase in performance measurement, mandatory statistical reporting, and financial accountability at the cost of good patient care. Examples include a new access control system with working time metrics, increased reporting responsibility to outside organizations, administrative responsibilities, demands for cost-cutting, and an increased emphasis on efficiency measurement:

As a doctor, I find it hard to accept that reporting our work to outside organizations is more important than finding the right treatment for a patient. It seems that nowadays, everything is more important than the patient (Deputy chief physician).

Modern patient work involves input into different clinical databases. This, in turn, has enabled the city management to push financial accountability in the form of various performance measures to the clinical professionals irrespective of the budgetary control of individual clinics.

Balfe, 2013

A number of young adults felt that the Irish healthcare system was increasingly concerned about providing them with the cheapest care rather than the best care. One young woman, for example, noted that auditors in the health system questioned her pharmacist to find out if she could be provided with fewer glucose test strips. A number of young adults also felt that pharmaceutical companies were beginning to fund functions that had previously been standard aspects of public sector care, such as glucometers and diabetes training programmes.

Tucker, 2020

Louise spoke about the increased requirement for senior therapists (referred to as band sixes, sevens, and eights) to spend time developing business cases.

Louise had experience of obtaining nonclinical skills to assist her in service development:

What we are forced to do, certainly as band seven physios is to be entrepreneurs and to be business minded; I've taken business courses and financial management courses through the NHS, I can look at tariffs and I can break down, look at bed days, and look at things from a business point of view not just a patient point of view; and from that we have set up new services, and run those from a financial efficiencies point of view (emphasis added as shown in bolding).

Louise suggested that the time spent engaging with these business cases created inefficiency:

We are wasting money and spending so much time wasting, not [assistants], it's band sevens and eights [seniors], costing the commissioners and the NHS a fortune, doing very little for a good chunk of our time in terms of productivity and patient contact because we are now chasing our tail, catching up with the latest change in politics.

Kerasidou, 2019 – Empathy
According to Doctor 7 below, those in positions of power have a specific view of how medicine and care should be practiced, and they are using austerity and efficiency-driven arguments in order to alter the nature and ethos of healthcare. In the process of trying to change medicine from a "profession" into a "job" what is lost is not the clinical aspects of the role but the caring aspect of these professions:
To actually have people to properly look after people costs a lot more and they (the government) are not prepared to pay for it. … The quality of caring, you know, I've seen that drop. (Doctor, 7)

More loosely related

Heras-Mosteiro, 2016
The second, named the "economic-based argument," was supported by a minority. The economics-based argument objects to the law on the basis that excluding the migrant population from health care access will actually increase costs related to emergency services, due to the worsening of chronic conditions and infectious diseases.

Thomas, 2013

The feeling within the healthcare sector is that health system transformation has been playing second-fiddle to more immediate goals such as expenditure reduction and technical efficiency savings.

Deminished view of profession

Pellinen, 2018

The mayor's means are mostly limited to rhetorical speeches and budgetary control (resulting in even more emphasis on financial accountability) in a situation where most of the city's clinics are already understaffed, and the work in clinics is described in the local newspaper as strenuous "salt-mine work":
The vice manager of the doctor's union commented on the situation of the FHC clinics as being like working in a salt-mine […] especially young doctors do not want to work there. The ratio of pay to volume of work is just so bad in the FHC […] In the FHC area, understaffing and difficulties in attracting doctors for vacant positions have been a constant issue (Local newspaper article January 20, 2014).

Humphries, 2019

'the deteriorating quality of the job ...we were having to take responsibility for . . . juniors that weren't up to scratch and for doing extra work for colleagues' (Respondent 31).

Kerasidou, 2016

Yet, many frontline healthcare workers feel strongly that the austerity-driven reforms not only cause them financial hardships, but undermine them as professionals. The following doctor argues that it is not only patients who are the casualties of these reforms but the respect and value shown to doctors has suffered too. He explains how the low salaries offered to new doctors are interpreted as an affront to their profession:
When you don't hire new doctors on the islands or in other remote areas, and there are so many residents there without a doctor and you do nothing about it. When you send someone with €1000 salary (on a 1-year fixed-term contract), someone who is 35–40 years old, who probably has a family, how can you send this doctor for 1 year to this island? You could at least have them stay there for 5 years, isn't that right? At least you could give them some time to get there, to find a house, to settle in that place. So it is as if they are mocking us. They are mocking both the doctors and the patients (I8).
For such reasons, many frontline healthcare professionals interviewed regard their allegiances as being with their patients, as both groups are perceived to be under attack by austerity measures.

When someone is in the operation room and you pay them €4 per hour to save a life, it's like you insult their work, there is no question about it. And I should better leave it at that. You just insult what they do (I8).

This idea of being insulted and affronted is a theme
repeated by those interviewed, on the topic of salary reductions. In general, the huge reduction in earnings is deemed to be an affront to the whole profession, which undermines motivations and demeans the importance to their work. In the following quote, we learn from a healthcare profession why doctors are deserving of respect:
… the political system regards the doctor as just a service provider, the same as any other public servant. Yet, from the moment that they regard them as that, they seize to respect them. So they will assign to them a thousand jobs, without paying them properly, and even worse than that, without showing any respect…a doctor deserves respect […] because they put their own health in danger for the sake of their patients'

Now regarding the A&E, when you see them (the patients) stacked, literally, three times the number we can handle … a dignified examination needs some time, you need to check their health history, to do the clinical examination… I have the feeling that we work in a factory. Each patient has 5 min, when one goes another comes in (I6) [Emphasis added].

Kerasidou, 2019 – Austerity

However what happens is . . . we become, A&E stands for anything and everything, not accident and emergency I think, its anything and everything, just send it to A&E. GPs who run out of things they can do with patients, they send them to A&E, Police if there are patients acting strangely they send them to A&E; or I am feeling a bit worried about my cough, I have to go to A&E. . . . That is a big problem for A&Es at the minute." (Nurse,12)

That's something we don't do in A&E, we don't bring people back. . . when you do something out of the norm because you are trying to improve flow, it does feel uncomfortable at times. . . it feels uncomfortable to make decisions that are weighted on flow rather than clinical care.

There was a consensus among those interviewed that targets did not necessarily reflect
overall quality of care, and, at times, obscured patient needs. As one nurse mentioned, the focus on quick turnaround times and rapid referrals made him "feel a bit like working on a conveyor belt"

(Nurse, 15), while another participant described it as "turning a profession into a mere job" (Doctor, 5) as a ways of explaining the depersonalising effects of solely focusing on measurable targets rather than the quality of care provided.

. . .part of being a doctor or nurse in an emergency department is to have that face that says, "Hello I'm approachable, I'm here to help"; which we all wear very well. If we couldn't wear that face every day, the emergency department is not the right environment for us to be working in. [. . .] People become a little bit more blunt and even snappy with each other, not with patients, because again, you know, I think that none of us would want to be that way with patients, but with our own friends and colleagues we can let our guard down a little bit, we can take away the professional face. And I think that is apparent, I see it with my nursing staff, I see it in my registrars and I see it increasingly within the consultant body that I work in. (Doctor, 8) [Emphasis added]

Kerasidou, 2019 – Empathy

The pressure to meet targets is leading departments to adopt models of organisation that resemble Fordist lines of production, rather than places of care. For example, interviewees explained that one nurse will be responsible for cannulating patients and taking bloods, and another will be administering drugs or checking electrocardiograms (ECGs). Even though this division of labour may help with the flow of patients through A&E, it also results in healthcare professionals becoming "de-skilled" (Nurse 12) as they become more specialised but less versatile.

Yet for others, the operationalisation of medicine in the name of efficiency has
more profound effects on the way they perceive themselves as well as the patients they treat, as Doctor 2 commented:
it is difficult to engage and empathise with patients. I think it kind of depersonalises them and you. It kind of makes you feel a bit less human [...] as if you are a robot or a computer. (Doctor, 2)

Ortega-Galan, 2020
"sometimes, I feel or think and I believe that charity has returned, social services were born to put an end to that kind of help, which I find humiliating, it's the most humiliating thing that's happening that we thought had disappeared, charity doesn't go hand in hand with social justice, people are entitled to basic benefits" (IDIs, p3)

"There are days when I think . . . the system is a mess! Because in the end nobody takes us into account, we write a social report and unless the people in front of you are colleagues who know what it's really like, other professionals don't understand that you have described the situation because there aren't any resources" (IDIs, p5)

Russo, 2016
GFM specialists complained mostly about the administrative controls brought in as an austerity measure, such as biometric checks and the obligation to clock-in and clock out of the service. Hospital doctors expressed their contempt about the obligation for mid-level professionals (médicos consultores) to do their share of emergency shifts, which formerly were an exclusive remit for entry-level doctors (médicos auxiliares). Both measures were widely seen as a reduction of physician professional autonomy, of the privileges linked to the physician status, and ultimately as a degradation of the profession. "Look, I have been acting as a senior specialist in this hospital for over 10 years; I work 12 hours a day; I need my sleep – I cannot be doing night shifts anymore alongside 20-year-old apprentices…". Senior hospital specialist.

Jane suggested these difficulties had put physiotherapists at risk of burnout or questioning the

occupation entirely:
I've seen a lot of people questioning or leaving the organization, purely because its feels like you're knocking your head against a brick wall because you're not able to get on with what you need to get on with, extra pressures being put on you . . . and I think that has driven people to other lines of work completely.

The conflict between fulfilling professional responsibilities within organizational constraints, resulted in feelings of powerlessness and subjection. The reality of this was illustrated through Louise's experience. She demonstrates feelings of powerlessness and fatigue while undertaking extensive work during the decommissioning of her service. Louise's feelings toward this process are evident in the following excerpt:
That's what made me so cross about all these changes, is that we offered ourselves, I offered my business case, I presented, I whored myself around like some cheapskate, I was banging my drum constantly, I had no shame at all, I didn't care who I presented in front of, I presented to everybody, and still . . . . they made all these decisions (emphasis added as shown in bolding).

Fana, 2021

Some of the staff refused (such as mortuary staff acting as porters), if they considered the task to below their pay grade. Others responded by abusing their leave; a senior manager remarked on the increase in absenteeism and sick leave. One staff member admitted: 'I just go to the doctor and book myself off so that I can rest' (FGD: General Assistants Hosp. A

CMOC2 – Power game

Tensions between policy-makers/organisational structure/management and health care professionals on the face of it often relate to the tension between economic values and patient-focused values, however there is evidence to suggest that health professionals believe they are losing their sense of autonomy, particularly around control of decision making (financial accountability e.g. approval of diagnostics; permission to use a new medication; introduction of compulsory electronic procedures for ordering medication; time management (clocking in); ability to book training opportunities etc.), senior staff required to do emergency cover (and other aspects of the role changing), substitution of roles).

Sense of powerlessness, feelings of detachment, desire to stay in control (mechanism), breakdown in communication

Resistance to change. Conflict between health professionals and management, administration, policy makers (power-game). Circumvent policy to deliver care. Act in a way that is non-collaborative (with management) while building a deeper sense of solidarity among health professionals.

Notes on CMOC2

This latter point is important because it reinforces the very thing that is creating a tension between patient-focused values and economic values. Health professionals through their desire to stay in control are involved in more management / policy-making processes than actually delivering care.

Add in here some of the things that built resilience – teamwork, staff-substitution, solidarity,

Extracts of text to support CMOC 2

Powerless

Papadakaki, 2017

The healthcare providers felt powerless about supporting migrant healthcare with such low capacity in the system. They felt that they were ineffective with regard to their ability to bring changes to the system to improve migrant healthcare

Kerasidou, 2019 Austerity

The harder decisions are what you do when the system is crashed around you. So often I will be down there, and maybe its unsafe, you know, there may be people dying, literally dying in the corridor and I used to worry about that too, but I don't now because what I do is email the duty manager and say, "you need to know it's dangerous, you need to sort it out"" (Doctor, 7) [Emphasis added]+
Feelings of detachment and of powerlessness in the face of the conditions created by austerity policies were common among those interviewed.

Papadakaki, 2017
The healthcare providers felt powerless about supporting migrant healthcare with such low capacity in the system. They felt that they were ineffective with regard to their ability to bring changes to the system to improve migrant healthcare. They thought themselves as being the final recipients of political decisions without any scope for active participation in these decision-making processes. They referred to continuous updates to Greek laws and policies regarding migrants' healthcare and reported a huge difficulty in daily scheduling or in making plans in a healthcare system that keeps changing day-by-day.

Lack of autonomy

Baradas, 2017

The increase in clinical criteria for approval of diagnostic methods is becoming a way of deterring and discouraging medical staff from requesting them, a trend which is likely to continue:
Ah, yes! It is much more difficult now, of course, to schedule a CAT scan, a mammogram, a PET scan, or to ask for a new medication. You have to justify much more and there is the possibility it will not be approved. [Justification is based] on the criteria of patient need. It is part of the protocols that already exist. But even with the need documented at this time, any characteristic that prevents this approval is taken into account. And before it was easier… for example, to get an 80-year-old patient to have the right to treatment, than it is now – according to age, for example, or to general condition. Whereas before it was a selection criterion (Physician 1). Yes, small things where you can perceive resistance… With PET scans this happens a bit, although we have never been denied, but we have to ask… There is this bureaucracy that in some form is meant to limit requests, but things keep functioning (Physician

2).

Hollingworth, 2015

Clinicians in PCT2 expressed similar views with regard to the threshold policies, but the intermediary services and prior approval system largely removed clinicians' responsibility of assessing patients against these criteria. By the time a patient had progressed to seeing a consultant, their eligibility for surgery will have been determined by the PCT:
But now they [patients] don't get to me, because someone else is making some sort of decision that they need steroid injections or they don't need carpal tunnel surgery or they do. Interview, group B, PCT2, Clin7

The above perspective was similar to that of a clinician from PCT1:
I mean, I feel I'm being interfered and manoeuvred, but actually when a patient is sitting in front of me, if I need to operate on that patient, a lack of funding or anything like that has not stopped me from doing it – I've always been able to do that.
Interview, group A, PCT1, Clin4

Correia, 2015

The monitoring of public hospitals' performance and of drugs prescription through compulsory electronic procedures intended to limit doctor's autonomy.

Kerasidou, 2019 Empathy

They also lose their professional autonomy, namely the opportunity to exercise clinical judgement regarding the care of patients. According to Doctor 7 below, those in positions of power have a specific view of how medicine and care should be practiced, and they are using austerity and efficiency-driven arguments in order to alter the nature and ethos of healthcare.

Russo, 2016
GFM specialists complained mostly about the administrative controls brought in as an austerity measure, such as biometric checks and the obligation to clock-in and clock out of the service. Hospital doctors expressed their contempt about the obligation for mid-level professionals (médicos consultores) to do their share of emergency shifts, which formerly were an exclusive remit for entry-level doctors (médicos auxiliares). Both measures were widely seen as a reduction of physician professional autonomy, of the privileges linked to the physician status, and ultimately as a degradation of the profession. "Look, I have been acting as a senior specialist in this hospital for over 10 years; I work 12 hours a day; I need my sleep – I cannot be doing night shifts anymore alongside 20-year-old apprentices…". Senior hospital specialist.

Tucker, 2020
. . there were times previously . . . describe it as the time of milk and honey, you know, if you wanted to go and do some training and implement that; whereas now you really can't make those decisions, there really aren't those value added experiences for staff and for patients because it's obviously more patient orientated, but it's becoming more lean.

These excerpts demonstrate circumstances where physiotherapists' autonomy was challenged by wider constraints from the organization. Such constraints dictated their ability and capacity to perform and deliver their services, suggesting tension between professional autonomy and organizational structure. Consequently, austerity appears to have changed the participants' perception of their autonomy. Their ability to make decisions freely was curtailed by the requirement to meet targets and create cost efficiency savings.

Control of decision making

Hollingworth, 2015

There was one exception to this, where one clinician acknowledged that he had, on occasion, had to explain to patients that their surgery would need to wait until they had tried conservative therapy. This clinician reported being very open about the lack of control he had over the situation, diverting responsibility to the PCT:

Yes I have told them that um the reason why they are having this treatment, and not an operation which they possibly expected to get, is that they don't fulfil the criteria set up by the PCT. Interview, group A, PCT1, Clin3

Pellinan, 2018

The problem is, the power of the profession is really high, doctors think that because we've always done like this, there is no reason to change. They are always trying to resist different development ideas (Financial manager).

The autonomy of the clinical profession is so strong that it does not need to take on development ideas from outside of the profession; the only time that outsiders are included in the talks is when they involve determining resources for the operations. Even in these situations, clinical professionals tend to strive for control over the discussion and terms:

Well, at least in my personal opinion, whenever I have tried to suggest some course of action or development idea, they (doctors) have been very cold, to the point of throwing me out of their meetings. For example, I tried to offer a development idea about co-ordination of closing times between clinics but nobody listened (Development officer).

Resistance to change

Pellinen, 2018

I think it was about one and a half years ago that the mayor was talking to the clinical management team and said that the situation is dire, the situation needs to be improved, both in terms of mutual relationships and of budget control. He also said that if someone thinks that they're not accountable, or they don't want to be, they don't have to be here (in the organization). And I was there, too. And I looked at the expression on their faces [clinical professionals] and I already knew they weren't going to change a thing (Deputy chief mayor).

The autonomy of the clinical profession is so strong that it does not need to take on development ideas from outside of the profession; the only time that outsiders are included in the talks is when they involve determining resources for the operations. Even in these situations, clinical professionals tend to strive for control over the discussion and terms:

Well, at least in my personal opinion, whenever I have tried to suggest some course of action or development idea, they (doctors) have been very cold, to the point of throwing me out of their meetings. For example, I tried to offer a development idea about co-ordination of closing times between clinics but nobody listened (Development officer).

CMOC3 – Fighting the system (Access)

Resilience to a diminishing view of profession seems to lie in health professionals who circumvent policy to delivery care. This takes many forms, including legal challenges to restrictive policies (with legal outcomes leading to regional differences in policy implementation – proximal outcome); informal referrals to specialists (friends); referral to ER; issuing medical reports to support applications for health coverage; or simply ignoring the rules to treat migrant populations or others (elderly, disability), even though they cannot pay their bill (hospital managements problem to figure out).

Heightened sense of duty, moral obligation, ethical decision making, resistance to change, solidarity, awareness of impact on population health (if for example, infectious disease within migrant populations is not controlled/treated). - Mechanisms

Leads to strain on health professionals (fighting the system), increased ER use, stable health outcomes contrary to predictions. This can also feed into the diminishing view of profession.

Furthermore, this impacts quality of care provided, staff retention,

Note on CMOC 3

I think the evidence around the shift away from a 'blame game' in terms of reporting shortcomings is a positive aspect that seems to have emerged post-recession. Not sure how much evidence to support that, but certainly cropped up a few times

Extracts of text to support CMOC 3

Circumvent policy to deliver care

Cimas, 2016

Table 2 includes the 29 documents found, which correspond to the 15 Regions that took legal, legislative actions or produced internal directive documents oriented to counteract, mitigate or module the healthcare coverage of irregular migrants as defined by the RDL 16/2012
Legal actions Six regions (Andalusia, Basque Country, Canary Islands, Catalonia, Navarra and Asturias) showed an up-front legal opposition against RDL 16/2012, filing appeals against it to the Constitutional Court. The appeals of Navarre and Canary Islands were based on violation of the constitutional right to health. In the ones of Andalusia and Catalonia, this main argument for the opposition to RDL 16/2012 was the interference of the Central Government in regional competences.

3.2. Legislative actions Twelve Regional Health Authorities (70.58%) have passed specific legislation (1 regional law, 3 orders, 8 instructions) in order to regulate entitlement to healthcare for undocumented migrants and/or to modulate the consequences of the application of RDL 16/2012. Among these, the most relevant is the Navarre Regional Foral Law 8/2013 which, using this Region higher level of autonomy, re-established their pre RDL 16/2012 status regarding entitlement to healthcare.

Administrative actions
Ten Regions have formally stablished alternative administrative pathways of access to healthcare services for low-income undocumented migrants, mostly including both primary and secondary care. Through these regulations, Regions standardized healthcare access for this group of population for limited period, most often of one year.

Gogishvili, 2021
As a response to the 2012 health reform, some autonomous communities (including Valencia), due the decentralized nature of the Spanish health system, developed various regional regulations and

instructions to still provide access to free healthcare to immigrants

Porthe, 2016
According to medical professionals, the restriction of coverage has led to the use of various unofficial and official strategies to facilitate the care of severe cases (e.g. heart disease) or of those that require immediate monitoring in secondary care, despite not having the IHC or the necessary coverage. The former include: informal contact with specialists to refer patients, referrals to hospital emergencies or, to a lesser extent, including the patient in their assigned primary care population. The latter include: issuing medical reports in support of applications for the full coverage IHC or, in the case of some providers, creating a specific service to authorize access to secondary care in urgent cases

"I[the girl] couldn't breathe (. . .) they didn't want to give her an appointment because she didn't have a health card (. . .) [another nurse intervened] you can't refuse the girl medical treatment

"When that law was passed [RDL 16/2012] (. . .) in the outpatient centre we had a meeting and said that we'd attend to everyone"

Hollingworth, 2015
Looking at threshold criteria alone, clinicians across both regions felt these had little impact on their clinical practice. For clinicians in PCT1, this was largely because of their involvement in writing the threshold criteria. Those consulted during policy formation explained how they had included a 'catch all' clause that preserved their ability to act in accordance with their own clinical opinion:
We haven't rigidly defined 'severe symptoms'. So there's a clause that says you can operate on them if they've got severe symptoms . . . it's the ones I want to operate on.
Interview, group A, PCT1, Clin2

Careful framing of patients' symptoms also allowed clinicians to demonstrate eligibility if they felt the patient warranted surgery. Across the board, clinicians from PCT1 demonstrated that they were very much in control when it came to making decisions about surgery:
Well um you don't want to um – you can't invent symptoms, you can't invent something that isn't there, and one shouldn't do it, because that's a very slippery slope if you do that. But of course, you can guide the patient to give the answers that enable you to put them on a waiting list, giving them questions that will lead them to – um – perhaps make their symptoms more intrusive than they possibly are, if you feel strongly.
Interview, group A, PCT1, Clin3

One clinician described this process, maintaining that they had never allowed threshold criteria to prevent them from operating on a patient they felt truly required surgery:
It's like everything else: you find ways around the system. [. . .] I have a patient in front of me who needs treatment. And if I think the best thing for them is carpal tunnel surgery then quite clearly, on paper, they will meet the criteria. So the whole thing is a nonsense.
Interview, group B, PCT2, Clin9

Cervero-Liceras, 2015

Around half the interviewees had doubts about whether the Royal Decree was being implemented, either because managers would not impose it or because health care professionals were finding ways to disobey it and provide the same care as before. However, there was also a sense that this would not last very long:
104 F. Cervero-Liceras et al. / Health Policy 119 (2015) 100–106
P-There is an Argentinian family that has all the correct documents to receive healthcare. But one of the sons is over 18 and he doesn't have them. He is a psychotic with a schizophrenic disorder and is

impaired, so he has no healthcare coverage.
I-And how did you proceed? P-Looking for subterfuges in a thousand ways, and there is still around two years for a legal process, an administrative process, to obtain the coverage. Meanwhile it is discussed, it is enquired, it's done, but for now it is given (the care) [. . .] but I know that at any point it will be removed (the right for coverage) (I13).

Heras-Mosteiro, 2016

Yes, there have been [immigrants who were excluded from the right to health care] specific patients that would sometimes tell you. They say, "I don't have the card anymore." I12 466–468
All participating health professionals responded to the new restrictions by continuing to provide care to the excluded population. They mostly argued in favor of providing health care to those who need it and against the exclusion of anyone that needs care.

Oh, it's a no-brainer for me, I see everyone, that's a given, and we try to help everybody here ... I believe, myself and all my colleagues, that no one is going to be kept outside those doors and no one will remain unattended.

Exercising conscientious objection to the new regulation and continuing to serve this population in the same manner as before did not present an ethical dilemma to most health professionals.
I have an ethical dilemma for about five seconds since I see the patient whether it is an emergency or not. Let's see, how can they tell me to refuse seeing a patient unless it's an emergency when I saw him the week before for whatever reason? That doesn't make sense to me. So, for me, it's not an ethical dilemma.

I believe that we should serve immigrants because they bring a series of pathologies, especially infectious and contagious diseases, which must be treated, otherwise they will spread to other sectors of the population [.. .]. I think they made a mistake regarding immigrants' access to care. I think they will reconsider that one, because they've seen that it does not save them that much money after all.

Kerasidou, 2016

In what follows a doctor, volunteering in a Community Clinic, which relies on donations to provide healthcare and treatment to those unable to afford it, explains how austerity measures has shaped access to medicines:
…the problem is that they cannot afford to buy their medication… In the end of the day, someone will always find a doctor to examine them; they will either go to a public hospital and pay these €5, which is not much – of course nowadays there are people who cannot even afford to pay these €5- or they can go to a regional clinic which is free, or there are many doctors, good people who see many patients for free, I personally know many doctors who do that. But no one will ever give them their drugs for free (I13) [Emphasis added].

For frontline healthcare professionals, managing requests from the uninsured raises tensions between following the rules and fulfilling their duties as doctors and nurses. In these instances, the staff interviewed mention that they manage such tensions and dilemmas by interpreting the new rules loosely, to make them comply with their sense of professional duty.

Our guidelines are that we have to medically treat them (the uninsured) as long as it is an emergency, and this is certainly what happens. As for the chronic conditions, things are bit muddled. For these situations the guidelines are vague and their implementation tends to be more towards trying to cover them [the patient] medically, although this means that we could face some sort of repercussions, but admittedly, this does not happen. […] But there are people who suffer from kidney

failure, and need dialysis... for this person to be considered an emergency case they must have reached a pre-death stage [...] On the other hand providing them with a dialysis three times every week would be considered chronic treatment. Well, we label the dialysis as an emergency treatment and we go for it. And nobody stops us. And of course we do the right thing [Emphasis added]

Has there ever been a case when you had to deny treatment to someone, after dealing with any urgent issues of course?'
DR: No, never. I: Never in all the years you have been working? DR: No, you cannot do this. I: Why can't you do it? DR: Well, it's an issue of our professional code of ethics, how can you do this? I: And what about the management? What if the management told you that there is no money to cover for this patient? DR: Then it is for the management to find a solution, and not me (I8).

CMOC 4 – Health Behaviour Change

In the context of restrictive policies, often driven by MOUs (health expenditure cuts aimed at making efficiencies), vulnerable populations (elderly, migrants, unemployed, people with disability, rural populations) were hardest hit by the introduction/increase in out of pocket payments / co-payments for care.

Mechanisms – Health-seeking behaviour change driven to save money; to address/prioritise the worst health condition; compounded by health illiteracy

Led to reduction in those seeking preventative primary care, reduced access to healthcare, medication mismanagement, shift from private to public system, increased emergency room use (also a mechanism for diminished view of profession), poor quality healthcare, delayed treatment with worsening prognosis; ignoring invisible conditions (e.g. mental health)

Notes on CMOC4

MOU, restrictive policies aimed at efficiencies – cuts, increased waiting lists

Who? – Migrants (poor employment rights further delaying health seeking behaviour, various residency, and other social health-insurance requirements), Older people, rural populations, Middle class, Mental health problems

People living in poverty, unemployed, ineligibility for medical/health card.

Access did not appear to be a problem for wealthier people who could pay for private health care

Positive: seeking generic drug alternatives; shift from private to public system; improved reporting of medication errors

Health professionals faced with the decision to ration care or prioritise treatments ...

People experience health illiteracy because they are migrants or simply populations of people who don't understand the complexity of accessing health care. Through guidance and support from NGOs or friend who previously navigated system, sometimes they were able to navigate the health care system and get access at the appropriate entry point. In many cases this was simply emergency care.

Guidance and support took the form of information provision, directly liaising with health professionals, completing administrative task (completing forms, getting referenced)

Extracts of text to support CMOC 4

Health seeking behaviour

Doetsch, 2017

The health budget cuts under the MoU were seen by respondents to alter elderly patients' health care seeking behaviour. Elderly were identified to attend less and avoid regular check-ups at the primary care service facilities as a result of the cuts on free of charge non-emergency patient transportations (ID10; ID11).
"And they used to have [ehm] free [ehm] ambulances from fire man but the financials of transportations was cut because of the troika. And now they have more difficulties in going to primary care or going to hospitals." Public health expert

Porthe, 2016
Users report that these waits force them to give up seeking treatment because absences from work or delays put their job at risk, and also that they lead to attempts at self-medication (Table 4e).

Medication mismanagement

Carney, 2017
noncompliance with medications as barriers to effective care barriers to access to health care services especially for elderly with a middle income pension and chronically ill patients (ID5; ID9). Respondents negatively evaluated the exemptions from co-payments for chronically ill patients as these exemptions were limited to medications which are directly related to the chronic condition, even though chronic conditions usually require the intake of several medications due to the co-occurring diseases (multiple morbidities) (ID3;ID11).

Still a significant share of pharmaceuticals was reported to be paid by the elderly patients through OOP. OOP was stated to restrict the affordability in the purchase of pharmaceuticals and to influence a fundamental problem for elderly with chronic diseases: polymedication, the usage of four or more medications by a patient (ID2;ID5;ID9;ID10,ID13).

"Mainly for those with chronic diseases, that have to follow daily specific medication, sometimes they even had to choose which is the most important medication, because they can't afford to buy both, mainly diabetes, cardiovascular diseases. [...] There are problems with medication, they go to this specific doctor and to the other one and all prescribe different medications and the interaction between medications is really bad." [Translated quote] Municipality authority (ID3)
OOP and financial constraints forced elderly to decide which drugs to purchase after the prescription of the General Practitioner (GP) (ID3;ID5). This was observed to ultimately result in a lack in quality of healthcare through ineffective treatment, severe interactions of medications, lack of monitoring and the increased risk for coronary artery diseases (ID2; ID6).

Gogishivili, 2021

Of course, I went because I had been without medication for 2 weeks.

Alverez-Galvez, 2019

Firstly, a deterioration in the health of the elderly has been observed, which is related, on the one

hand, to poor consumption of medicine (e.g. self-medication)

Participants highlighted an increased difficulty in accessing certain medications that were subsidised before the crisis, and which currently are either no longer subsidised or require co-payment. This especially affected some highly recommended medications or even ones prescribed by doctors, such as some vaccines for children. This also affected long-term medical treatments. As a result, many users end up abandoning them, due to the impossibility or permanent difficulties paying for such medical treatments.

Cervero-Liceras, 2015
The majority of health care professionals, who were against the introduction of copayments, shared stories of patients being unable to pay for medication. For example a surgeon recalled the story of a patient not taking his antirejection medication because he could not afford to pay for it, and later dying of a related complication: R-So, when a patient comes and tells you that he can't afford the drugs, you pick the drugs he's got and prescribe the minimum, so at least he stops using the more expensive and less useful pills. . . I-And that affects their health? R-Yes, he was a cardiac transplant patient, he stopped taking his medication and died. I don't know if he died due to an infection or varicella. I-And he stopped taking it because of this issue (copayment)? R-Yes, he stopped taking it because he didn't have enough money (I20).

Positive outcome

Gleeson, 2021
Study participants also recognised the importance of incident reporting in maintaining safe patient care and felt that the concept of a no-blame reporting culture was becoming more prominent in the hospital, especially since the appointment of a medication safety pharmacist.
I think the culture has changed so much. When I started you would have been hung out to dry if you made a medication error. The culture has changed dramatically over the years, that we now look at that as a learning prospect

There's a medication safety pharmacist now, and she's pushing reporting of medication errors, and there's been a two or three-fold increase in error reporting, which is great

Heras-Mosteiro, 2016
Well, you see, here I have conflicting feelings, because I'm not sure if you're actually trimming down the consumption of prescription drugs, or what's really happening is that patients who should be taking them, stop taking them because they can't pay

Physicians discussing access barriers focused on the copayment of prescription drugs, describing how patients started to inquire about the cost of medicines or requested reviews of their treatment or a change to the cheapest available option. Interviewees also described, quite astonished, cases of lack of adherence to treatment when patients chose to purchase lower amounts of drugs because of financial, rather than medical, reasons.
I remember a diabetic patient who sometimes comes with a glucose level of 400–500. I tell her, 'Maria, you must take your insulin.' Insulin is inexpensive, but you later find out that she doesn't take it, she doesn't buy it, because she doesn't have the four or five euros needed to pay for it

Kerasidou, 2019 Empathy
People don't follow prescriptions; they don't follow formulas.

Health illiteracy

Carney, 2017

Even with a permesso di soggiorno (residence permit) and current health cards, “regular” migrants and asylum-seekers face many barriers in accessing health services, beginning with misunderstanding about how to navigate the health system. Upon visiting a social worker at one of Palermo's government-operated clinics for “regular” migrants and asylum-seekers, she explains how she has been dealing with an issue all morning involving two young girls who are trying to find a place to sleep while fleeing a prostitution ring. Despite having arrived from Algeria close to two years ago, they are unfamiliar with the resources available to them, including within the healthcare system, a “sad reality of a failed system of reception” she says.

Yet language itself is not the only barrier to effective communication or efficacy of treatments prescribed in clinical settings. An Italian researcher from the field of communications claimed that with respect to non-communicable diseases, some migrant behaviors are destructive to their health, such as improper diet. She called for “nutrition education, “because [migrants] don't know what's bad for them, what's good for them.” She also alluded to drinking, tobacco use, and noncompliance with medications as barriers to effective care.

Despite arriving basically healthy, the director from NIHMP asserts that migrants and asylum-seekers develop health problems after living in Italy for one to three years, because of chronic unemployment, homelessness, stress, lack of access to health care, and violence. Even among migrants who are entitled to access the health system, he worries that many individuals do not know their rights and often defer care.

Doetsch, 2017
The high percentage of health illiteracy was frequently specified by informants to cause a great access barrier in the appropriate usage of the service in particular among the elderly population (ID1; ID5; ID7; ID8; ID11). Health illiteracy was stated to be indirectly impacted by the budget cuts under the MoU through the lack of investment on health care promotion for the elderly (ID5; ID7; ID8).
“Health literacy is a key word […] we need people participating in this system. But to people to participate they need to know how the system is organized, need to know what this system offers local, so health literacy is a key point to elderly.” Public Health physician (ID7)
Elderly were characterized to face barriers in access through: lack of understanding on the usage of health care facilities and health benefits, lack of engagement of elderly, and lack of understanding of the GP’s instructions on adequate application of pharmaceuticals

Gogishvili, 2021
Participant 6: “The admission and information desks at the hospitals need a reason why you want an appointment with the social worker. They were telling us different reasons why they could not give us an appointment with the social worker. They told us that in order to get such an appointment we already needed to have a public health insurance card. We explained that we did not have the card and that is why we wanted an appointment, but they told us that the social worker was not for that. We went there because our friend received the card this way, but in another institution or health center.”

When the interviewee was asked if the admission or information staff at the hospital explained where they should have gone to apply for the card, Participant 6 answered “No, they never told us.”

Poduval, 2015

However, migrants interviewed had poor knowledge of their health rights in the United Kingdom. Most did not know they could access primary care without payment. A woman from Uganda stated that not only was she unsure of whether health care was available without charge, she also believed that migrants were not allowed to access health services even if they were able to pay charges. "What I knew is that we didn't have any chance of getting anything, whether we pay or not, when you are illegal here you don't have any chance of getting those things. So I never tried." (12F)

Despite the fact that most migrants interviewed were not aware of their rights to primary care, many did know they could receive emergency treatment through A&E departments. The knowledge that A&E services were free led many to consider A&E as the first place to seek medical attention rather than seeking primary care from GPs, as stated by a man from Vietnam:
Interviewer: And what do you know about health care in the United Kingdom? What do you understand your rights are? Interpreter: He doesn't know. Interviewer: If he did need to go and see a doctor does he have any ideas about how he would do that? Interpreter: He doesn't know. Interviewer: So what would he do if he had a health problem that he thought needed a doctor's opinion? Interpreter: A&E. (5M) Staff described talking to GP staff that were unfamiliar with health care entitlements for migrants and trying to help undocumented migrants worried about deportation or other punishments for trying to access health care.

Balfe, 2013
The third strategy was to transfer to another clinic/ diabetes service, which usually proved to be difficult. There was often no objective information that young adults could use to determine if one clinic was better than another. As a result young adults who were considering transferring were often forced to rely on rumours or recommendations from friends or colleagues. Most searched for information on the Internet and found that clinics' websites were quite basic. Understandably a number of them were reluctant to move clinics, possibly to a worse clinic, on the basis of a rumour.

Under-developed themes/theories

Degraded working conditions (including short-term contracts) compounds staff shortages as medical staff look elsewhere for better pay and conditions (values – sticking together, fix the problem vs. self-preservation, self-worth)

Having highly skilled / experienced managers within the health service with the authority to make informed-decisions, allows clinicians to focus on delivering care, train juniors, innovate and research, retain professional integrity, skills, hands-on experience ….

But there's a tension between clinicians and management. There is a sense of solidarity between health professionals and a resistance to change (when this change is perceived to undermine the principles of high-quality healthcare)

Julian Le Grande – NHS, Knights, Naves, Pawns and Queens (Naves = agency staff)

Patient outcomes

Most quantitative studies (n=39) were looking at patient outcomes. The evidence suggests decline in patient outcomes across a range of metrics. Background section. Patients have become much more vulnerable to the next shock, the starting point is that much worse. Disproportionately affected those who are more vulnerable -

Politicians – **short-termism** (not actually that much in the literature on this but worth keeping in mind as we work through the project)

Extracts from literature organised by theme

Blame game

Ortega-Galan, 2020

The professionals feel battered by the authorities, who give no answers, restrict means and cut back on resources, and because they have to answer to the situations of users who are often in a desperate situation. This situation requires agile and effective responses that are non-existent. This situation causes people to feel frustrated, mistreated, and desperate and need to look for blame. The social worker is the professional who manages these situations and that is why he is faced with the bad being of the user who, sometimes, makes him responsible for the lack of answers to his needs.
" . . . clients sometimes blame you for the lack of resources, the lack of a solution, when really it's the fault of the authorities, but you get the blame . . . those above don't realise that the social services are debilitated, they don't realize because they're not here . . . " (GFs1, p13)

" . . . Sometimes you feel that your hands and feet are tied, because you often find that the system doesn't let you give clients what they need (. . .) and they come back . . . and in the end they take their frustration out on me . . . " (GFs2, p19)

Professionals in their speeches about the situation of the social service system wonder who is responsible or who is to blame for this situation. On the one hand, they clearly express that although professionals are sometimes socially indicated as responsible, they do not feel at all that they have generated the restrictive social policies that leave the most vulnerable populations unprotected. On the other hand, they recognize that the only ones responsible for the current situation are the public authorities that design and structure the social protection system.

The professionals assume that the public authorities that design the social policies and the public administrations that manage them are to blame for the current situation of the social protection system. However, the consequences were most severe for those most vulnerable and for frontline professionals. In addition, participants reported professional dissatisfaction due to unhappiness with the situation provoked by the system and the authorities.
"But in the end you know that it is the authorities who are responsible, they don't provide the resources or means necessary . . . " (IDIs, p6)
"There are days when I think . . . the system is a mess! Because in the end nobody takes us into account, we write a social report and unless the people in front of you are colleagues who know what it's really like, other professionals don't understand that you have described the situation because there aren't any resources" (IDIs, p5)

Distrust

Olafsdottir, 2013

however, because of selective participation and lack of transparency and documents to support the ideas, the Minister failed to establish a consensus around his proposals.
… Part of the Minister's problem was lack of cooperation. … Some of his ideas were good but the problem was that he did not cooperate with the stakeholders … even though good advice was given, it was not accepted
Healthcare specialist

Pellinan, 2018

Personnel here seem to think that management of the clinic wants to pressure them as tightly as possible. They keep saying that we've always had the extra funding when we needed it, because we

can't stop the public service. So, they want to see some representation from the city administration to come down here and confirm the crisis and the budget cuts (Head nurse).
The budget is considered unrealistic by the personnel and the financial reporting is not trusted. The overhead costs designated to the clinic in particular are seen as distorted. Management at the clinics seems to be confused by the overheads, as these are not within the control of the clinic and are apparently hard to anticipate:
I do not even understand why these costs are put to us. What I would like to see is the salaries, equipment, and materials we have used so far. This would be realistic for me. These overheads, they seem to come at an unsystematic pace. When I think that we are on budget, suddenly there comes a sudden addition of overheads designated to our clinic and all of a sudden, we're way off (Head nurse).

Well they do send the reports to us on a regular basis. I even try to follow them, but you cannot trust the reports coming from the financial administration. You can't believe the numbers. It is a constant pain to try to follow the budget. On the one hand, we're required to be cost-conscious, but on the other, these reports do not give us any way of doing it (Deputy chief physician).

Between 2011 and early 2016, there were eight general health managers, two of whom were substitutes. This issue has also been discussed many times in the local newspaper in articles concerning professional cliques, which report that doctors and their professional association resist candidates who lack a clinical background. The situation is further complicated by the expectations of the city administration and the politicians; consequently, the general health managers have found their position to be quite unstable during the last years.

[…] A shop steward for the city's doctors continues; "Doctors in the city do not trust the administration. They feel that the decisions made by the administration are in a constant flux. Also, it is clear that there is no willingness [on their part] to really get together and solve these issues [conflicts between administration and doctors] with mutual discussions" (Local newspaper article July 1, 2015).

According to the interviews, confidence in physicians and in the SNS has never faced greater risk in democratic Portugal than it faces now. Most prominent among the reasons for this change are the recurring messages from the media about the huge cuts to the SNS, affecting the salaries of health professionals as well as therapies, principally medications: So this is so ingrained that just the other day I was going to tell a patient that she was going to do radiation therapy, and she asked me: 'Doctor, order it quickly, before they stop doing it because of the money.' And I said: 'But did someone tell you that they were going to stop doing radiation?' (patient): 'Ah, they say that now they're not going to do anything.' For the patient, and right now, until proven otherwise, the problem is to save money [there is broadening distrust] Entirely! 'Why is it that I'm done and I can't do any more?' Because it is not indicated. 'Is because there's no money?' No. It's not indicated. [This question] is very frequent. Until proven otherwise, the hospital will not give a better treatment in order to save money. Similarly, until proven otherwise that anyone who comes to a hospital like this is treated poorly… a public hospital is where you do poorly. And then they say: 'By chance, I got lucky. I don't know what to say.'
The vast majority of people who say bad things are those who weren't treated there. Because those who were treated there say better things than those that never went
there, who say that it's terrible!… Until proven otherwise, the public system is bad. And will treat them badly (Physician 3

Legido-Quigley, 2013

The government changes and the eleven starting to run the (Catalan) health service are from the private sector, all of them have worked in private insurance companies . . . there is a clear intention to privatise and make business, and take a part of it. In this country we have only made houses, and there is potential to make money on health and social services. There are lots of interests behind this, there are loads of friends, there is a cloud . . . That is why there is despair."—specialist doctor, tertiary hospital

Ethical decisions

Brall, 2019

Aiming to align those ideologies on individual, party or country level would not be suitable and desirable, however ethics could help to analyse and hence better understand the respective concepts of justice and value systems in place.

Moreover, one interviewee referred to the importance of ethical decision-making especially in times of economic scarcity, with specific emphasis on upholding equity.
"I think in times of austerity the ethics of decision-making becomes even more important. Because very often one is having to make difficult decisions between spending areas or projects and so it's important when one is making most decisions one takes into account what is equitable." (P01)

Heras-Mosteiro, 2016

Exercising conscientious objection to the new regulation and continuing to
serve this population in the same manner as before did not present an ethical dilemma to most health professionals.
I have an ethical dilemma for about five seconds since I see the patient whether it is an emergency or not. Let's see, how can they tell me to refuse seeing a patient unless it's an emergency when I saw him the week before for whatever reason? That doesn't make sense to me. So, for me, it's not an ethical dilemma.

Kerasidou, 2016

The example given here of rule bending and loose interpretation of guidelines is mentioned as an important strategy in reconciling the differences between what healthcare professionals feel they are being asked to do, versus what they feel is the right thing to do. When it comes to the actual treatment of patients, they feel that some of the rules and regulations introduced are contrary to effective clinical practice and patient management. This, they feel, makes their everyday working lives difficult.

Tucker, 2020

We have patients who can't leave hospital now, because they haven't got any input in the community, and from a duty of care point of view it's a real ethical/moral dilemma for therapists (emphasis added as shown in bolding).

Financial accountability

Pellinan, 2018

Further, as budget responsibility has not been delegated formally to the deputy chief physicians in charge of the clinics, this creates an ambiguous situation concerning the meaning of financial accountability and may further promote emphasis on professional accountability at the operational level:

This under budgeting has been our way for years now. We just ask for extra funding every year when we realize that the budget is not going to hold. And I understand it now, the budget doesn't mean a thing; extra funding comes anyway (Head nurse

Previous budgeting mistakes, ever increasing cuts to the budget and a lack of understanding about the principles behind the calculation of the overhead figures add to the ambiguous situation the clinical professionals perceive. This has led to the clinical profession to emphasize professional accountability in spite of the city administration's aim to increase the financial accountability in the organization. Further, clinical professionals direct blame toward the financial administration and city management for previous mistakes: We had this error in the budget a while back. One cost item was left out of the budget, which amounts to over one million euros. You can think for yourself what this means to us. We have to stay on budget, while those making the budget leave out some numbers. We still have to pay the salary, even for those that are not included in the budget (Director of basic health care).

In our case organization, one of the problems resulting in part from a restriction on hiring substitute personnel is that most of the clinics are understaffed. This results in disproportionate overhead costs being attributed to a single patient visit, thus causing sub-optimization and distorted cost calculations.

One aim of such strategies seen in our case is to downplay financial accountability, action that comes close to resembling civil disobedience in terms of the vertical hierarchy
I think it was about one and a half years ago that the mayor was talking to the clinical management team and said that the situation is dire, the situation needs to be improved, both in terms of mutual relationships and of budget control. He also said that if someone thinks that they're not accountable, or they don't want to be, they don't have to be here (in the organization). And I was there, too. And I looked at the expression on their faces [clinical professionals] and I already knew they weren't going to change a thing (Deputy chief mayor).
This lack of dialogue between city administration and clinical professionals suggests that when two strong accountabilities conflict and common goals and blame sharing are absent, different parties reconcile the situation by utilizing the blame avoidance strategies. One aim of such strategies seen in our case is to downplay financial accountability, action that comes close to resembling civil disobedience in terms of the vertical hierarchy. Another implication in our case is that loyalty to the profession and an individual sense of responsibility is stronger than loyalty to the organization, at least when the organization is both vertically and horizontally complex.

Fana, 2021

While this committee enabled finance officials to control expenditure, clinical staff regarded it as a barrier to service delivery: 'To buy just a simple thing like electrodes which only costs (USD 60) we have to go through many steps. We have to sit as an Institutional (hospital) Costs Containment Committee on Monday, and then send our request to the District Costs Containment Committee that sits on Tuesday, and then on Wednesday sent it to the Provincial Costs Containment Committee. We then wait for their approval or disapproval. This has increased the turn- around time' (Senior Manager

Governance

Carney, 2017

Instead of requesting an emergency rescue loan to "weather the
sovereign debt crisis," (Oxfam, 2013:2), that others astutely note was actually a global banking crisis (Muehlebach, 2016), Italy has been repeatedly pressured by fellow EU member-states into economic austerity. Recent austerity measures implemented by the Italian government include increases in taxes on households (to 45 percent of gross income by some estimates), a delay in the age of retirement,

lower pensions, reductions in wages, a reintroduction of property taxes, and significant reductions in national public spending for social programs

Kentikelenis, 2014

Two main strategies can reduce deficits in the short term: cutting of spending and raising of revenue. The Greek Government used both at the behest of the Troika, albeit with an emphasis on reduction of public expenditure. 3 years ago, we drew attention to the effects of the austerity measures on the health of the Greek people.8

Hollingworth, 2015

Observed meetings were dominated by new requests for funding and implementation of NICE mandates

Leider, 2014

the underlying political environment and the structural challenges of state budgets being under the control of the governor and legislature and, significantly, the underlying political environment.

Thomas, 2013

"The New Programme for Government has an 'entirely different focus'– Universal Primary Care and Universal Health Insurance - but Government knows we are no-longer masters of our own destiny – so how these can be delivered is a question."

Cervero-Liceras

The perception of corruption and mismanagement was
often mentioned in interviews and at all levels of the health care system. At facility level, interviewees reported drugs being taken home regularly and heard cases of expensive equipment being stolen from public hospitals. Professionals working in the private sector reported knowing of cases of corruption within their centers. There were particular concerns about the potential for corruption in private concessions delivering public services, with frequent calls for tight control of these arrangements during interviews. At the system level, politicians, policy makers and top
level management were blamed for not having the knowledge, experience and information needed to take decisions that would improve the health care system. Moreover, most interviewees perceived a lack of planning and claimed that decisions were made precipitously.

Healthcare professionals complained of being excluded from the design and implementation of healthcare policies. Participants highlighted a lack of consensus in the introduction of healthcare reforms and a low level of engagement with associations and citizens. In addition, they expressed concerns regarding a lack of transparency and communication of the new
policies. As a result, health care reforms were believed "to be doomed to failure". In the following quote a doctor describes how decisions are normally made by those who do not know about the topic: So the clinical consequences are not taken into account because (the decisions) are not taken by those who know about the topic. Of course! (I05).

Fana, 2021

Hospital A is a 350- bed specialised provincial tuberculosis referral hospital with 200 staff, and it reports at the district and provincial level. It has experienced instability in leadership with three CEO appointed in the last 5 years and had lost experienced employee in its management team.

In response to austerity, the provincial department of health centralised all financial decision- making, introducing a provincial cost containment committee (PCCC) that reviewed every expenditure decision.

While this committee enabled finance officials to control expenditure, clinical staff regarded it as a barrier to service delivery: 'To buy just a simple thing like electrodes which only costs (USD 60) we have to go through many steps. We have to sit as an Institutional (hospital) Costs Containment Committee on Monday, and then send our request to the District Costs Containment Committee that sits on Tuesday, and then on Wednesday sent it to the Provincial Costs Containment Committee. We then wait for their approval or disapproval. This has increased the turn- around time' (Senior Manager

Antunes, 2019
Finally, users mentioned the need to clarify leadership structures and accountability of health and administrative professionals.
"I think they should have a hierarchical superior in the health centre". (man, 66 years old) "One fundamental thing is to have someone accountable here. . . even a director". (woman, 62 years old)

Lack of transparency

Olafsdottir, 2013

however, because of selective participation and lack of transparency and documents to support the ideas, the Minister failed to establish a consensus around his proposals.
… Part of the Minister's problem was lack of cooperation. … Some of his ideas were good but the problem was that he did not cooperate with the stakeholders … even though good advice was given, it was not accepted
Healthcare specialist

Cercero-Liceras, 2015
In addition, they expressed concerns regarding a lack of transparency and communication of the new policies

Transparency, there is very little. Transparency is the "quid" of the issue. Well, transparency does not exist. But it does not exist at a hospital level and I don't believe either at political level or national level. . . (I17).

Fana, 2021
In both Hospitals A and C, poor communication led to conflict. In Hospital A, the lack of communication that applications for new staff had been rejected led to animosity between departments: 'It's a lie. Human Resources Management staff are not doing their job, yet they expected us to deliver and meet our targets; how?' (Senior Manager Hosp. A). A lack of information about an outbreak of Klebsiella led non- clinical staff to believe that management and clinical staff were intentionally exposing them to infection, when they did not inform staff about outbreak and took precautionary measures to protect themselves

Poor communication and a lack of understanding led one manager to believe that his posts were intentionally not filled and that payment of monies due to him were blocked by another manager. The result was a physical fight between the two managers

Union- management relations were strained in Hospitals A and C. In Hospital C, the relations between labour and the CEO are poor: 'We need to work on that, because sometimes even meetings are not

held as per the schedule' (Senior Manager Hosp. C). In Hospital A, senior management did not seem to recognise the importance of the union. The unions had decided not to engage with the CEO anymore

Communication

Fana, 2021

In Hospital B, there was better management, leadership and communication than in Hospital A and C with cohesion and teamwork among members of the top management. The management (CEO and the Director Human Resources) had been recently appointed in 2016 and had introduced more democratic management and open communication: 'We communicate with all the stakeholders regularly. All of them know what is within our control in the hospital and what issue are out of our control' (Senior Manager Hosp. B). A shop steward described the relationship with management: 'The CEO consults and involves unions in planning and when things do not go as planned, CEO comes to explain; CEO knows how to work with unions' (FGD: Shop steward Hosp. B

A junior employee highlighted how this communication increased cohesion: 'If you have something that does not sit well with you, you can easily say it without fear of what will happen to you, so that it can be sorted out immediately; we are like one family, and even if someone is facing a disciplinary action, our managers don't abandon us, they support and defend us during that process' (FGD: Nursing Assistant Hosp. B)

In both Hospitals A and C, poor communication led to conflict. In Hospital A, the lack of communication that applications for new staff had been rejected led to animosity between departments: 'It's a lie. Human Resources Management staff are not doing their job, yet they expected us to deliver and meet our targets; how?' (Senior Manager Hosp. A). A lack of information about an outbreak of Klebsiella led non- clinical staff to believe that management and clinical staff were intentionally exposing them to infection, when they did not inform staff about outbreak and took precautionary measures to protect themselves

Poor communication and a lack of understanding led one manager to believe that his posts were intentionally not filled and that payment of monies due to him were blocked by another manager. The result was a physical fight between the two managers

Thirdly, a regular bilateral forum was created to allow finance and human resources managers to resolve challenges: 'It is not a formal structure, but something that we initiated, and its success depends on our willingness to cooperate with each other to resolve issues' (Senior Manager Hosp. B). HR and finance staff were able to learn more about how their individual tasks fitted into broader processes, and how challenges arose. Salary queries were resolved more quickly and accurately, and employee satisfaction increased.

Gleeson, 2021

Communication within medical teams was considered key to ensuring patient safety during a hospital stay, while communication with community healthcare services, including general practitioners (GPs), was equally important in maintaining patient safety once the patient had left hospital. If there isn't communication among team members then there is going to be a slight kind of break in the link chain of the patient's actual clinical management, and that then could affect the patient safety in different ways (HSCP 5)
I suppose proper communication that … if you send out a letter to a GP, that the GP gets it and that

you know that the GP has gotten it (Physician 4)

How decisions perceived / framed

Hollingworth 2015

The scope for public accounts, and thus bias, was a prime consideration throughout the conduct of this study, and commissioners' portrayal of previous local practices was no exception. Even if the above commissioners rejected the term 'disinvestment' in a public context, the mere desire to dissociate local practices from 'disinvestment' suggests some level of concern that disinvestment carries negative connotations.
There was one example of disinvestment being presented differently in individual (interview) versus group (meeting) contexts. Despite describing threshold policies as examples of disinvestment within their
interview (particularly the 'cataract policy'), the commissioner below tried to dissociate these policies from 'disinvestment' when addressing the group:
There are various grades of disinvestment . . . threshold policies are a kind of disinvestment. Interview, group A, PCT1, C1

So I'm going to share some of the work we do, what our system is, how we involve the providers, how we work collaboratively . . . and also a couple of [threshold] policies, [. . .] and also the cataract policy. So – not disinvestment, but a proper use of resources.
Observation, group A, PCT1, C1

Leider, 2014

More typically, interview respondents identified the governor or legislature expanding Medicaid while cutting public health. These respondents attributed that action to the increase in match/maintenance of effort requirements and the political ramifications of Medicaid cuts, versus relatively fewer ramifications for public health cuts, especially during the post-2008 recession.

Olafsdottir, 2013

The Minister of Health requested preparedness for increased demand in health services, particularly in mental health departments, to meet the increased distress. A new free psychiatric service was opened and, at first, 1–4 individuals used the service per day, but that number soon decreased. Approximately 50 individuals used the service during the 6 months it was open, less than two individuals per week on average. Some interviewees perceived the opening of this free service as a political propaganda as one of the interviewees said:
[The psychiatric reception] was opened with some pomp and circumstance … a new psychiatric reception is not what people need at this stage. When the nation is experiencing disaster, like this, people need information, not psychiatric-nonsense. … People did not know what was happening to their country and what the consequences for its inhabitants would be.
Healthcare manager (emphasis added

All key informants stated that political views had a much stronger influence on actions and decisions than the opinions of health professionals: The political advisors that Ministers of Health have in their closest circle have had much more influence on policy making than health professionals … The policy making process runs down the wrong paths, delivering disputable results.
Healthcare specialist

Pellinen, 2018

[…] A shop steward for the city's doctors continues; "Doctors in the city do not trust the

administration. They feel that the decisions made by the administration are in a constant flux. Also, it is clear that there is no willingness [on their part] to really get together and solve these issues [conflicts between administration and doctors] with mutual discussions" (Local newspaper article July 1, 2015).

Balfe, 2013

A number of young adults felt that the Irish healthcare system was increasingly concerned about providing them with the cheapest care rather than the best care. One young woman, for example, noted that auditors in the health system questioned her pharmacist to find out if she could be provided with fewer glucose test strips. A number of young adults also felt that pharmaceutical companies were beginning to fund functions that had previously been standard aspects of public sector care, such as glucometers and diabetes training programmes. About a quarter of young adult interviewees noted significant difficulties in obtaining diabetes technologies, particularly CSII and when they were offered these devices they were not given a choice about the model they could take. They felt that the health system restricted their ability to obtain the most advanced CSII devices that, although more expensive, would give them the best quality of life. One young woman who attended an urban teaching hospital in Dublin, however, noted that her consultant gave her a choice of three pumps from which to choose.
The chemist said do you need that many strips. I'm like, yeah. They're like, there was a query about it. I'm hardly selling them on the street. (Female, 28).
You don't get the best pump. You get the old one that costs less. (Female, 27).
I was on the list for the diabetes programme for 18 months. They just don't have the resources. It's a pharmaceutical company that are paying for it now. (Female, 30).

Informed decision making

Hollingworth, 2015

Commissioners tended to portray restrictive policies as tools for minimising wasteful use of NHS resources. They reported achieving this by ensuring the provision of treatment led to evidence-based clinical benefit. The following portrayal of 'not routinely funded' policies was typical of commissioners; here, the decision to stop funding activity is presented as logical and non-contentious:
It's pretty much a no-brainer to say that something doesn't work, therefore we shouldn't be doing it [. . .]. You get a body of experts to look at the evidence base and say, 'That's rubbish, don't do it'. That's fine.
Interview, group A, PCT1, C2
Threshold policies were also typically presented by commissioners as indisputable choices that made intuitive sense. Most commissioners avoided any suggestion that policies denied care, emphasising that criteria ensured that patients who would benefit from treatment would still be granted access:
We're just turning back the tide of the ones that shouldn't be [receiving treatment]. Interview, group B, PCT2, C10

Although service configuration as a form of disinvestment was not apparent during the period of observation, a mix of commissioners and providers from both regions discussed examples of it. Past initiatives included changing the way in which treatments were delivered, and closure of satellite health centres in favour of centralising services:
So it could happen at an organisational level. So there's been examples where services have been centralised . . . so all the clinical evidence would suggest that the more of something that someone does, the better the outcomes will be.
Interview, group A, PCT1, P3

Leider, 2014

“We look at effectiveness, cost, evidence, acceptance of the programs and immediate impact vs long-term impact. We use those elements for making decisions. How do we choose between STI clinics and community health services for pregnant women and children. We choose the latter, based on the above criteria.” (Senior deputy)

In the absence of an expectation of increased political support to alleviate levels of scarcity driving the frequency or difficulty of trade-offs, one potential avenue for future research might turn toward the creation of training, technical assistance, and decision support tools to aid in systemizing (and potentially easing) these difficult trade-offs practitioners encounter on a regular basis, as well as aids for practitioners to navigate rough political waters.

Pellinen, 2018

Municipal politicians expect the organization to provide good quality services at minimum cost. The municipalities expect to be provided with timely financial reports on the different areas of the FHC to assist their oversight responsibilities. However, some of the municipalities in the FHC were unhappy about the financial reporting provided by the main city:
[…] member municipalities need to be informed better of the developments in the FHC during the fiscal period. Timely and precise reports are needed to ensure that member municipalities can keep track of the expenses. […] a representative [of one of the member municipalities] stated “we shouldn’t be forced to dig out the information from databases ourselves and try to calculate the different cost units from pooled data. For example, last year [2014] our expenses [from the FHC] were bigger than the agreed budget” (Local newspaper article February 24, 2015).

A new manager, responsible for health care services, tried to find solutions to the situation by holding both personal and small group discussions over three months with the staff of the FHC. In an effort to reconcile conflicting accountabilities, the manager also had a great deal of dialogue with the decision makers on the basic welfare board, aiming to teach them to recognize the health care issues affected by the decision-making processes. This implies the (as yet unrealized) potential of dialogue between professional groups to reconcile conflicting accountabilities: I had some good discussions with the basic welfare board, and I tried to bring the information to the decision makers from the viewpoints which are important in health care […] also there was some understanding, at least partly, about the financial aspects. But then again, when we went to the budget negotiations in the autumn, it was never realized in practice, because the decision makers saw that now we have the budget frames here and we cannot go over these frames, even though we know what should be done (General health manager).

Political ramifications

Kentikelenis, 2014

A new manager, responsible for health care services, tried to find solutions to the situation by holding both personal and small group discussions over three months with the staff of the FHC. In an effort to reconcile conflicting accountabilities, the manager also had a great deal of dialogue with the decision makers on the basic welfare board, aiming to teach them to recognize the health care issues affected by the decision-making processes. This implies the (as yet unrealized) potential of dialogue between professional groups to reconcile conflicting accountabilities: I had some good discussions with the basic welfare board, and I tried to bring the information to the decision makers from the viewpoints which are important in health care […] also there was some understanding, at least partly, about the financial aspects. But then again, when we went to the budget negotiations in the autumn, it was

never realized in practice, because the decision makers saw that now we have the budget frames here and we cannot go over these frames, even though we know what should be done (General health manager).

Kentikelenis, 2014

Greek Government officials, and several sympathetic commentators, have argued that the introduction of the wide-ranging changes and deep public-spending cuts have not damaged health and, indeed, might lead to long-term improvements. Officials have denied that vulnerable groups (e.g., homeless or uninsured people) have been denied access to health care, and claim that those who are unable to afford public insurance contributions still receive free care.

Leider, 2014

"Our legislature has a 25% to 40% turnover rate every 2 years. Political interests change much more quickly than public health interests and we have to find better ways of making healthy choices easier for politicians to make." (Legislative

But as interview and survey respondents made clear in the course of this project, trade-offs in public health priority setting are grounded in an authorizing environment with a driving economic need and political desire to ration government support to a variety of social services; public health is but one of many services in this context.

Olafsdottir, 2013

The protests on the streets amplified over the
months, and in January 2009 the Prime Minister resigned, the cabinet stepped aside, and a new government was formed, which took control on 1 February 2009. The two politicians who exchanged keys to the Ministry of Health on 1 February 2009 favoured radically different political ideologies. It is, therefore, not an overstatement to say that the Ministry of Health went from one political extreme to another.

Pellinen, 2018

Politicians in the city council and on the basic welfare board tend to think in terms of the meeting they are participating in. This results in contradictory behavior. And there are so many different agendas, I mean, it's not only the party politics that determine politicians' opinions, it is also regional politics, so politicians may be inclined to prevent the closure of a clinic in their constituency (General health manager).

The clinical administration of the FHC is of the opinion that in this situation delivering cost savings in health services would require shutting down some of the health care clinics. However, any proposal to close some of the clinics results in political debates and, ultimately ends up being blocked by the politicians on the various boards of the city administration. Politicians seem to be reluctant to take the responsibility for a decision to weaken services, since that could weaken their chances of being re-elected: Whenever we try to make a big decision about service coverage, like shutting down a couple of clinics to cut costs, we encounter political resistance and they keep saying that we must leave the service coverage untouched. It is difficult for a health manager to make any tough decisions when you don't get political support (General health manager).

The relocation of officeholders is one example of the agency strategy for avoiding blame in Hood's (2011) typology. This strategy can be utilized in the form of dismissal (to shift blame to the person

being dismissed), and by resigning, thereby avoiding a blame falling on oneself:
At the time, they (politicians) saw the manager's responsibilities in the organization as simply to be a cost-cutter. They didn't appreciate cost-effectiveness, as in doing an effective job or thinking about long-term cost effects, rather they wanted immediate cost efficiency. And now that I think about what has happened since (the relocation), I can already see that in one year, they (the FHC) are already overrunning the budget more than ever (Relocated general health manager).

Thomas, 2013
Up to 2012, HSE have managed to keep their overall
budget balanced, but this masked a serious problem of overruns in hospital spending as budgets fell but political pressure on waiting lists forced more hospital activity.

Performance monitoring

Correia, 2015

The monitoring of public hospitals' performance and of drugs prescription through compulsory electronic procedures intended to limit doctor's autonomy.

A substantial reduction in drug expenditure in the NHS was achieved mainly through introducing clinical guidelines, monitoring systems, compulsory electronic prescription and giving priority to generic drugs in both the public and private sector.

Physicians' working hours were increased to 40 h per
week, while the remuneration of general practitioners became more performance-based

Gleeson, 2021

Study participants also recognised the importance of incident reporting in maintaining safe patient care and felt that the concept of a no-blame reporting culture was becoming more prominent in the hospital, especially since the appointment of a medication safety pharmacist.
I think the culture has changed so much. When I started you would have been hung out to dry if you made a medication error. The culture has changed dramatically over the years, that we now look at that as a learning prospect

Kerasidou, 2019 Austerity

"So it's (the four-hour target) not an indicator of quality at all. [. . .] it's a bit of waffle isn't it? And you could say, right ok, you are good quality because you get hardly any complaints, or incidents, or most patients have a safe journey through the A&E department. All those things are really important quality indicators. So the performance target is just a measure of time. It is not a measure of anything else. And they use it because it is easy to measure. But you could argue that if people spend longer in the department, then they get more treatment, we may have more time to spend with them. They may get a better deal. So a four-hour target itself is not a quality measure

Kerasidou, 2019 Empathy

As Nurse 1 explains, the main reason why meeting targets is important in A&E is because these measures are directly linked to funding. If a hospital fails to achieve its targets, it is penalised by having its budget reduced:
Cause there is so much pressure to meet the targets and that's what it comes down to. [...] The risk of going into special measures, you lose a lot of funding and you get a lot of scrutiny on the Trust as a whole. (Nurse, 1)

Opportunity to reform

Pellinen, 2018

Modern patient work involves input into different clinical databases. This, in turn, has enabled the city management to push financial accountability in the form of various performance measures to the clinical professionals irrespective of the budgetary control of individual clinics.

Correia, 2015

A substantial reduction in drug expenditure in the NHS was achieved mainly through introducing clinical guidelines, monitoring systems, compulsory electronic prescription and giving priority to generic drugs in both the public and private sector.

Kentikelenis, 2014

There is a broad consensus that the social sector in Greece was in grave need of reform, with widespread corruption, misuse of patronage, and inefficiencies, and many commentators have noted that the crisis presented an opportunity to introduce long-overdue changes.

Thomas, 2013

For sustainability we need new responses (how do we deliver care, what is the burden of responsibility etc.) … this is an opportunity for fundamental change”.
The Programme for Government outlines for the first time in the Irish state the principle of universal access to health care through a Universal Health Insurance system, drawing on the Dutch model
