# Supplementary material for: How International Health System Austerity Responses to the 2008 Financial Crisis Impacted Health System and Workforce Resilience – A Realist Review
Source: Int J Health Policy Manag. 2022 Dec 24;12:7420. doi: 10.34172/ijhpm.2022.7420 (PMC10125082; doi:10.34172/ijhpm.2022.7420)
Supplement: Supplementary file 3 — Theoretical Lines of Enquiry From Additional Searches. [file ijhpm-12-7420-s003.pdf]

Article title: How International Health System Austerity Responses to the 2008 Financial Crisis Impacted Health System and Workforce Resilience – A Realist Review

Journal name: International Journal of Health Policy and Management (IJHPM)

Authors' information: Padraic Fleming[1]*, Louise Caffrey[2], Sara Van Belle[3], Sarah Barry[1], Sara Burke[1], Jacki Conway[4], Rikke Siersbaek[1], David Mockler[5], Steve Thomas[1]

[1]Centre for Health Policy and Management, Trinity College Dublin, The University of Dublin, Dublin 2, Ireland.

[2]School of Social Work and Social Policy, Trinity College Dublin, The University of Dublin, College Green, Dublin 2, Ireland.

[3]FWO, Institute of Tropical Medicine, Antwerp, Belgium.

[4]Everlake, 5 Marine Terrace, Dun Laoghaire, Dublin, Ireland.

[5]Library Reader Services, Trinity College Dublin, The University of Dublin, St James Hospital, Dublin 8, Ireland.

(*Corresponding author: Padraic.fleming@tcd.ie)

Supplementary file 3. Theoretical Lines of Enquiry From Additional Searches

Contents

Appendix 3 – Theoretical lines of enquiry 1
CMOC 1 - Top-down governance: 1
Search Summary: 2
CMOC 2 – Perceived Value shift 5
Search summary: 5
CMOC 3 – Powerless and detached 12
Version 2 12
Search Summary: 12
CMOC 4 – Working the system (access) 20
Version 2 20
Search Summary 20
CMOC 5 – Health-seeking Behaviour Change 29
Search Summary 29
First redrafting CMOCs after parallel searches 36
References 39

CMOC 1 - Top-down governance:
Health systems decision-making was highly influenced by outside agents (e.g. TROIKA) within the broader austerity agenda, but there was a lack of transparency about these outside influences from policy makers, compounded by poor communication from

management and lack of co-production with frontline staff, led to a lack of ownership and buy-in from those delivering care and a distrust of the decision-making agenda.

Search Summary: Title: transparency AND health = 10 results (7 potentially relevant, 4 full texts available)

1. **Al Balushi, 2020 - How internal transparency impacts organizational resilience**

Hood (1995) shows that as a result of [New Public Management] NPM, transparency has gained more prominence since clarity in role assignment and authority has become essential. Besides, NPM has emphasized the setting of measurable standards and control mechanisms, based on the practices prevalent in the private sector. According to Lapsley (2009) and Lapsley and Rios (2015), with NPM, more emphasis has been placed on transparency. Hood and Heald (2006) perceive transparency in contemporary public management "quasi religious." Though one might argue that the abovementioned measures could have aimed at transferring blame or accountability, they still have impacted public management.

Albu and Flyverbom (2016) distinguish between two essential epistemologies in transparency research, namely, verifiability approaches and performativity approaches, with the former being more affirmative transparency and the latter being more critical.

According to Heald (2012), "transparency is a mechanism through which the principal can exercise surveillance over the actions of an agent." The emphasis over "principal-agent" surveillance limits the true benefits/value transparency introduces. It sounds more like a control mechanism rather than an enabler of better management. Heald adds that in symmetric relations, both the principal and agent can view the processes of each other. Beech and Crane (1999) call it multidimensional transparency. However, in many cases, transparency ends being one-sided (top-down). Looking at its significance, prominent international bodies (e.g. IMF and OECD) stress the importance of transparency in public finance, for instance (Lapsley and Rios, 2015). The literature introduces external and internal transparency. Street and Meister (2004) state that external transparency is communication to the environment outside the organization. It aims at ensuring outsiders, normally a specific group, are aware of certain organizational activities. However, Maclean (2011) reminds that whatever the volume of external reporting is, it cannot replace internal transparency.

Street and Meister (2004) define internal transparency as "an outcome of communication behaviors within an organization that reflects the degree to which employees have access to the information requisite for their responsibilities."

Street and Meister (2004) show that as communication level is reduced, internal transparency follows in the same direction. They demonstrate that reduced internal transparency results in difficulty as management becomes less aware of issues within the units around them. Again, reduced internal transparency affects all stakeholders, not management alone, but it depends on the quality of information indeed.

On the other side, scholars have also identified negative consequences for transparency. For example, Christensen (2002) shows that managers, when dealing with corporate communication, appear to take it for granted that their organizations are transparent. This "false transparency" is in itself a by-product of the lack of transparency.

Albu and Wehmeier (2014) illustrate that transparency can become meaningless to stakeholders when it focuses on enhanced communication flow rather than enhanced understanding.

Discussion

With internal transparency enhanced, the results indicated improved resilience. As extensive documentation and reporting was required, alongside the use of performance indicators, context analysis and scheduling, risk identification and prioritization was necessitated and introduced. Risks were identified and monitored using indicators. The documentation, reporting and indicator systems enabled exchange of knowledge and continuous improvement, which resulted in organizational learning. Sensemaking was also improved as beneficiary requirements were the basis of both fact-based management and performance indicators.

2. **Griffiths, 2018 - Resilience and transparency in social systems**

Neoliberalism and resilience

neoliberalism, which will serve for present purposes: [...] a theory of political economic practices that proposes that human well-being can best be advanced by liberating individual entrepreneurial freedoms and skills within an institutional framework characterized by strong private property rights, free markets and free trade. The role of the state is to create and preserve an institutional framework appropriate to such practices [...] and to guarantee, by force if need be, the proper functioning of markets. Furthermore, if markets do not exist (in areas such as land, water, education, health care, social security or environmental pollution) then they must be created, by state action if necessary. (Harvey, 2005, p. 2)

Youssef et al. explore this enframing, and argue that neoliberalism has brought about a shift in discourse from "resistance" to "resilience": Building resilient subjects involves the deliberate disabling of the political habits, tendencies and capacities of peoples and replacing them with adaptive ones. Resilient subjects are subjects that have accepted the imperative not to resist or secure themselves from the difficulties they are faced with but instead adapt to their enabling conditions. (Youssef et al., 2013, p. 85)

The second link between neoliberalism and resilience concerns transparency and, by extension, accountability. Transparency, understood as the degree to which others can inspect the activities of an individual or organization, is associated with many positive esthetic, social and political experiences. It can prevent bullying, corruption and abuse, as well as facilitating understanding and collaboration and improved standards in public life.

At issue here is not the existence of accountability, but rather the nature of the accounts, and the degrees of transparency and constraint on autonomy which are required by and of managers, employees and politicians. Key performance indicators have become ubiquitous as the means of imposing transparency and accountability in management in the USA and UK, as well as other countries. They were defined by Fitz-Gibbon at a relatively early stage of their deployment: [...] as an item of information collected at regular intervals to track the performance of a system. Performance indicators (PIs) are collected in many complex systems which, like education, deliver a service. Thus 'near-misses' are recorded to monitor air transport; the length of hospital waiting queues is recorded by the Health service. (Fitz-Gibbon, 1990, p. 1) Selective transparency is generated by key performance indicators, and this enables managers to control the activities of the managed. The

relevant questions for the present inquiry are how neoliberalism has changed the reporting conditions for accountability, how the balance between specified autonomy and imposed transparency has changed as a result, and what the implications of these changes are for resilience.

Within a hierarchical social structure, the law of requisite variety (Ashby, 1958) determines that not all the details of the functioning of the managed system can be exposed to the manager. There are therefore black boxes (Glanville, 2003) within the system where action is taken independently of the manager's control. … Within black boxes, interpersonal and professional practices arise that maintain the viability of the organization by adsorbing the variety generated by the activities that occur within the black box. To take the example of education, teachers carry out many undocumented tasks which are invisible to educational managers, such as maintaining mental models of all their learners and adapting their communication with them accordingly, adapting learning materials to the learning needs of individuals or groups, responding to learners' personal crises, or eliciting a positive social environment in the classroom. All these activities take place within a black box, as far as management is concerned.

It is the blackness of the boxes in social systems that provide the flexibility which is necessary to sustain the coexistence of contradictory models of the processes underway in social systems.

In the example of teaching policy, variety equations make it hard, or impossible, for policymakers to perceive the problems of teachers and learners. These problems can be addressed in conversations between teachers and with learners that take place in an unrestrained way within black boxes, enabling responses to emerging problems to be developed and elaborated. For these conversations to take place, flexibility is needed in both time and activities. However, if the black box becomes transparent because of the introduction of management philosophies and methods informed by neoliberalism, then those active in the black box will be required to be accountable to management using a set of metrics to which financial value is eventually attached. The adjustments in the balance of reporting and autonomy which are designed while carrying out this process will not support conversations whose purpose is not perceptible from the standpoint of the manager.

Black boxes enable multiple conversations to take place, generating multiple perspectives and interventions with which to meet the challenges generated by continual external change. A design initiative by management to increase transparency and accountability, without respecting the historically evolved network of black boxes that maintains organizational viability, is therefore likely to negatively impact on the resilience of the system.

These considerations apply to all organizations, but are particularly severe in education, because those involved ascribe a wider range of purposes to the organization than the case in most institutions. These purposes include personal growth, employability, the award of qualifications in return for payment, the health of the economy and the preservation of national cultures. Many of the conversations and activities which take place to address these conflicting purposes are undocumented and take place in black boxes. Opening up these black boxes to management control based on a subset of educational purposes may be expected to suppress those undocumented purposes, and to suppress functions which are valuable to individuals and society. Constraints to the conversations within black boxes can be catastrophic. Outsourcing of government work to the private sector is in effect a dramatic reduction in conversation between government and those who carry out work in its name. In outsourcing, activities within the existing black boxes of a system are

not made transparent, but rather completely eliminated, and replaced with other black boxes external to the system.

I have argued that increases in transparency and accountability, when deployed within a neoliberal context, impoverish the interactions of organizations, employees and stakeholders, concentrate problem solving at the pinnacle of the organization, and may gradually redefine the purpose of the organization. The neoliberal reform program frames the problems that emerge in terms of resilience and seeks to promote resilience by exposing all actors to the Darwinian rigors of the market. It is argued here that, on the contrary, not only do some organizations fail to survive market competition, but also the strategies applied to promote participation in markets, i.e. the promotion of transparency and accountability, can themselves constrain the maintenance of viability in complex social organizations. Political and social action may be necessary to correct these problems. Nevertheless, the analysis presented contributes to a possible solution in two ways: first, by clarifying the need for the recognition of the hidden contribution to the maintenance of organizational and individual viability made by undocumented conversations and interactions, and, second, by showing the need for close analysis of the systemic contribution made by the conversations and interactions which take place in black boxes before opening up those black boxes to managerial accountability.

CMOC 2 – Perceived Value shift

In context of restrictive fiscal policies (staffing, consumables, treatment options, available time with patient), a perceived value shift is evident for health professionals, from patient-focused to economic, with long-term consequences resulting in a diminished view of the profession, apathy and burnt-out.

Search summary: Title words: values AND health Keywords: burn-out OR austerity = 22 (16 potentially relevant, 9 full texts available)

3. **Benatar, 2010 - Values in global health governance**

The dominant values that define understanding of global health in the rich ‘western’ world can be epitomised as: individualism and respect for human rights, economic liberalism, corporate managerialism, a narrow focus on scientific rather than social solutions to health problems, and an oversimplified, linear approach to health problems. The prominence given to individualism and respect for human rights has become dominant only since the Universal Declaration of Human Rights which, it should be noted, defines all rights as inalienable and interdependent (Second Interagency Workshop 2003). However, within western societies the values of self-determination, civil and political rights and economic freedom, generally rank above socioeconomic rights, including access to health care (Chapman 1994, Falk 2000, Teeple 2000, Benatar 2002, Farmer 2003). Failure to achieve socio-economic rights is also a function of the lack of effective governance at the national level to compensate for some of the unequal distribution of the spread and growth of global capitalism (Benatar 1998, 2005, Pogge 2002, Bakker and Gill 2003).

Our 60-year focus on individual autonomy and interpersonal ethics is now being gradually supplemented by an expanding understanding of the need for an ethics of public health to enable us to deal rationally with threats to global health as systemic challenges (Dawson and Verweij 2007). This invokes a wider view of the determinants of health and the local and global actors that must

engage these issues (WHO 2008) and requires us to look beyond the proclamation of rights and values to examine the ways they are applied and interact in practice. It also challenges us to reevaluate, redefine and rebalance these values (Buchanan 2000, Benatar et al. 2003, Nixon et al. 2008).

4. **Lachman, 1984** – **A STRESS MANAGEMENT SUPPORT GROUP AS INPUTS IN REDUCING NURSING STRESS AND IN CHANGING HEALTH VALUES: AN EXPLORATORY STUDY** - PHD thesis preview only

Values and beliefs play a large role in determining someone's perception. Jones (1980d) found that greater endorsement of irrational beliefs significantly correlated with higher levels of staff burnout. Other studies suggest that internally oriented (perceive self as cause) individuals may be better able to handle stress from uncontrollable events.

Irrational beliefs are certain values or ideas common in our culture that lead to anxiety, anger and depression. An example of an irrational belief is, "It is essential that one be loved or approved by virtually everyone in his community." Even though this belief is unreasonable, many people hold it to be true to some degree. Rimm and Litvak (1969) found that this negative self-talk produced substantial physiological arousal (stress).

5. **Landwehr, 2015 - Value congruence in health care priority setting: social values, institutions and decisions in three countries**

There is a broad body of philosophical literature on the hard choices to be made in the comparative evaluation of claims to medical services under scarce resources, which cannot be recapitulated here (but see Daniels, 2008 for some vivid examples). What is relevant in this context is that governments in most OECD countries have delegated the intricate task of weighting competing principles of just distribution in the assessment of medical technologies to specialized agencies, which are charged with defining lists of medical services, or health baskets, covered by the public health care system (Landwehr and Böhm, 2011). These agencies operate at arm's length from the government and bureaucracy and are often statutorily independent. However, they are inscribed with values and programmed with distributive principles they are to apply in decisions. Such inscriptions may be explicitly spelled out in the mandate and statutes of an agency, or they may be implemented through institutional design. To use a somewhat blunt example: an agency staffed exclusively with health economists is more likely to apply efficiency considerations than one that is staffed with patient representatives. As Landwehr and Böhm have argued and demonstrated elsewhere (Landwehr and Böhm, 2011; Böhm et al., 2014), the institutional design of appointed agencies is likely to have an effect on resulting decisions: institutions always entail biases that benefit some interests more than others and they define standards by which decisions have to be justified. Assuming that decisions of appointed agencies need to reflect social values adequately in order to gain public acceptance (Clarke and Weale, 2012; Littlejohns et al., 2012), the question of whether and how societal values are reflected in the institutional design of agencies and in their justification of distributive decisions gains not only academic, but also practical relevance. In our assessment of social values, institutions and decisions, we seek to assess the congruence between social and institutionalized values in three different countries – France, Germany and the United Kingdom – and explore the likely consequences of the incongruences we find.

6. **Leck, 2016 – Social Return on Investment: Valuing health outcomes or promoting economic values?**

SROI analyses seek to take account of all social, environmental and economic outcomes – the 'triple bottom line' (Elkington, 1997) – and articulate their associated value in monetary terms (Arvidson et al., 2010; Harlock, 2013; Nicholls et al., 2012). A return on investment ratio is calculated, and this is the element that is often highlighted, but SROI is intended to be about more than generating an economic interpretation of value (Arvidson et al., 2013; Nicholls et al., 2012).

7. **Littlejohns, 2019 - National Institute for Health and Care Excellence, social values and healthcare priority setting**

As a way of publicly justifying its decision-making processes, the National Institute for Health and Care Excellence developed a statement of what it termed its 'Social Value Judgements' in 2005.1 This document was updated in 2008, in response to the National Institute for Health and Care Excellence acquiring responsibilities for public health. Both editions focused on value for money as a foundational consideration for ensuring fairness to National Health Service patients in the context of limited resources. As a testimony to Social Value Judgements' importance, the document was cited in the first judicial review of a National Institute for Health and Care Excellence decision: The key principle underlying NICE's approach to appraisals is that the NHS's limited resources should be targeted on those treatments which provide best value for money. The principle is to be found at paragraph 4.1 of NICE's ''Social Value Judgments – principles for the development of NICE Guidelines''. (8 December 2005)2 Although the Social Value Judgements clearly conveyed the National Institute for Health and Care Excellence's commitment to securing value for money, they also recognised the critical importance of other social and ethical values. Reducing health inequalities, preventing discrimination and addressing disabled people's needs figured prominently in both Social Value Judgement documents. So, when the National Institute for Health and Care Excellence announced in November 2018 that it was updating Social Value Judgements, there was considerable interest in whether any changes would ensue – perhaps reflecting the increasing personalisation of medicine or how the National Institute for Health and Care Excellence was adapting to the addition of social care to its remit, within a global environment of healthcare austerity, scepticism of expertise and, of course, Brexit (the UK referendum decision to leave the European Union).3

First, what is lost when substantive ethical and social values, like those endorsed in both prior Social Value Judgements, are no longer explicitly put forward as guiding principles for the National Institute for Health and Care Excellence? Second, what is the impetus for and objective of this dramatic change – could it be heralding a shift in the way the National Institute for Health and Care Excellence thinks or works? Considering the first question, the main casualties of this new approach are likely to be accountability, transparency, consistency, and public, political and professional understanding of the reasons for the National Institute for Health and Care Excellence's decisions. The move away from substantive ethical values towards procedural principles enables the detail of decision-making procedures and the content of recommendations to remain almost entirely unspecified. Patients who are being denied treatment on the basis of the National Institute for Health and Care Excellence's decisions will particularly struggle to make sense of their situation, as will clinicians who

need to explain such decisions to them. Equally, the National Institute for Health and Care Excellence's decisions are left open to a wide range of professional, financial, political and other interests without providing a basis for scrutinising them. For these reasons, the move towards procedural principles sends a worrying signal to other countries who are establishing priority-setting processes for Universal Health Coverage. Universal Health Coverage is about the values and principles that countries could embrace in expanding health benefits for their populations. With the proposed Social Value Judgements, the National Institute for Health and Care Excellence risks losing its status as a leading example from which others might learn how to combine procedural and substantive values in decision-making.

8. **Ong, 2020 – Moral distress, moral equilibrium and the moral equilibrium framework: health professionals' well-being in the face of daily challenges to moral values and integrity** - T**hesis**

The first is the concept of moral equilibrium-disequilibrium that offers a new way of understanding and responding to moral challenges in palliative care. This new concept emerged from the iterative analysis of the experiences of participants in this setting. Moral disequilibrium results when health professionals, faced with challenges to their values and moral integrity, are unable to adapt to maintain their moral integrity. Moral disequilibrium is part of the everyday experience of being a health professional, and like all experiences will vary from person to person and in intensity, from mild (e.g. moral discomfort) to severe (e.g. moral distress, moral injury). The moral equilibrium-disequilibrium concept provides a positive perspective to moral challenges, and also considers the psychological, spiritual and physical wellbeing of health professionals. The concept is of practical value to clinicians as it names the dynamic involved in their experiences without the associations of varied current definitions of moral distress. If moral disequilibrium can be identified and addressed early, it may mitigate the progression into further disequilibrium.

The second significant contribution is the development of the novel Moral Equilibrium Framework (MEF). The framework comprises three dynamic phases: identification of moral disequilibrium and the moral values involved; its resolution in attaining a "good enough" moral equilibrium; and the evolution phase of growth in moral understanding including the building of moral resilience.

Rushton (2016b) defined moral resilience as "the capacity of an individual to sustain or restore their integrity in response to moral complexity, confusion, distress, or setbacks… minimize one's own suffering, and allow one to serve with highest purpose" (p. 112). Re-establishing personal and professional moral integrity is key to managing moral distress. Others have also promoted the building up of moral resilience as a way of dealing with the growing awareness and probable incidence of moral distress (Lützén & Ewalds-Kvist, 2013, p. 320-321; Monteverde, 2014). Rushton offers preliminary suggestions to build moral resilience: the person must know themselves well, especially their values and their personal dynamics including strengths, limitations and "moral core" (Rushton, 2016b, p.116). Amongst what she highlights is the need to develop self-regulatory capacities, encouraging mindfulness and transformational learning, as well as developing ethical competence and engaging with others (Rushton et al., 2013). The theoretical concept of moral resilience seems 31 compelling but has not as yet been substantiated empirically in a significant way as an intervention. However, programs such as "The Mindful Ethical Practice and Resilience Academy" to build moral resilience show promising positive outcomes (Rushton, 2018, pp. 246-247).

…

Secondly, being passionate about the work and buoyed by significant satisfaction in what they were doing aided resilience to challenges. Resilience is defined as “the ability of an entity... to withstand, adapt, recover, or rebound from adversity, stress, or trauma” (Rushton, 2018, p.105). Healthcare professionals like Riva, often found creative coping strategies to counteract the daily stresses inherent in the work (Ablett & Jones, 2007). As Riva remarked, “you cope with it.” Riva alluded to a significant observation—passion and job satisfaction helped build resilience to the stress of work. This resilience included creative coping mechanisms which enabled Riva to continue her passion and job-satisfaction. Thirdly, Riva’s comment of “when you can’t care for patients the way you believe they should be cared for, then you are not quite right,” was revealing. I posit that she was alluding to the challenge to her moral integrity—when she was unable to do what she believed was in accordance with her professional and personal values. The “not quite right” feeling, I argue, was the feeling of disequilibrium. Finally, Riva’s observations in her management role also led her to believe that unaddressed moral distress contributed negatively to general wellbeing: “tiredness, complaints, feeling stressed…. You’re finding fault in things you normally can handle.”

9. **Taylor, 2002 - Professional Dissonance: Colliding Values and Job Tasks in Mental Health Practice**

Victor Frankl (1988) has termed a ‘‘value collision.’’ For example, the current mental health practice context increasingly requires mental health practitioners to engage in involuntary treatment interventions leading to a practice climate rife with potential value collisions. Such potential value collisions can include: providing mandated or involuntary treatment, intervening in a consumer’s decision-making about medication, breaching confidentiality to communicate with family members, and making decisions about a consumer’s ability to direct his or her own life, such as advocating for outpatient commitment, or court orders allowing forced medication.

Professional dissonance is conceptualized as a feeling of discomfort arising from the conflict between professional values and expected or required job tasks. The conceptualization of professional dissonance as a researchable topic for practice, as well as the generation of the study’s dissonance-reducing cognitions, owes a large debt to Leon Festinger and colleagues’ previous work on cognitive dissonance (Festinger, 1962, 1964; Festinger & Carlsmith 1959; Harmon-Jones & Mills, 1999).

Existential theory, especially, speaks to the internal, spiritual struggle to live an authentic life and is therefore instructive in developing professional dissonance as a problem for social workers.

The core ideas of existential theory are important to the present research because the key existential issues of ‘‘authenticity’’ vs. ‘‘bad faith’’ and ‘‘ontological guilt’’ vs. ‘‘ontological anxiety,’’ speak to the current struggle in the mental health practice context to negotiate a practice position that simultaneously protects a consumer’s rights (even to fail) and ensures the practitioners’ ability to make professional, caring and sometimes unpopular decisions about what should happen in an intervention. The discussion of professional dissonance highlights the notion of the dissonance process as one filled with confusion, angst and, hopefully in the end, professional and personal growth.

Existential psychology, with its focus on authenticity and responsibility, speaks directly to the cost of bad-faith actions. In this way, it answers the question of what happens to practitioners who consistently act in a way that conflicts with their ideas of what they should be doing or as Margolin (1997) calls it, living in contradiction. It also changes the negative cast of anxiety to encompass anxiety as a potential growing experience. In this way, existential psychology balances the prevailing view of anxiety as burn-out or pathological, by casting it as a growing pain. This recasting is appropriate for the current research study, which combined the views of anxiety as problematic (as seen in cognitive psychology and ego psychology) with the idea of anxiety as a possible vehicle to excellent practice in the crafting of the concept of professional dissonance.

The current research focused a great deal on the experience of conflict, ambivalence and anxiety among social workers when trying to decide what is the next right thing in practice interventions amid the ‘‘gray areas’’ of mental health. One of the major contributions of existential theory, however, is to recast anxiety from a negative hobgoblin of burn-out and attrition in practice into a state of potentiality. This spirit of ‘‘becoming’’ can enliven the concept of professional dissonance in a way that moves it from a pathological problem needing a cure to a practice process that signals avenues of change. Along this line, respondents affirmed their willingness to ‘‘sit with’’ dilemmas in order to work them through. One reported seeing dilemmas as ‘‘A golden opportunity to review what is really important.’’ Another agreed, ‘‘Life is full of conflicts. Things are not simple.’’ The practice wisdom of the sample population was evident in this area. Respondents also spoke to the importance of simply doing the best they could as practitioners: ‘‘Make a decision, move on, let go,’’ ‘‘Pray I’m doing the right thing.’’ It would seem that perhaps the idea of ‘‘good enough practice’’ especially when partnering with consumers has potential for mental health practice and may go a long way in helping to prevent burn-out from unresolved professional dissonance. This dimension of living with dissonance and doing the best you can has implications for education of future mental health practitioners to help them learn to expect dissonance and dilemmas and normalize the process while underlining the importance of life-long supervision and collegial support.

10. **Velez, 2020 - A framework for explaining the role of values in health policy decision-making in Latin America: a critical interpretive synthesis**

Values, defined as “principles, or criteria, for selecting what are good (or better, or best) among objects, actions, ways of life, and social and political institutions and structures” [4], are essential at all stages of the policy process, ranging from the prioritisation of some issues over others on a government’s agenda to the development of policy options to address an issue and the implementation of selected policy options. When governments or institutions more generally set agendas and develop and implement policies, they also legitimise and promote certain values over others, making value-laden decisions about health systems [5].

We identified 116 values in the 207 papers included (see Supplementary material 5 for the list and frequency of values identified). We found from this that stakeholders and policy-makers in Latin America call a great variety of things ‘values’ in their writings, including the right to health, equity, universality, sustainability, decentralisation, feasibility, privatisation, primary healthcare, Millennium Development Goals, and many more. Further, these values describe concepts that are quite

different from each other, such as principles, strategies, instruments, specific goals, elements of a policy, or beliefs about the health system.

We propose that the values considered in the policy process can be characterised in four ways, namely (1) goal-related values (i.e. guiding principles of the health system); (2) technical values (those incorporated into the instruments adopted by policy-makers to ensure a sustainable and efficient health system); (3) governance values (those applied in the policy process to ensure a transparent and accountable process of decision-making); and (4) situational values (a broad category of values that represent competing strategies to make decisions in the health systems). This theoretical framework is represented in Fig. 4, which can be thought of as a heuristic that can be used to identify the four categories of values and the conditions in which values are used in different stages of the policy process.

The seriousness of health policy decision-making requires that the procedures for making decisions reflect values like accountability, social participation, stewardship and transparency. Governance values are not final goals of the health system but promote the principle of legitimacy in health policy development and implementation.

Situational values are values that become important in specific circumstances. These values reflect policy legacies, changes in the balance of organised forces, interests of influential groups, ideological positions, changes in the national mood or international influences. We propose that situational values are not the ultimate goal of the health system. However, some situational values become tremendously important for a country at particular points in time and governments could incorporate them into technical or governance categories or even misrepresent their role and feverously pursue them as though they were a goal of the health system. For example, at the end of the 80s and 90s, virtually all countries in Latin America began the process of reforming their health systems and pursued values promoted by the World Bank and the Inter-American Development Bank, including privatisation, competitiveness and market [20, 25, 28, 29, 45, 57, 85]. Reformers maintained that privatisation would improve other high standard values like accessibility, efficiency, equity, quality and social participation [35, 30, 47, 59, 81, 86, 87].

This situational values category is complex because different competing values belong here and there is no consensus about the legitimacy of those values. For example, some governments highlight decentralisation and others pursue centralisation; some countries promulgate compulsoriness and others ask for voluntariness; and some endorse public financing whereas others prefer privatisation. Countries that implemented private health insurance models commonly asserted competitiveness, privatisation, market, targeting, cost-containment and efficiency as the most appropriate mechanisms to achieve universality in a liberal, market-oriented society [19–21, 25, 28–30]. Those values are identified in the health system reforms of Brazil [21, 31–33], Chile [29, 32], Colombia [21, 32–34], Costa Rica [35] and Mexico [20, 28, 36, 37]. However, when we examined the strategies followed by more public financing-oriented governments, other values like public financing, primary healthcare and centralisation appeared (e.g. Bolivia, Cuba, Ecuador, Venezuela) [26, 81–84]

Agenda-setting Values are used to frame problems in health systems and to prioritise issues on government agendas; they also shape how some issues gain prominence in the government agenda

given that this is a precursor for identifying policy options. When governments compare their indicators based on the goals they hope to achieve with similar indicators or expectations from other countries, such comparisons are important factors in framing an issue as one that warrants a government's attention when those comparisons result in negatively framed goal-related values (e.g. lack of universality/solidarity, inequity, bad quality or vulnerability to the right to health) [23, 25, 27, 28, 47–50]. Governments also pay attention to problems that are framed in relation to inefficiency or as threats to the fiscal sustainability of health systems [22–25, 28, 38, 62, 63] and, more recently, to problems regarding corruption, lack of social participation or deficiencies in accountability.

CMOC 3 – Powerless and detached

Version 2

In order to achieve efficiencies - information systems for example were introduced to track activity and spending, health professionals perceive a loss of autonomy and decision-making power, leading to a sense of powerlessness and detachment and ultimately a resistance to change and conflict between front line workers and policy decision makers / management.

Search Summary: Title words: autonomy OR empowerment AND healthcare professional OR doctor OR nurse = 17 (12 potentially relevant, 4 full texts available)

11. **Cleary, 2016 - The Limits of Professional Autonomy: An Interview-based Comparative Analysis of the Workplaces and Perceptions of Educators and Healthcare Professionals - Dissertation**

The "modern bureaucracy" has been sociologically relevant since Max Weber and Frederick Taylor separately wrote about scientific rationalism and bureaucratic influence in the early 20th century72. Applications of bureaucracy research have been diverse, ranging from the professions (such as public education and healthcare) to the bureaucratization of the mafia (Haller 1992). A good deal of sociological research in the late 20th century and early 21st century has concerned itself with making bureaucratically structured organizations more bureaucratic (more efficiently managed). Structural-functionalist perspectives dominate this research. There is also a considerable body of literature (though not quite as much) that is phenomenonological, and attempts to understand how individuals experience phenomena in their workplaces. Such literature is generally ethnographic and symbolic interactionist in nature and design and usually focuses more on the experiences of employees than the experiences of employers.

The participants in this study reveal professional autonomy to be a multi-layered phenomenon. Professional autonomy can also be a social construction and a shared experience. Landahl Hospital doctors illustrated this idea, with prideful and fearful responses about their beloved workplace culture; Bridgton's teachers similarly demonstrated a collective sense of pride in their very autonomous workplace.

An individual's sense of professional autonomy is a product of the individual's professional environment, specifically, the external and internal bodies that respectively fund and 72 Scholarly interest on bureaucracies likely predates Taylor (1914). The purpose of this statement, and this

section, is to provide a brief history of sociological literature on bureaucracies, and to establish a beginning point of my sociological analysis on bureaucracies. I am not attempting to provide an exhaustive historical account of scholarly interest on bureaucracies. 123 manage the individual's workplace. More importantly, each workplace is structured according to the needs of the external funder at a particular historical moment: Autonomy in public education is presently a function of public sentiment (i.e. measures of student academic performance and the resulting effects on the politicians and educational bureaucrats whose jobs depend on student performances); hospitals are affected by monolithic pressures, coming from payers, to constantly cut their costs. Individual public schools win professional autonomy for their members (teachers and principal) vis-à-vis superior student academic outcomes. Hospitals earn professional autonomy for doctors and executives by outcompeting (and "out-conforming") rival hospitals in the high-stakes game of cost minimization. Individuals and organizations within each field that deliver external funders that which they desire (political goodwill or lowest costs) are declared "winners" by their respective external bodies. Their "prize" is professional autonomy (those who fail to win do not get as much of the prize).

A common sentiment expressed in discussions about public education and healthcare in America, by practitioners and the popular public at large, is the idea that any argument or opinion about either institution is best advanced by explaining how said argument or opinion leads to "better outcomes" for students and/or patients. This pervasive and unexamined presumption has infiltrated American discourse. Turn on the TV and listen to a discussion on CNN (or FOX, MSNBC, CNBC, PBS, etc., etc.) about public education in America, and you are likely to hear any number of different ideas on how "we" should reform our schools in ways that maximize "student achievement" (at the moment, charter schools and "school choice" seem to be garnering the most headlines). Attend a parent teacher organization meeting, and you are likely to hear similar, redundant (though passionate) protestations of "doing what's best for the kids." You are just as likely to hear this sentiment in a TV program as you are to read it in scholarship on public education: If it leads to "better outcomes" and "higher achievement" for students, then it is a good idea or argument. Similarly, popular and academic consciousnesses assume that any healthcare reform that is worth talking or writing about must be rooted in patients' interests. Such discourse belies what should be intuitively recognizable: the people in these professions are not sacrificial lambs; they are like any adult who possesses their own, self-oriented needs. But this point struggles to see the light of day in America because it is deemed selfish and un-noble.

Sociological literature on bureaucracies reflects and indeed strengthens this popularly held sentiment. This is particularly true of structural-functionalist works like the ones that were summarized in this dissertation. Symbolic-interactionist works are not immune either. Like the layperson, this research assumes bureaucracies to be highly instrumental entities that can be manipulated in order to achieve particular outcomes. As iterated throughout this dissertation, such thinking pursues general goals of efficiency and profit maximization; specific goals include "reforming America's public schools" and "revolutionizing the way healthcare is delivered in the United States." Weber (2010) argued that bureaucracies were the most powerful invention to ever seek and attain humanity's economic and social goals. However, the problem with the popularly and scholarly held sentiment (that the purpose of a bureaucracy is the attainment of goals in service of others) is that it ignores a significant component of them: employers' and employees' needs and wants. Sociologists of work who employ a symbolic-interactionist framework, such as Gary Alan Fine, tend to focus exclusively on employees but not employers.

Employees interviewed for this dissertation were sympathetic to the needs of their employers. Their pursuit of emotional fulfillment did not prevent their awareness of and support for the employer's goals. Despite incredible frustration and burnout, Principal Eric Smith is a "team player" who follows the orders of the Louisiana State Department of Education and the 125 local school board. "…I mean I understand, well, if they're giving us the money, then they ought to be able to tell us [what to do]…" (Smith). And, despite strongly resenting the way in which the recent merger was handled, the doctors from Landahl Hospital's Department of Internal Medicine believe that merging with another hospital was necessary for financial survival (even though many might wish it had not been with Stewart Hospital). Although their primary sense of fulfillment and identity is associated with patient care, they believe not only that a hospital has to have its business affairs in order, but that a good doctor accepts this reality. A Landahl doctor embodies their measured acceptance of business-related needs, "… [O]ur residents are much more aware of the business of medicine than I was… when I was a trainee 20 years ago now… I think that that's actually a good thing" (M. Kepler).

Throughout all three studies, I found interviewees to possess a similar mindset that balanced the realities of employed work with their own personal needs of fulfillment. Each interviewee expressed some professional desire, and their descriptions of their professional desire informed their experiences with, and their conception of, "professional autonomy."

The interviewees did not strike me as radicals, deviants, or even "odd." They are mainstream, everyday Americans. I found many to be compassionate, empathetic, and intelligent. Yet not once during the course of my research did any interviewee support their claims by referencing "the good of the students" or "the good of the patient." They spoke of professional autonomy as if it is necessary to the performance of their work. They believe they do their best work when they possess sufficient autonomy.

12. Laschinger, 2003 - Organizational Trust and Empowerment in Restructured Healthcare Settings: Effects on Staff Nurse Commitment

Cook and Wall [1] defined organizational trust as the extent to which one is willing to ascribe good intentions to, and have confidence in, the words and actions of other people. Trust has a significant impact on important organizational factors such as group cohesion,[2] perceived fairness of decisions,[3] organizational citizenship behavior,[4] job satisfaction,[1,2,5] and organizational effectiveness.[6,7] Mistrust results when information is withheld, resources are allocated inconsistently, and employees have no support from management.

Without trust, people cannot or will not work together except under conditions of stringent control.[8] Ironically, at a time when trust is most needed for successful organizational transformation, the changes resulting from restructuring have diminished trust in the work setting. This state of affairs has serious implications for organizational performance.[9] Nurses, the largest group of healthcare providers in hospitals, have been particularly hard-hit by recent downsizing. It is possible that their mistrust of the system could potentially threaten the quality of patient care.

Kanter [10,11] maintains that work environments that provide access to information, resources, support, and the opportunity to learn and develop are empowering and enable employees to accomplish their work. As a result, employees sense that management can be trusted to do

whatever is necessary to ensure that high-quality outcomes are achieved. According to Kanter, employees in environments such as these are more committed to the organization and more likely to engage in positive organizational activities.

Kanter's theory provides an explanatory framework for investigating the role of organizational trust with regard to factors influencing nurses' ability to function effectively in today's dramatically restructured healthcare settings. The purpose of this study was to test a model linking staff nurses' workplace empowerment, organizational trust, and organizational commitment.

Kanter's Theory of Organizational Empowerment

Kanter 10,11 argues that people react rationally to the situations in which they find themselves. When situations are structured in such a way that employees feel empowered, the organization is likely to benefit both in terms of the attitudes of employees and the organization's effectiveness. In fact, Kanter argues that the impact of organizational structures on organizational behavior is far greater than the impact of employees' personality predispositions.

The organizational structures that Kanter believes are particularly important to the growth of empowerment are having access to information, receiving support, having access to resources necessary to do the job, and having the opportunity to learn and grow. Access to these empowering structures is promoted by formal job characteristics-that is, jobs that are visible and central to the organization's goals and that allow the employee flexibility enhance empowerment. In addition, informal job characteristics such as alliances with superiors, peers, and subordinates in the organization further influence empowerment.

According to Kanter, the mandate of management is to create conditions for work effectiveness by ensuring that employees have access to the information, support, and resources necessary to accomplish their work and that they are provided ongoing opportunities for employee development. Having access to these structures results in increased levels of organizational commitment, feelings of autonomy, and self-efficacy. Consequently, employees are more productive and effective in meeting organizational goals. Relations among the constructs in Kanter's theory are presented in Figure 1.

Numerous studies have linked Kanter's concept of empowerment to important organizational outcomes, such as organizational commitment,12-14 job autonomy,15 participation in organizational decision making,16 job satisfaction,17 perceived control over nursing practice,18 and lower levels of job burnout.19,20 In support of Kanter's belief that powerful bosses tend to empower their staff, Laschinger and Shamian 21 found that nurses who considered their managers to be powerful in the organization also rated themselves high in job-related power. More recently, Laschinger et al.22 found that staff nurses who were more empowered in their work setting rated their leaders higher on the use of leader empowering behaviors, such as encouraging autonomy, promoting participative decision making, and expressing confidence in employee competence.

Organizational trust was defined by Gilbert and Tang 24 as the belief that an employer will be straightforward and follow through on commitments. Trust refers to employees' faith in

organizational leaders and the belief that ultimately organizational actions will benefit employees. Mishra and Morrisey [7] argued that open communication, sharing of critical information, sharing of perceptions and feelings, and greater worker involvement in decisions enhance trust in organizations. Butler [25] identified 11 conditions of trust: discretion, availability, competence, consistency, fairness, integrity, loyalty, openness, overall trust, promise fulfillment, and receptivity.

Trust must be an integral and coherent part of the organizational culture if change is to be implemented effectively and sustained. According to Johns,[26] empowering employees involves understanding their needs and capabilities, trusting them, and helping them to maximize their fulfillment while pursuing corporate goals. Mutual trust is a critical component of this process. Managers must be willing to empower employees, and employees must accept the challenge inherent in empowerment and make a commitment to organizational goals.[27] High levels of organizational trust are needed to accomplish change, but paradoxically the change itself may destroy trust and threaten organizational effectiveness. Research on organizational downsizing has shown that decreased levels of trust are associated with decreased communication and increased conflict.[28] As hospitals continue to downsize, employee trust and morale are eroded as workloads increase and job insecurity escalates.[29] In such low-trust organizations, behaviors such as high absenteeism, prolonged breaks, limited learning, low accountability, reactionary thinking, and low creativity are predictably common.[30]

Organizational commitment consists of employees' attachments to their organization.[38-40] According to Meyer and Allen,[41] there are three types of organizational commitment. *Affective commitment* is a person's emotional attachment to, identification with, and involvement in a particular organization. Employees with strong affective commitment work in the organization because they want to. *Continuance commitment* reflects an employee's awareness of the costs associated with leaving an organization. People with high continuance commitment believe the benefits of staying with an organization outweigh the consequences of leaving and stay with the organization because they need to. This type of commitment is likely to be prevalent in today's downsized work environments. *Normative commitment* reflects a person's sense of obligation for remaining in the organization.

Affective commitment has many positive consequences for the organization. It has been found to be positively related to job satisfaction, job involvement, job performance, and organizational citizenship behavior.[42] Employees with strong affective commitment contribute more to the accomplishment of organizational goals and are less likely to leave the organization. Although employees with high continuance commitment are also less likely to leave the organization, this lower turnover occurs at the expense of employee engagement, job satisfaction, and self-esteem.[43] Employees with high continuance commitment may be motivated to do the minimal amount of work required to maintain their jobs. Thus, this type of commitment may be counterproductive to the accomplishment of organizational goals and objectives.[43,44] This claim was corroborated by McCloskey and McCain,[45] who found that nurses who stayed in their positions because of job scarcity reported higher levels of absenteeism and were increasingly more likely to demonstrate poor work performance.

Organizational commitment is of particular importance to healthcare organizations. Employees in these turbulent environments are struggling to maintain high-quality patient care with fewer resources. The empirical evidence suggests that employees with high affective commitment are

more likely to rise to the challenges imposed by restructuring, but employees with high continuance commitment may simply do the minimum. Moreover, Glisson and Durick 16 found that employees displaying higher levels of affective commitment were more resistant to job strain and burnout, suggesting that affective commitment may help employees withstand the negative effects of downsizing. Clearly, it is important for healthcare organizations to promote the factors that encourage affective commitment and to reduce those that encourage continuance commitment.

Meyer et al.47 suggest that work experiences are the strongest predictors of affective commitment. In a meta-analysis of organizational commitment research, Mathieu and Zajac 48 found that job scope, challenge, and high levels of work autonomy were consistently related to commitment. Bateman and Strasser 49 found higher levels of affective commitment when exceptional job performance was recognized and rewarded. Opportunity for advancement, perceptions of fairness regarding the distribution of rewards, and workplace autonomy were found to enhance staff nurses' organizational commitment.50

The results of this study support the proposition that staff nurses' empowerment affects their trust in management and ultimately influences affective commitment. More specifically, staff nurses in this study believed that their sense of workplace empowerment strongly affected their trust in management and subsequently their belief and acceptance of organizational goals and values, their willingness to exert effort in the workplace, and their desire to stay in the organization (affective commitment). These findings highlight the importance of creating environments that provide access to structures that empower nurses to accomplish their work.

The strongest relationships were found between trust in management and nurses' perceived access to information and support. The relationship between access to information and trust is consistent with Hart et al.'s 23 findings linking trust to the willingness of managers to share accurate information in a timely fashion. When this does not happen, the perception may develop that information is being hidden. At this point, the grapevine takes over. As speculation grows as to why information is not provided, feelings of safety and comfort in the manager-staff relationship deteriorate, and trust in management is destroyed. Similarly, the fact that access to support and trust were strongly related suggests that when staff nurses are provided with helpful feedback and guidance from managers and given the flexibility to use their judgment and make discretionary decisions, their trust in management increases. Assuming responsibility for these actions entails a risk on the part of staff nurses. Because trust is defined as the willingness to take risks and to be vulnerable to the action of others based on the assumption that the other will act in a manner beneficial to the trustor,60 support from managers that leads to successful decision making or damage control for mistakes benefits the trustor and fosters trust in management.

The relationships between trust in management and access to resources and opportunity were lower but still significant. According to Gambetta,61 trusting another person is based on the assumption that the other will act in a manner that is beneficial to the trustor, and this assumption leads to a willingness to engage in a cooperative effort. Staff nurses and their managers must cooperate to provide care for patients. Staff nurses are responsible for providing expertise in patient care, and managers are responsible for furnishing the resources needed for this care. If

managers cannot provide these resources as a result of staff shortages, fiscal restraint, breakdown of aging equipment, or inadequate supplies, staff nurses may not be able to keep their side of the agreement.

A weaker relationship was found between trust in management and access to opportunities to learn and develop. This is not surprising. With the current cutbacks in hospital funding, educational opportunities for staff have been slashed. For the few that remain, the increased workload makes it virtually impossible for nurses to leave their units to attend classes. Ultimately, this may lead to negative patient care outcomes, because nurses may not be able to upgrade their knowledge through continuing education.

13. **Traynor, 2010 - Professional autonomy in 21st century healthcare: Nurses' accounts of clinical decision-making**

Examining the French university hospital system in the 1960s, Jamous and Peloille proposed that the decision-making of professionals differed to that of other occupations. Because professional work concerns situations that are high in indeterminacy professionals employ higher degrees of tacit judgement than those in non-professional occupations. They argued that the decision-making carried out by members of other occupational groups was based on relatively more easy to formalise, technical factors. They predicted that a study of how these two types of decision-making affected work would empirically identify 'genuinely' professional occupations because professional work would feature a high proportion of indeterminacy relative to technicality e their so-called 'I/T ratio'. Their work has been drawn on in recent studies of the medical and nursing professions though their ideas have been applied more to studies of professional rhetoric than decision-making in practice (Atkinson et al., 1977; Traynor, 2009). Looking at professional rhetoric it is possible to argue that groups like medicine or nursing face a dilemma. If they account for their practice too strongly in terms of its technical complexity, explicit rules and procedures (that could be set out in a manual for example) this risks the possibility of intervention and control by outside groups such as bureaucrats and managers because of the predictability and visibility of the work. To avoid this, professions may call attention to the indeterminacy of their work. Indeterminacy would call for professional judgement, or the use of tacit or private knowledge. It therefore allows professions to emphasise the social qualities and experiences of their members, which qualify them to make such judgements. The risk of too heavy an emphasis on indeterminacy is that other groups can claim equal or superior skill and the indeterminist loses control over their field and the ability to make rational decisions within it. So the most effective professional claims would feature a particular combination of both.

14. **Tyan, 2010 - Understanding Taiwanese home healthcare nurse managers' empowerment and international learning experiences: Community-based participatory research**

The participants' accounts of their empowered and powerless experiences within the home healthcare context were grouped as attributes of empowerment, attributes of powerlessness, and factors affecting both. The findings indicated that the nurse managers felt empowered on the individual and interpersonal levels but powerless on the system level.

Empowerment is an important concept that significantly contributes to professional development and quality of care (Corbally et al., 2007; DuPlat-Jones, 1999; Gibson, 1991). Research has shown that empowerment is positively linked to job satisfaction and productivity among nurses (Kuokkanen

et al., 2002; Manojlovich, 2007). In contrast, nurses who describe themselves as powerless are less satisfied with their jobs and contribute to poorer patient outcomes (Manojlovich & Laschinger, 2002; Manojlovich & DeCicco, 2007).

Nurse managers play a crucial role in empowering nurses in the field as well as assuring quality of patient care (Cameron-Buccheir & Ogier, 1994; Laschinger et al., 2007). Nurse managers who feel empowered are more likely to fulfill their managerial roles (Patrick & Laschinger, 2006; Upenieks, 2003), and their subordinate staff have lower turnover and higher morale than disempowered nurse managers (Corbally et al., 2007; Hayes et al., 2006). Therefore, empowering nurse managers is regarded as an important organizational strategy to promote nurses' clinical effectiveness and improve patient outcomes (Flynn, 2005; Laschinger et al., 2007; Lee & Cummings, 2008).

Categories and Subcategories of Empowerment and Powerlessness

Categories	Participants
**Attributes of Empowerment**	
Independent competence in practice	N1~N4, S2~S5 (n=8)
Professionalism being recognized	N1, N2, N4, S1~S5 (n=8)
Continually growing in the profession	N1, N2, N4, S1, S2, S4 (n=6)
Having hope to advance HHC	N1, N4, S1, S3~S5 (n=6)
**Attributes of Powerlessness**	
HHC not being recognized	N1~N4, S2, S4, S5 (n=7)
HHC being subordinate within the healthcare system and within participants' organizations	N1, N3~N5, S1, S2, S4 (n=7)
Not being able to expand HHC service	N1, N4, N5, S1, S2, S4, S5 (n=7)
No standard for HHC quality	N1, N3, S1, S4 (n=4)
**Factors Affecting Empowerment or Powerlessness**	
Social/healthcare system change	N3, N5, S2, S4 / N1, S1, S5 (n=7)
NHI HHC reimbursement levels	N1~N5, S1~S5 (n=10)
Organizational structures/resources/supports	N1~N6, S1~S5 (n=11)
Nurse – physician relationship	N1~N5, S1~S5 (n=10)
Nurse – patient/family relationship	N1~N4, S2~S5 (n=8)

CMOC 4 – Working the system (access)

Version 2

A sense of professional / moral duty or ethical decision making, solidarity with patients or fellow health professionals led to health professionals circumventing policy to deliver care (legal, informal referrals, treat regardless of ability to pay), but ultimately lead to strain on frontline workers, increases ER use but more stable health outcomes than originally predicted.

Search Summary

Search 1 – Title: street level bureaucrats AND health = 16 (Keyword resilience = 3)

Search 2 – Title: moral distress AND health Keyword: resilience = 34 (resilience in title = 2)

Search 2.1 – Title: moral distress AND health Keyword: austerity = 6

Search 3 – Title: "ethical decision" AND health AND moral = 6

17 in total, 15 potentially relevant, 9 with full texts

15. **Lee, 2014 - My moral compass and ethical decision making in public health**

If we have learned one thing in the course of the development of the field of public health ethics over the past quarter century, it is that my moral compass might not be in complete agreement with my colleague's; my true north might not point to his true north. Reasonable people disagree. While we might not be so far from each other on what we think the right thing is to do, the distance might be of vital import. How, then, do we get to an ethical decision about what to do next? Simply stated (albeit less simply implemented), we turn from a moral compass to a process of ethical decision making. Ethical decision making is a process by which questions with less than clear ethical solutions are posed before an established framework steeped in a common set of principles that guide the decision makers through evidence, ethical considerations, and scenario shifts to arrive at one of several possible paths forward. Often the chosen path is not what each individual's moral compass would dictate, rather, given that each person has agreed with the common set of ethical principles guiding the ethical framework, the resulting decision path is one (perhaps of several) that follows from systematic ethical evaluation. It is the achievement of this result that places ethical decision making far beyond that of even the most virtuous person's moral compass. In addition to the value of incorporating systematic ethical evaluation into the everyday practice of public health professionals, there are at least three additional compelling arguments in favour of relinquishing our personal moral compass for a more robust decision making framework in public health. In public health we must account for 1) the primacy of transparency in deliberating the ethical contours of public health policy decisions; 2) the extant pluralism in the affected community and the population as a whole; and 3) the fact that communities and populations, not solely individual patients, are the focus of the ethical duty in our practice.

Transparency

Participation and deliberation, in the context of ethical decision making with a framework of mutually valued and important principles, functions as a tool for improved outcomes, legitimized decisions, accountability, and buy-in on decisions even when the outcome is not what individual participants would have desired. These outcomes stem from trust in a transparent process and agreement with underlying principles. Gutmann and Thompson describe democratic deliberation as

a powerful tool for decision making in bioethics and in the political process between citizens and their elected officials.

Pluralism

Different stakeholders might have different opinions on the weight of each of these values for a given ethical decision at a given time or given the current state of scientific evidence, and these are the issues that are deliberated. It is the use of such a framework, with mutually agreed upon values and principles, that allows public health practitioners to move from applying their personal moral compass toward an ethical decision making framework that considers the plurality of the community and allows application of a transparent, defensible process.

Focus on community

When dealing with a public good, moral governance shifts from the individual to the collective and ethical decision making is based on common values, articulated as principles, that engages all affected parties. One strength of this process is its reliance on a collective framework, not a personal moral compass even if the outcome would have been identical.

16. **Edwards, 2011 - Moral agency as enacted justice: a clinical and ethical decision-making framework for responding to health inequities and social injustice**

Moral agency refers to the realization of a capacity (as an individual or group) to act morally and for change in a situation…

Justifying Decisions in Clinical Practice…

Reflective Equilibrium Reflective equilibrium is a method, initially described by Rawls," that is used in ethical decision making to reflect on a perspective or judgment in order to reach a justified moral position between competing moral judgments. In reflective equilibrium: . . . we "test" various parts of our system of beliefs against the other beliefs we hold, looking for ways in which some of these beliefs support others, seeking coherence among the 'widest set of beliefs, and revising and refining them at all levels when challenges to some arise from others .. . a person who holds a principle or judgment in reflective equilibrium with other relevant beliefs can be said to be justified in believing that principle or judgment.'' Reflective equilibrium may be used in various circumstances and would be evoked (either spontaneously or intentionally) if a person believed that the killing of innocent people was morally wrong but was put in a position to have to make a judgment on the death of innocents

A more: mundane but perhaps relevant example of reflective equilibrium in the context of resource distribution in health was provided by Daniels; In thinking about the course of right action in a particular case, we often appeal to reasons and principles that are notoriously general and lack the kind of specificity that make them suitable to govern the case at hand without committing us to implications we cannot accept in other contexts. This requires that we refine or specify the reasons and principles if we are to provide appropriate justifications for what we do and appropriate guidance for related cases. Suppose, for example, that we are considering whether we should ignore age in the distribution of medical treatments. Many initially believe that age is a "morally irrelevant trait," just like race, and they would insist that rationing medical services by age is just as unacceptable as rationing by nice. On considering a variety of cases, however, it might become

apparent that we all age, although we do not change race. This difference means that the different treatment of people at different ages, if systematically applied over the lifespan, does not create inequalities between persons, as it would in the case of race. We might be led by this realization to think that age rationing might be acceptable under some conditions when race rationing would never be. and this would be a moral surprise for many who changed their view. It has been suggested that the success of reflective equilibrium rests on a presupposition that "we are justified to take our present beliefs (the present provisional equilibrium) as a reliable point of departure of reflection and justification. Otherwise, a person or group could reach an "ethical coherence" among various beliefs and judgments that they consider relevant but that might still represent an immoral or distorted position in a wider context." Informed by his understanding of the social determinants of health, Daniels defined a wide reflective equilibrium in which; . . . we broaden the field of relevant moral and non-moral beliefs to include an account of the conditions under which it would be fair for reasonable people to choose among competing principles.'"' This is an important addition, as it implies the need to extend moral deliberation beyond individual experience, or what is in our "own heads" (including personal and professional value systems), and to develop an analytical reasoning process that engages with the beliefs of others from the perspective of their contexts (or conditions).

Reflective Equilibrium and the Ethical Reasoning Bridge… originally expressed by Hunter, who described the inquiry task of the medical practitioner as crossing a bridge where, on one side, information about a case that is universal and shared by all or most patients is sought and, on the other, information that is unique to a particular patient is sought.2' The ER bridge embodies a form of wide reflective equilibrium.

The ER Bridge and Epistemology…

The ER Bridge and Dialectical Reasoning…

Facilitating Transformative Learning in Clinical Practice

The story of the health care workers of Project Prójimo and their therapeutic and moral learning, from the meeting of rehabilitation needs of individual villagers to the empowering of a local community, manifests moral agency. The evolving sense of "agency" gained by these health care workers and their community with respect to ameliorating the disempowering effects of disability can be traced, as a form of learning, with each crossing of the ER bridge. The learning that these village health care workers embodied was described by Freiré as ""conscientization."" Freire's account of knowledge and truth can be related to a critical research paradigm, the underlying assumptions of which include those previovisly described and a historical perspective in the experience of power (or powerlessness). In Freire's critical pedagogy, the purpose of learning, as in conscientiztion, is for the learner (individual or group) to become aware of the factors and conditions, including historical influences and effects of power structures, that constrain one's choices and actions in a particular context and weaken or even extinguish a sense of moral agency. "^^ The objective of such leaming is transformative learning and emancipatory change in which both health care workers and patients are involved." The kind of learning for understanding and change described above is not only redolent of Wildes' call for a "wider phenomenology" as a more inclusive moral basis for health care. It is also an embodiment of justice, enacted through enhanced moral agency in partnership with patients and their communities.

Moral Agency: An Individual Practitioner and Collective Professional Enterprise Although it is practitioners who encounter situations of inequity and injustice and who try to both make sense of and resolve them at the "coal face" of clinical practice. We acknowledge that these inequities and injustices ultimately will need to be addressed at institutional and regulatory levels. It is important to state that although the capability approach focuses on justice concerns pertaining to the actual lives of individuals, it does so in the context of the wider social conditions and determinants that we have discussed.'^ Thus, there is a need for both individual and public forms of moral reasoning. "That is, there is a need for scrutiny of what one person interprets as an injustice to be set in the context of community or population discourse regarding what is unjust or not.^ Issues of inequity or injustice that were encountered in the assessment and treatment of individuals at Project Prójimo became issues that were gradually shared and discussed by the community as a form of public reasoning. Public discourse concerning the forging of consensus around common concerns and experiences is a commonplace in all kinds of communities (including professional). In our final reference to the Figure, the relationship between a practitioner and his or her community of practice and the relationship between a patient and his or her community context is illustrated. The practitioner ideally embodies the values, knowledge, and obligations (expressed in the code of conduct) of the profession while contributing to and building those same values, knowledge bases, and commitments. ^^ What the practitioner learns in the course of physical therapist practice is "returned" in discourse to colleagues and ultimately to the collective association.' For example, clinical or professional issues and concerns, reporting of experiences derived from unusual or complex cases, and letters to the editor are all common sights in professional journals and provide for a wider discourse between practitioner and profession. On the right-hand side of the bridge, the patient, too, has a relationship with the community. The patient's health status and quality of life, in part, may be representative or expressive of the health of that community (or other population).'»ss Yet patients are also people with potential agency who are able to contribute to change in and with their community. The emergence of consumer- and patient-led groups with emphases on improved provision of health information, self-management skills and strategies, and patient advocacy exemplifies this relationship.56 57 It is the extent to which health care practitioners have a sense of their own moral agency (and that of their profession) that, in turn, allows them to facilitate the agency and capabilities of their patients in and with their communities. Enacting justice as a practitioner at a clinically based level becomes part of a continuum by which professional associations of physical therapy are informed in their representations to government and institutional authorities in the course of the development and reform of health policy. It is in enacting justice throughout the whole of this practitioner-association continuum that our responsibility in fulfilling the social contract as a profession is fulfilled.

17. **Khokhar, 2021 - Stress, Resilience and Moral Distress among Health Care Providers During COVID-19 Pandemic**

Moral distress is the painful psychological disequilibrium that results from recognizing the ethically appropriate action, yet not taking it, because of such obstacles as lack of time, supervisory reluctance, an inhibiting medical power structure, institution policy, or legal considerations.

18. **Splig, 2022 - The new frontline: exploring the links between moral distress, moral resilience and mental health in healthcare workers during the COVID-19 pandemic**

The COVID-19 pandemic has had major collateral effects on the global healthcare system creating sustained and unrelenting pressure to re-allocate scarce healthcare resources including HCWs. During the pandemic, many HCWs have encountered potentially morally distressing events (PMDEs) over and above the stressors faced during their typical practice, such as risk of COVID-19 transmission to family members, caring for patients without family members present, triaging patients in the context of limited resources where the lack of treatment may result in death, and following directions that go against their professional standards or core values [7, 8].

…

Concerns have been raised about the risks associated with moral distress and moral injury faced by HCWs during the COVID-19 pandemic [11, 12]. Moral distress was found to relate to the volume of care of patients infected with COVID-19, access to personal protective equipment, and communication from leaders [8]. Working in a stressful, less supportive environment during the COVID-19 pandemic has been associated with increased moral injury [12]. While a link has been proposed between moral distress/injury and adverse mental health outcomes [13, 14], there is little empirical data and a lack of knowledge about potential factors that may help prevent and better manage moral distress in HCWs. Moral resilience, which refers to the capacity to sustain or restore one's integrity in response to moral adversity [15], has been proposed as a pathway to mitigate the detrimental effects of moral adversity [16]. Sharing similar features with general resilience, moral resilience specifically incorporates individual factors that can help HCWs practice in a manner that reflects their intentions, character, and integrity [15, 17] while confronting an ethically adverse situation without lasting detrimental effects of moral distress.

…

Our findings provide empirical evidence about the importance of moral resilience for the mental health of HCWs facing a global crisis. In our Canadian sample, there were significantly more frequent PMDEs, higher levels of moral distress, more severe stress, anxiety and depression symptoms, and higher occurrences of diagnosed mental disorders in HCWs exposed to patients with COVID-19 compared to those who were not.

…

Moral resilience is premised on the belief that certain types of moral adversity in high stakes contexts (e.g. pandemic) are unavoidable and in some cases not modifiable. Enhancing moral resilience may help individuals restore their moral agency and autonomy when facing PMDEs. Self-regulatory skills such as mindfulness, tools to amplify certain dimensions of moral resilience like moral efficacy, and practices that foster self-stewardship and buoyancy can restore stability and agency so that HCWs can experience less distress [34, 35]. The goal of reducing distress is not aimed at creating complacency or tolerance of unethical practices or moral adversity, but rather to acknowledge and confront the sources of distress and to provide resources to reduce their short- and longterm detrimental effects [15]. Doing so may reduce the build-up of harmful moral residue and consequent risk of burnout, depression and secondary post-traumatic stress disorder (PTSD) [36]. We observed that higher moral resilience is associated with greater support from employers/co-workers. Hence, bolstering relational integrity, being true to one's values while respecting the values of others, and embracing shared moral endeavour and interconnection, may be ways to amplify individual moral resilience within the context of the clinical team or organization

[15]. Individual integrity is intertwined with the integrity of those served and of those with whom HCWs collaborate to deliver care including colleagues, organizations and the broader society. This finding is counter to claims that focusing on moral resilience puts undue burden on individuals and diverts attention from systemic contributions to the sources of moral adversity in healthcare [37].

…

In addition to enhancing support from the employer and colleagues, strengthening institutional structures to identify and respond to ethical concerns, creating spaces for dialogue and discussion [38], structured debriefings about troublesome cases, and peer to peer support led by facilitators trained in the nuances of ethical practice may amplify the foundation of moral resilience. Interventions such as the Mindful, Ethical Practice and Resilience Academy (MEPRA) [35] or other programs aimed at proactively developing and strengthening the capacities associated with moral resilience may provide a needed resource for HCWs.

19. **Virani, 2021 - Moral distress amid COVID-19: A frontline emergency nurse's perspective** (more descriptive than theoretical)

Moral distress is "the experience of knowing the right thing to do while being in a situation in which it is nearly impossible to do it."2 Compared with other healthcare professionals during a pandemic, ED nurses are more vulnerable to experiencing moral distress, which has intensified amid COVID-19.3-5 One out of 3 nurses experience moral distress, and 1 out of 10 leave the profession.7 Nurses and staff physicians are twice as likely to leave the profession due to moral distress compared with other healthcare professionals.6,7

Faced with issues such as insufficient time to manage competing demands, limited resources such as PPE, the responsibility to implement new no-visitor policies, changing clinical practices, among many others, nurses have been expected to make morally challenging decisions that may result in devaluing patients' wishes and reducing nurse-patient interaction.

20. **Willis, 2015 - Moral decisions, moral distress, and the psychological health of nurses**

A significant minority of participants became markedly distressed as they talked about previous experiences of distress within the interview. This chapter shall explore how past distress may remain a dormant part of a person's subjectivity and re-emerge or become (re)enacted in the narrations of those past distressing experiences (this can be related to what Hardingham [2004] called 'moral residue'). The term (re)enacting is used, firstly, in line with the position taken in Chapter 4 that all emotions and affects are enacted. That is, emotions are seen as affective practices (Wetherell, 2012; cf. Burkitt, 1997, 1999, 2014). Since the enacting of distress emerged in these interviews as individuals spoke of previous experiences of distress, it is suggested that it constitutes a re-enacting of that past distress. Participants were both enacting present distress and re-enacting past distress. Hence the term (re)enacting. Secondly, the present participle is preferred to the past tense because it implies an ongoing process of (re)enaction within the flow of both subjectivity and social relations.

21. **Moussa, 2022 - Why Do Street-Level Bureaucrats Risk Themselves for Others? The Case of Public Health Care Workers in Egypt (Masters thesis)**

Street-level bureaucracy (SLB) is a sociological concept that tries to clarify the operational practices and convictions of public service employees in their daily duties, and the manners by which they sanction public arrangement in their routine work. It analyzes the working environment with special attention to the government assistance and training. The concept depends on the idea that public administrations address 'the coal mineshafts of government 6 assistance where the "hard, filthy and risky work" of the state' is finished (Cooper et al., 2015). The belief that risk-taking might be deliberate and regular appears to be inadmissible to the psychometric methodology. Thus, in the sociological writing, the human being by nature is fearful in hazardous situations and attempts at avoiding situations that might be of danger to him/her (Lupton & Tulloch, 2002). Biological altruism is considered one of the fundamental theories regarding why individuals endanger their lives for other people. In developmental science, an organic entity is said to act philanthropically when its conduct benefits others at the expense of itself (Biological Altruism, 2013). Different speculations examine the starting points of goodness, focusing on the belief that people have the organic potential to think often about the wellbeing of others. The physical capability of SLBs to care for others is innate and evolves with experience and interactions with others (Cohen & Golan-Nadir, 2020). Furthermore, the desire to assist clients or serve society is not always sufficient. Helping often entails faith in the ability of a particular street-level bureaucrat to do so. Both organizational and environmental factors can influence one's willingness and ability to put one's life on the line for others. Indeed, according to Lipsky (1980), the distinguishing feature of street-level work is the interaction between employees and customers. 7 The conservation of resources theory, which states that people aim to build and defend what they value, could also explain such behavior of risking their lives for others. As a result, people want to maintain control over the resources required to achieve their goal of defending what they value. Individuals aim to create circumstances that will protect and promote their integrity (Cohen & Golan-nadir, 2020).

The findings of the present study suggest that patriotism, a sense of responsibility, religious beliefs, public recognition, and appreciation are the most significant factors influencing health care workers as SLBs to risk their lives for others during the COVID-19 pandemic. Moreover, the study results show that while good compensation packages, donations, and peer support boost the health care workers' motivation during their work in quarantine hospitals, the lack of organizational preparedness, and noncompliance with preventive measures by citizens are areas for improvements in order to obtain better outcomes. Overall, healthcare employees have stated that they are willing to put their lives on the line for others as part of their profession. The findings demonstrate that this willingness of SLBs cannot be attributed to one specific dominant factor but to a combination of three major elements of a sense of responsibility, religious beliefs, and public recognition.

22. **Shukla, 2019 - HIV Health Care Providers as Street-Level Bureaucrats: Unreflective Discourses and Implications for Women's Health and Well-Being**

Street-level bureaucrats Lipsky's (1980/2010) propositions about frontline workers and their role in how government policies are administered and implemented is reflected in the following interview extracts and analysis of health worker's narratives. HCPs interviewed for this study met the following assumptions in Lipsky's theory: (1) SLBs are well-intentioned and at least initially committed to public service. Over time the constraints of their work environments may erode their ideals and negatively impact service delivery; (2) SLBs operate within severely resource-constrained environments where they face a paucity of personal, organisational, and community resources; (3)

SLBs in their client-facing role end up interpreting policies and therefore shape what service delivery and public policy become on the ground. Because SLBs often need to justly apply meagre resources with policies that may be conflicting, their role makes discretion inevitable; and (4) As part of their service patterns, SLBs develop coping mechanisms to deal with the pressures and the resource constraints of their jobs (Lipsky 1980/2010). Some examples of SLB's coping mechanisms include 'creaming', generating time efficiencies, and limiting assessment of client needs or outcomes from service delivery. 'Creaming' involves SLBs identifying and prioritising clients who are likely to demonstrate the effectiveness of the government service, therefore, clients who are most likely to succeed are chosen first for services. SLBs also cope in their work environment by treating clients in ways that make optimal use of their time. SLBs may use cursory information to make judgment calls or may decide that some clients are worthier of receiving time-consuming services than others, or SLBs may transfer work responsibilities by referring clients to other organisations. The HCP participants in this study demonstrated these SLB characteristics as will be detailed in the Results. Lipsky's framework dovetails with Moxley's observations that the advocate's compromise entails work to use a relational approach to incrementally accentuate the humanity and strengths of the marginalised client and to advocate for 'more and better' (2014, 288); this also entails balancing the conflict of 'relentless action for just treatment' (289) with collaboration with clients and institutional representatives.

Meaning-making Meaning-making is the process by which individuals make sense of, or find connections among events, circumstances, and patterns of human behaviour (Merleau-Ponty 1962). Meaning-making assists people in making sense of their world and life. In the larger study, women's perceptions and experiences of caregiving in light of their own health challenges were examined and additional interviews with HCPs explored their experiences of providing care to these women caregivers living with HIV. Here we focus on examining the way HCPs as SLBs make meaning of women's caregiving roles and help constitute those meanings in dialogues with women. Because social meanings of phenomena are constructed in social spaces through interaction and the processes of meaning-making, dialogues between actors (HCPs and women clients) play an important role in constructing social meanings (Merleau-Ponty 1962; Berger and Luckmann 1966). Collectively developed and repeated dialogues assign the validity of truth to social events, circumstances, or phenomena under consideration in a given socio-historical context (Berger and Luckmann 1966; McNamee and Gergen 1999). There are no autonomous beings (Merleau-Ponty 1962): individuals are but a part of the meaning-making process in which no singular actor individually exercises influence on the outcomes of the meaning-making process. The construction of meaning is essentially co-created through acceptance, discussion, argument, questioning, inquiring, resistance, and other modes of dialogical interaction. In this study, the meanings made about informal care and the salience of women's health status are co-created through dialogues between HCPs and women caregivers living with HIV. Although our broad aim is to explore how HCPs perceive women's needs and how their advocacy affects services, we ETHICS AND SOCIAL WELFARE 135 also want to understand how these mutually constituted meanings affect HCPs' service provision and beliefs about their clients, as well as the broader societal implications for their clients. These interactions come with the appearance of psychological and other benefits for women which, we argue, come with unintended underlying harm. In empirically examining the perceptions and interventions of these (non-physician/-nurse) health care workers, we respond to Banks' call to reflect upon the ethical concerns and dilemmas that come as a result of such work (Banks 2008).

23. Virtanen, 2018 – Street-level bureaucrats as strategy shapers in social and health service delivery: Empirical evidence from six countries

The concept of the SLB, as developed by Lipsky (1980), needs however to be embedded into a more sophisticated discussion highlighting the tensions inherent within the role, and one which is dependent upon organisational context and personal interests. For instance, the assumption that SLBs develop explicit strategies to maintain themselves as resources and powerful actors in the system misses the relevant discussion on discretion, coping under the pressure of change, resisting change, adopting a paternalistic mentality towards service users and even engaging in policy sabotage. Tuurnas et al. (2015) have conceptually distinguished three separate, though overlapping, strategies that are important in the construction of service systems. In the following, we adopt this threedimensional strategy typology as the framework upon which to present our empirical findings. The first strategy conveys the idea that SLBs try to play an active role as policy makers. Given this, SLBs should be understood as a reform resource and as powerful actors who influence policy implementation. We argue that Lipsky offers an intellectual space from which to explore how SLBs demonstrate official (public) policy in the context of their relationships with their service users. Despite the fact that there are formal rules and vertical control over their work, SLBs apply the rules and create their own ethical codes in order to best tackle their work tasks. SLBs act in the boundary between the two worlds – the public service systems and the users of services – translating policy goals into concrete and perceived actions. This relation is reciprocal in essence, meaning that the SLBs also transfer the information of the effectiveness and productivity of the service interventions (from the boundary in-between the system and service users) vertically from 'street-level' to, in hierarchical terms, the upper levels of decision-making. As such, it is from here that the idea of SLBs as policy makers comes. The second strategy deployed by SLBs relates to working practices. This means that SLBs take an active role in defining the content of their day-to-day work. A significant amount of research evidence already exists on SLB actions in relation to working methods, values and practices, as well as on the discretion, freedom and self-interest of frontline workers in public organisations (e.g. Ellis et al., 1999; Evans and Harris, 2004). SLBs' ability to increase their level of discretion in respect of their working practices is a key tool in their day-to-day existence. Discretion here concerns the extent to which a worker has, in a specific context, the freedom to innovate and focuses on the factors that give rise to that freedom in that context. Looking more closely at the debate over this notion of discretion, it is clear that significant differences exist between what we may term the 'continuation' and 'curtailment' positions, differences which focus on beliefs about organisations' desire for and ability to secure control and on the workers' ability to resist control and seek discretion (Evans and Harris, 2004; Hupe and Buffat, 2014). SLBs' third strategy refers to professionalism. This relates to the fact that SLBs try to increase their professional groups' autonomy as a part of the service system. This perspective highlights the role of professionalism, and the notion that SLBs face constant difficulties in maintaining control in different organisational structures, and especially in large organisational settings. These include the need to consider signals from political superiors, organisational arrangements, enhancements in staff capacity and managerial supervision. Lipsky's original notion was designed to acknowledge and identify such a function for professionals as a way of understanding how they are active players themselves in the process of forming policy. The problem here is surely that in the 35years since Lipsky wrote his famous work the relationship between the state and the individual has fundamentally changed, as

has the role of the 'professional' as an intermediary between the two. In an era when people were recipients of welfare, welfare professionals were designated as being facilitators, making sure that both sides were serviced – there was significant room for discretion. In the new existence, however, where performance standards and indicators are centrally set and where service users have actionable rights and do not just receive state 'charity', the level of discretion open to SLBs is likely to be much smaller.

CMOC 5 – Health-seeking Behaviour Change

With the introduction/increase in OOPs, health-seeking behaviour change, compounded by health illiteracy, led to reduction in primary care usage, increased emergency care, medication mismanagement, delayed treatment

Search Summary – Title: health literacy AND health-seeking behaviour = 4

Search 2: Title: financial AND health-seeking behaviour = 10

(11 potentially relevant, 7 full text available)

24. **Akuffo-Henaku, 2019 - Financial Sources and Health Seeking Behaviour among the Elderly in Ghana** (Masters thesis)

The theoretical underpinning for this study is the Andersen and Newman Framework of Health Services Utilization. The purpose of this framework is to discover conditions that either facilitate or impede utilization (Aday & Andersen, 1974). The goal being, to develop a behavioural model that provides measures of access to medical care. The framework was first developed in the 1960s and has since gone through four phases. An individual's access to and use of health services is considered to be a function of three characteristics namely, predisposing factors, enabling factors and need factors (Ward, 1977). Predisposing factors cover the socio-cultural characteristics of individuals that exist prior to their illness, social structure (education, occupation, ethnicity, and culture), health beliefs (attitudes, values, and knowledge that people have concerning and towards the health care system) and demographics such as age and gender (Aday & Andersen, 1974; Anderson, 2005). The enabling factors comprise the logistical aspects of obtaining care. These include personal/family (the means and know-how to access health services, income, health insurance, a regular source of care, travel, extent and quality of social relationships), community (available health personnel and facilities, and waiting time) and possible additions (genetic factors and psychological characteristics) (Bass, & Noelker, 1987; Aday & Andersen, 1974). Finally, the need factors. These tend to be the most immediate cause of health service use, from functional and health problems that generate the need for health care services. According to Andersen (2005), perceived need will improve the understanding of care-seeking and observance of a medical regimen. This is because, it represents how people view their own general health and functional state, as well as how they experience symptoms of illness, pain, and worries about their health and whether or not they judge their problems to be of sufficient importance and magnitude to seek professional help (Andersen, 2005).

The Health Belief Model was also developed in the early 1950s by social scientists at the U.S. Public Health Service (Anderson, 2005). The primary goal was to understand the failure of people to adopt disease prevention strategies or screening tests for the early detection of disease. Later, the model was employed to understand patients' responses to symptoms and compliance with medical

treatments. The model proposes that a person's belief in a personal threat of a disease together with a confidence in the usefulness of the recommended health behavior or action will determine the prospect for the person to adopt the behavior. University of Ghana http://ugspace.ug.edu.gh 19 This model draws from psychological and behavioral theory with the basis that the two aspects of health-related behavior are the desire to avoid illness, or on the other hand, recover if already ill; and the belief that a specific health action will avert or remedy illness. In due course, an individual's course of action often depends on the person's opinions of the merits and obstacles related to health behavior. There are six constructs of the model (Anderson,1995) . The first four constructs were developed as the original tenets. The last two were added as research about the model evolved. These tenets include perceived susceptibility, perceived severity, perceived benefits, perceived barriers, cue to action and self-efficacy. This model is criticized as being limited in terms of not accounting for a person's attitudes, beliefs or other factors that dictate a person's acceptance of a health behaviour (Anderson, 2005). Another weakness of this model is its assumption that every individual has access to equal amounts of information on the disease (Porter, 2000). It also does not capture environmental or economic factors that may impede or facilitate the recommended intervention. Another behavioural theory is the theory of planned behaviour. This theory started as the theory of reasoned action in 1980 (LaMorte, 2019). It was meant to predict an individual's intention to engage in a behaviour at a specific time and place. The main intention was to explain all behaviours over which people have the ability to exert self-control. The key tenet in this model is behavioural intent (LaMorte, 2019). These intentions are influenced by the attitude about the likelihood that the behaviour will have the expected outcome and the subjective evaluation of the risks and benefits of that outcome. This theory states that behavioural achievement is subject to motivation/intention and ability/behavioural control. It distinguishes between three types of beliefs-behavioural, normative and control (LaMorte, 2019). The six main constructs underpinning this theory include, attitudes, behavioural intention, subjective norms, social norms, perceived power and perceived behavioural control. Some limitations of the theory cover the assumptions that behaviour is the end-product of a linear decision-making process, and does not consider that it can change over time; the individual has access to resources and opportunities to be successful in performing the desired behaviour, despite the intention. Additionally, it does not account for other variables that feed into behavioural intention and motivation such as fear, past experience or threat.

25. Mazlan, 2020 - E-HEALTH LITERACY AS A MEDIATOR ON RELATIONSHIP BETWEEN PREDICTORS AND ONLINE HEALTH INFORMATION-SEEKING BEHAVIOUR

1.7.2 Comprehensive Model of Information Seeking (CMIS)

The CMIS (Johnson, 1997) has been used extensively in the literature related to health information and its seeking (Case, Andrews, Johnson, & Allard, 2005; Rice, McCreadie, & Chang, 2001). In the seeking of information related especially to cancer, CMIS has seven factors grouped under three categories which are antecedents, information carrier factors, and information-seeking actions (Johnson et al., 2001; Johnson & Meischke, 1993). Antecedents are the main factors affecting individuals' health information seeking which include demographic characteristics, personal direct experience with health conditions, salience, and beliefs. The second category, information carrier factors, refers to individuals' perceptions of the features and conditions of information sources. These include the characteristics and the utility of the information sources selected. The third category in the CMIS is the information-seeking actions.

1.7.5 Salience (Unmet Health Information Needs)

Salience means personal significance and interests (Johnson & Meischke, 1993). According to the CMIS, individuals are usually motivated to search for information when they realize that they are missing some salient information (Case et al., 2005). Salience refers to the extent to which people are missing personally significant resources and events (Johnson, 1997). Johnson had suggested that uncertainties or limited knowledge can cause people to seek more information. Based on this idea, Johnson regarded insufficient information environments as a factor of salience. Salience is measured by the individuals' perceived needs and necessity for information and assistance of others (Johnson et al., 1995).

1.7.6 Beliefs (internet Self Efficacy)

Based on the CMIS, beliefs refer to an individuals' self-efficacy which is how much the individual perceive they have control over the future or that there are effective to prevent, treat and control a particular condition. This belief not only effect information carrier factors (e.g., trust in information sources), but also the individuals' exposure to information sources (Johnson, 2001). This definition supports the notion that if individuals have a low sense of self-efficacy or believe that knowing more about a cancer will not allow them to solve their problems, then they are not likely to seek health information. Basically, perceptions of powerlessness can lead to insufficient health-related knowledge (Caiata-Zufferey et

al., 2010; Leydon et al., 2000), because people have no motivation to learn more about their health care when they feel like they have no control (Caiata- Zufferey et al., 2010).

26. Muhanga, 2021 - Health Literacy, Health Behaviour and Healthcare Seeking Behaviour in Rural Settings in Tanzania: Towards a Conceptual Framework

Airhihenbuwa (2007:177) argues that ―improved knowledge and a change in attitude are the twin engines that generate expected outcomes in behaviour.‖ However, the study suggests that having knowledge without a change in attitudes cannot lead to behavioural change. Muhanga and Malungo (2017) assert that there is a strong connection between attitude and behavioural changes. Freer, (2015: 16) argues that any individual is ―a rational being that, when given the correct knowledge, will make the rational choice of positive behaviour change.‖ However, this is not often the case because there are innumerable aspects which can stop an individual from making a ―rational‖ choice. Other studies (Freudenberg, 2000; Freer 2015) have identified the systemic issues of poverty, structural constraints and culture among such precluding factors. Airhihenbuwa‘s PEN-3 model explains health beliefs and actions within the context of culture, history, and politics (Airhihenbuwa, 1995, 2007). The model postulates that health behaviour within an African context can be best understood within the cultural identity of Africans. The model further insists that even for other education interventions to be effective there is a need to attach them to African culture.

The study, out of which this conceptual framework was developed, theoretically was guided by PEN-3 model (Airhihenbuwa, 1995:29-34). Under cultural identity domain, the model recognizes the role of health education in empowering People, Extended Family and Neighbourhood (PEN) to make informed health decisions and perform roles that are appropriate in their families and communities. Relationships and Expectations is another domain of the model. Perceptions comprise of the knowledge, attitudes, values, and beliefs, within a cultural context, that may facilitate or hinder personal, family, and community motivation to change. The model assumes that there are enablers which are cultural, societal, systemic, or structural influences that may enhance barriers to change. The enablers are such as availability of resources, accessibility, referrals, employers, government officials, skills and type of services. Nurturers refer to the degree to which health beliefs, attitudes, and actions are influenced and mediated, or nurtured, by extended family, kin, friends, peer and the community. PEN-3 model also acknowledges Cultural Empowerment reflected in Positive Behaviour, Existential Behaviour and Negative Behaviours (PEN): Positive Behaviours are those that are based on beneficial actions and beliefs. Those ought to be encouraged. The success and sustainability of program relies on such behaviours. Existential Behaviour refers to practices, behaviours and/or cultural beliefs that are inherent to a group and have no harmful health consequences. One should not target to change such behaviours for change because they cannot lead to program failure. Negative behaviours are harmful beliefs and actions that affect health of a victim. Before attempting to change negative behaviours, health providers need to examine such behaviours within their historical, political, and cultural contexts (Airhihenbuwa, 1995). The theory goes beyond just valuing

the influence of health information hence health knowledge on health behavioural changes through health education by looking at cultural, historical, and political context (Freer, 2015). Although the model is widely used, there is no empirical information for its application in studies that are OHA based.

A conceptual model by Paasche-Orlow and Wolf (2007) indicates HL to be influenced by sociodemographic and economic characteristics. These characteristics in Fig.1 are referred to as subjects' characteristics (including the level of education attained, gender, age, and occupation). Several other studies (Rauschenbach and Hertel, 2011; Marinaccio et al., 2013; Muhanga, 2017a; Kaale and Muhanga, 2017; Muhanga and Malungo, 2017b) have reported on sociodemographic and economic related characteristics to have predictive effects on other variables. Subjects' characteristics were traced to analyse their effects on HL, HB and HCSB. Other models, Pathway Model and Determinant Model of Illness Behaviour also demonstrate that the decision to engage with particular medical channels is influenced by a variety of socio-economic variables (Akinnawo and Oguntimehin, 1997; Tipping and Segal, 1995).

Health of a population is not completely under the control of an individual citizen, nor of a doctor (except in some instances of individual disease), but is substantially under the control of the social relations of the society. Health is political much as power is exercised over it. There are ways public policy has been determining health in the society. Public policy as determinant of health, is acknowledged by Bambra et al., (2003) and Williamson and Carr (2009). At every local community, there are by laws, directives or even policies which form part of local politics. In order to analyse the influence of local politics on HL, HB and HCSB, a variable on Fig. 1 on political aspects was included. The intention here was to give answers to questions on how local leadership involved itself in promoting health related aspects for the local community. Together with this, local governance involvement of health related aspects was also analysed. This is in line with the fact that LGAs have been mandated by the Tanzania National Health Policy responsibilities with respect to health administration in their areas of jurisdiction (URT, 2007a; 2007b). Noteworthy, there are no any local government laws, directives or even policies that have been formulated on health care, diseases prevention and health promotion. There is no evidence on ways the bylaws, directives or even policies have managed to influence people's health. Knowledge is an important intervening variable in most of social and health related studies. This idea is accommodated in PEN-3 model (Airhihenbuwa, 1995, 2007). Theoretically, prior knowledge on environment management practices, health, and diseases, forms an important aspect of HL, HB and HCSB. Knowledge and awareness have a lot to influence on HL, HB and HCSB. The constructed conceptual framework (Fig.1) indicates prior knowledge having direct link with One Health Literacy. This is based on the fact that lack of knowledge in health related aspects has a number of outcomes. The outcomes include poor understanding of the complex nature of their health; medical care and conditions; poorer comprehension of medical information; low understanding and use of preventive services plus impacts of certain individuals behaviours on their health (see Davis et al., 1996; Baker et al., 1997; Baker, et al., 1998; Bennett et al., 1998; Doak et al.,1998). In this study, prior knowledge was assessed and its influence on HL, HB and HCSB was analyzed. It is apparent that HL is partly about people's access to health information and their capacity to use it effectively to make appropriate health decisions in order to promote and maintain good health (Ratzan and Parker, 2000; TARSC, 2009; Berkman et al, 2010). The communication, information, and technology that people intermingle with each day is the one that moulds ideas about health and behaviours of the people.

These processes make up the context and the ways professionals and the public search for, understand, and use health information, significantly impacting their health decisions and actions. Issues of access to information describe individuals' information seeking behaviour. In this context, it was important to address aspects of people's access to information in general and health information in particular much as at the core of health literacy is an aspect of access to health information. There is also an indication from previous study (Kambarage et al., 2003) that there are significant disease incidences which are undetected, unreported or underreported. Some studies (Karimuribo, 2007, et al) have attributed occurrences of these incidences of failing to detect and report to low awareness among other factors. Unquestionably, low awareness is partly connected to lack of information. Ultimately the intention of assessing access to information and types of information accessed was to give an answer to whether information accessed was adequate, understood and used in such ways which promoted and maintained good health by observing the kind of HB and HCSB exhibited. Therefore the influence of HISB on HL, HB and HCSB was analysed. Variables on access to information, types of information accessed and Information seeking behaviour were added to the conceptual framework in Fig. 1. Obviously, health care provider-patient interaction can play role in raising awareness on health and healthy choices, sharing health messages, removing barriers and creating supportive networks and environments. This can then influence HL, HB and HCSB. This supposition is supported by Making Every Contact Count Approach (NICE, 2007 as quoted by NWPH2 , n.d; Beck et al., 2002). Service characteristics which include aspects related to health care setting (i.e access to services in terms of physical proximity), perceived quality of service, the quality of medical care in terms of technical efficiency have been cited as key determinants of demand for health care (Mwabu et al., 1993; Ellis et al, 1994; Sahn et al., 2003; Baker 2006). Others being, psychological and monetary costs of taking action (including costs, time, money, effort, stigma, social distance, feeling of humiliation and the like); beliefs in the efficiency of recommended health care (itself related to beliefs about the cause of the disease). The conceptual framework takes into account efficiency in terms of accessing a particular modern human and animal health care service (availability, perceived costs involved and proximity –located within walking distance or not).

…

Theory of Reasoned Action is framed on thesis that behavioural intentions are influenced by attitudes and norms. A desired health outcome occurs if an individual adheres to a particular behaviour. The influence other people, such as a spouse, have on an individual's behaviour emanate from subjective norms (Nutbeam et al., 2010; Glanzet al., 2008). According to Nutbeam et al.,(2010), social norms theory suggests that much of people's behaviour is influenced by their perception of how other members of their social group behave. The traditions and customs have been considered to have a lot of influence on behavioural aspects. There are instances where certain practices have been maintained on a consideration. That is, they have customary values regardless of their consequences. In the context of an observation that the efforts of the government on improving health services and educating people to become more health literate did not yield positive outcomes. In search for the realities, it was important to investigate on perceptions and attitudes of the people towards HIBs. Health Belief Model (HBM) predicts that individuals will take action to protect their health, if they perceive themselves to be susceptible to a problem, and serious

consequences. In addition, individuals also need to believe a course of action is available that will reduce their susceptibility, and that the benefits of taking action outweigh the costs or barriers (Rosenstock, Strecher and Becker, 1994; Sheeran and Abraham, 1995 as cited by Hausmann-Muela et al., 2003). According to a model by Kuoeger, (1983) as cited in WHO (1995:5) the choice of healthcare is also influenced by beliefs in the efficiency of recommended health care (itself related to beliefs about the cause of the disease). The conceptual framework further captures local people‘s experiences: seeking health services, availability of treatment (traditional versus modern), knowledge and beliefs about diseases, and cosmopolitan outlook of diseases. Those have been covered partly under service characteristics; disorder characteristics and prior knowledge (see Fig.1). Andersen‘s behavioural model of health-care-use presupposes that the utilization of health care is influenced by the predisposition, the ability and the need to use health services (Andersen and Newman, 1973). Andersen grouped factors influencing the utilization into three main categories which are population characteristics, health care systems and the external environment (Andersen, 1995): This is presented in Fig. 1 partly under perceptions on diseases and other social aspects also under services characteristics.

…

The importance of lay networks and cultural factors (beliefs about disease causation and the potential efficacy of different forms of care have been said to affect the toleration or not of different symptoms) for determining health seeking behaviour (see for example WHO, 1995;Amaro and Gornemann, 1991; McCormack, 2012). The conceptual framework also brings on board social networking to analyse the influence of lay networks and some other cultural factors on the choice of a healer and maintenance of some HL, HB and HCSB. Social Cognitive Theory (Social Learning Theory) emphasizes the influence of other people on individual‘s behaviours, observational learning and the role of self-efficacy (Nutbeam et al., 2010). Behavioural economics and choice architecture approach recognizes that people do not always behave rationally, with behaviours often being governed by instinct, emotion, past events and the people around us (National Social Marketing Centre, 2011).

First redrafting CMOCs after parallel searches

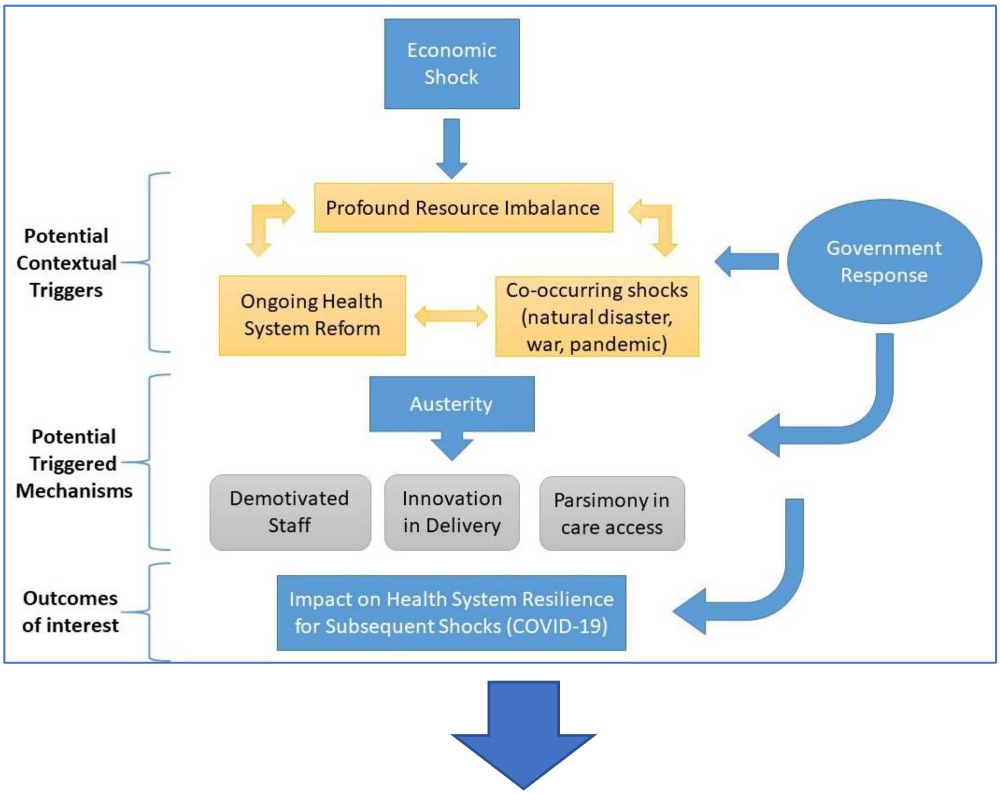

	CMOC from systematic search **(bold search terms)**	CMOC refinement after parallel searches
1	**Health systems** decision-making was highly influenced by outside agents (e.g. TROIKA) within the broader austerity agenda(1-5), but there was a lack of **transparency** about these outside influences from policy makers, compounded by poor communication from management and lack of co-production with frontline staff (6-8), led to a lack of ownership and buy-in from those delivering care and a distrust of the decision-making agenda(6, 9, 10)	**Health systems** decision-making was highly influenced by outside agents (e.g. TROIKA) with an emphasis on ***external transparency (11)*** and attendant emphasis on activity-driven metrics(2, 8, 10, 12-14) ***(selective transparency) (15)***, however there was a lack of ***internal* transparency (11)** about the policy decisions, compounded by poor communication and/or leading frontline workers to distrust the decision-making agenda ***and a resistance to change*** (16). **Discussion point:** *Legacy is that communication flow upstream may have been jeopardised leaving management in the dark.* Legacy of having the wrong kind of information to make informed policy decisions – metrics driven by selective transparency, rather than based on interpersonal and professional practices (what's captured within black boxes) (15).
2a	In context of restrictive **fiscal policies** (staffing, consumables, treatment options,	In context of compromised transparency around restrictive fiscal policies (staffing, consumables, treatment options, available

	available time with patient), a perceived **value** shift is evident for health professionals, from patient-focused to economic (10, 14, 17-19), with long-term consequences resulting in a diminished view of the profession, apathy and **burnt-out** (7, 10, 14, 20-24).	time with patient) and the attendant shift to financial and procedural principles, there was an ***erosion of ethical values (25) and moral disequilibrium for health professionals (26)***, resulting in **professional dissonance** (27), a diminished view of the profession, apathy and burnt-out (7, 10, 14, 20-24). **Discussion point:** Legacy around moral resilience, and health professional's capacity to sustain or maintain their integrity. Coping mechanisms include passion and job satisfaction (26), 'good enough practice' (27), having access to information, receiving support, having access to resources necessary to do the job, and having the opportunity to learn and grow (16).
3	*In order to achieve efficiencies* - information systems for example were introduced to track activity and spending (2, 8, 10, 12-14), **health professionals** perceive a loss of **autonomy** (3, 12, 14, 17, 19, 24) and decision-making **power** (3, 10), leading to a sense of **powerlessness** (13, 28) and detachment and ultimately a resistance to change and conflict between front line workers and policy decision makers / management (2, 7, 10).	With the shift to external/selective transparency and prioritisation of KPIs related to activity, finances, performance (11, 15)<br><br>health professionals perceive a loss of autonomy and decision-making power, feeling powerless and detached,<br><br>leading to a lack of organizational commitment and job satisfaction and increased professional dissonance and burnout (16)
4	**Street level bureaucrats:** A sense of professional / **moral** duty or **ethical** decision making, solidarity with patients or fellow health professionals (19, 21, 29, 30) led to health professionals circumventing policy to deliver care (legal, informal referrals, treat regardless of ability to pay) (3, 6, 21, 30-33), but ultimately lead to strain on frontline workers, increases ER use but more stable health outcomes than originally predicted (34).	When faced with circumstances that challenge their moral position and that of the medical profession, in the case of austerity - when vulnerable populations such as migrants in Spain were excluded from accessing free healthcare, health professionals sabotage policy to deliver care (35), in an effort to maintain control over what they value while balancing system needs with service-user needs (35, 36), having undergone reflective equilibrium (ethical decision making that tests system beliefs with other beliefs sets)(37), however sustained and unrelenting restrictive policies can ultimately lead to moral distress in the longer term when health professionals felt powerless to sustain resistance of growing restrictive policies (38-40) or when 'creaming' or generating efficiencies were

		no longer possible (41) (e.g. doing more with less and then less with less). **Discussion point:** *Legacy issue = moral residue where past moral distress can lie dormant until the next shock when it resurfaces (42).*
5	With the introduction/increase in **OOP**s, **health-seeking behaviour** change (33, 43), compounded by **health** il**literacy** (1, 18, 32, 43, 44), led to reduction in primary care usage (43), increased emergency care, medication mismanagement (1, 6, 32, 45), delayed treatment	

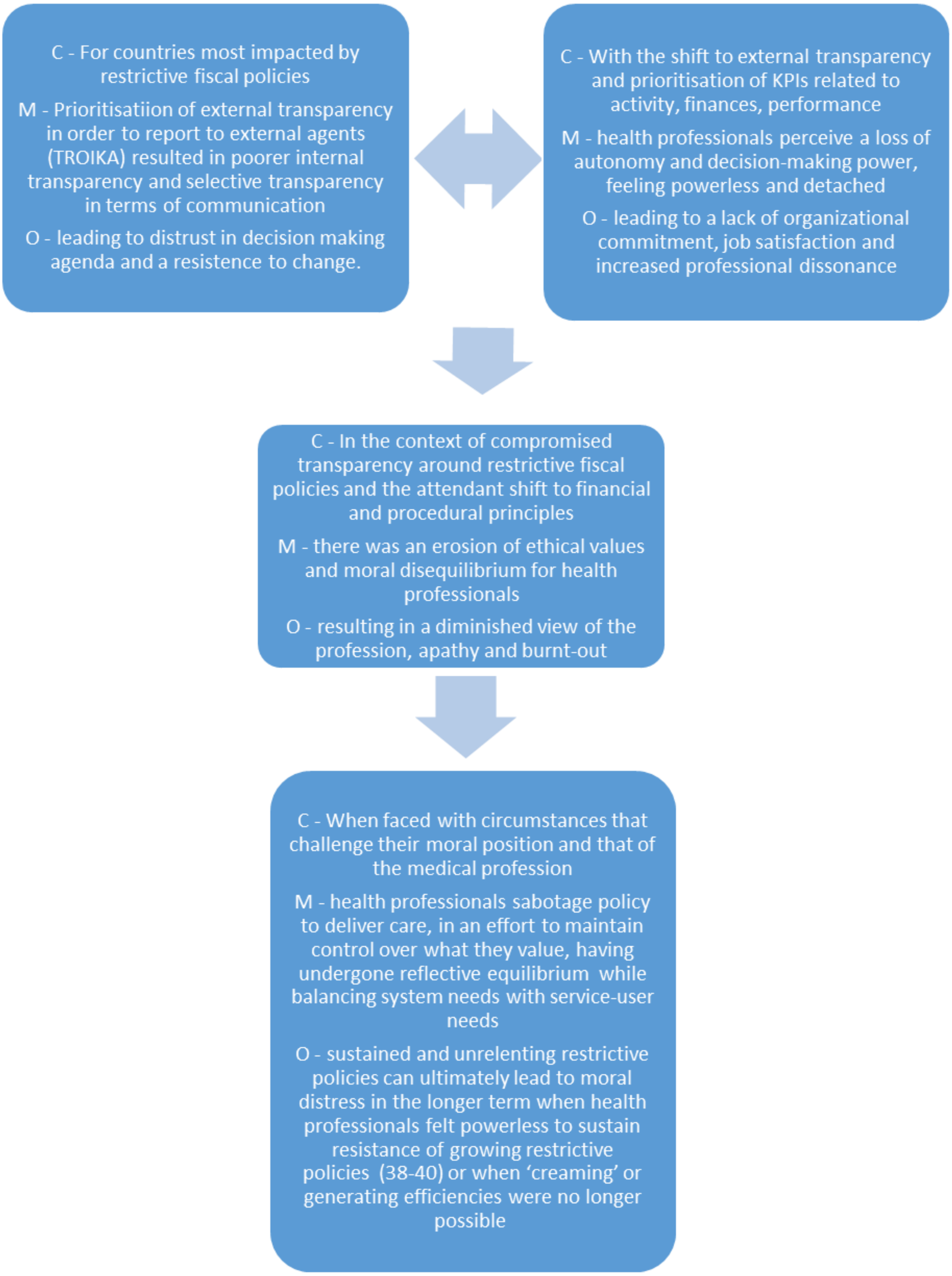

References

1. Carney MA. "Sharing One's Destiny": Effects of austerity on migrant health provisioning in the Mediterranean borderlands. Social Science and Medicine. 2017;187:251-8.
2. Kentikelenis A, Karanikolos M, Reeves A, McKee M, Stuckler D. Greece's health crisis: From austerity to denialism. The Lancet. 2014;383(9918):748-53.

3. Hollingworth W, Rooshenas L, Busby J, Hine CE, Badrinath P, Whiting PF, et al. Using clinical practice variations as a method for commissioners and clinicians to identify and prioritise opportunities for disinvestment in health care: a cross-sectional study, systematic reviews and qualitative study. NIHR Journals Library Health Services and Delivery Research. 2015;04:04.
4. Leider JP, Resnick B, Kass N. Tradeoffs in resource allocation at state health agencies. Journal of public health management and practice : JPHMP. 2014;20(6):566-79.
5. Thomas S, Keegan C, Barry S, Layte R, Jowett M, Normand C. A framework for assessing health system resilience in an economic crisis: Ireland as a test case. BMC health services research. 2013;13:450.
6. Cervero-Liceras F, McKee M, Legido-Quigley H. The effects of the financial crisis and austerity measures on the Spanish health care system: A qualitative analysis of health professionals' perceptions in the region of Valencia. Health Policy. 2015;119(1):100-6.
7. Fana T, Goudge J. Austerity, resilience and the management of actors in public hospitals: a qualitative study from South Africa. Bmj Global Health. 2021;6(2).
8. Gleeson LL, O'Brien GL, O'Mahony D, Byrne S. Thirst for change in a challenging environment: healthcare providers' perceptions of safety culture in a large Irish teaching hospital. Irish Journal of Medical Science. 2021.
9. Olafsdottir AE, Allotey P, Reidpath DD. A health system in economic crises: a case study from Iceland. Scandinavian journal of public health. 2013;41(2):198-205.
10. Pellinen J, Matto T, Sippola K, Rautiainen A. Blame game or dialogue? Financial, professional and democratic accountabilities in a complex health care setting. Accounting Auditing & Accountability Journal. 2018;31(2):626-50.
11. Balushi MA. How internal transparency impacts organizational resilience: emerald.com; 2020.
12. Correia T, Dussault G, Pontes C. The impact of the financial crisis on human resources for health policies in three southern-Europe countries. Health Policy. 2015;119(12):1600-5.
13. Kerasidou A, Cribb A. Austerity, Health and Ethics. Health care analysis : HCA : journal of health philosophy and policy. 2019;27(3):153-6.
14. Kerasidou A. Empathy and Efficiency in Healthcare at Times of Austerity. Health care analysis : HCA : journal of health philosophy and policy. 2019;27(3):171-84.
15. Griffiths D. Resilience and transparency in social systems. Kybernetes. 2019.
16. Laschinger H, Finegan J, Shamian J, Wilk P. Workplace empowerment as a predictor of nurse burnout in restructured healthcare settings. Longwoods Review. 2003.
17. Barradas CDS, Nunes JA. Facing austerity: the decline in health access and quality of care for patients with cancer in Portugal. Historia, ciencias, saude--Manguinhos. 2017;24(4):933-51.
18. Balfe M, Brugha R, Smith D, Sreenan S, Doyle F, Conroy R. Considering quality of care for young adults with diabetes in Ireland. BMC health services research. 2013;13:448.
19. Tucker R, Moffatt F, Timmons S. Austerity on the frontline- a preliminary study of physiotherapists working in the National Health Service in the UK. Physiotherapy theory and practice. 2020:1-13.
20. Humphries N, McDermott A, Conway E, Byrne J, Prihodova L, Costello R, et al. 'Everything was just getting worse and worse': deteriorating job quality as a driver of doctor emigration from Ireland. Human resources for health. 2019;17(1):1-11.
21. Kerasidou A, Kingori P, Legido-Quigley H. "you have to keep fighting": Maintaining healthcare services and professionalism on the frontline of austerity in Greece. International Journal for Equity in Health. 2016;15(1).
22. Kerasidou A, Kingori P. Austerity measures and the transforming role of A&E professionals in a weakening welfare system. PLoS ONE. 2019;14(2).
23. Ortega-Galan AM, Ortiz-Amo R, Andina-Diaz E, Ruiz-Fernandez MD. The Sustainability of Public Social Services: A Qualitative Study. Sustainability. 2020;12(9).

24. Russo G, Rego I, Perelman J, Barros PP. A tale of loss of privilege, resilience and change: the impact of the economic crisis on physicians and medical services in Portugal. Health Policy. 2016;120(9):1079-86.
25. Littlejohns P, Chalkidou K, Culyer A, ... National Institute for Health and Care Excellence, social values and healthcare priority setting. Journal of the Royal …. 2019.
26. Ong C. Moral distress, moral equilibrium and the moral equilibrium framework: health professionals' well-being in the face of daily challenges to moral values and integrity: minerva-access.unimelb.edu.au; 2020.
27. Taylor M, Bentley K. Professional dissonance: Colliding values and job tasks in mental health practice. Community mental health journal. 2005.
28. Papadakaki M, Lionis C, Saridaki A, Dowrick C, De Brún T, O'Reilly-De Brún M, et al. Exploring barriers to primary care for migrants in Greece in times of austerity: Perspectives of service providers. European Journal of General Practice. 2017;23(1):128-34.
29. Brall C, Schröder-Bäck P, Porz R, Tahzib F, Brand H. Ethics, health policy-making and the economic crisis: A qualitative interview study with European policy-makers. International Journal for Equity in Health. 2019;18(1).
30. Heras-Mosteiro J, Sanz-Barbero B, Otero-Garcia L. Health Care Austerity Measures in Times of Crisis: The Perspectives of Primary Health Care Physicians in Madrid, Spain. International journal of health services : planning, administration, evaluation. 2016;46(2):283-99.
31. Cimas M, Gullon P, Aguilera E, Meyer S, Freire JM, Perez-Gomez B. Healthcare coverage for undocumented migrants in Spain: Regional differences after Royal Decree Law 16/2012. Health Policy. 2016;120(4):384-95.
32. Gogishvili M, Flórez KR, Costa SA, Huang TTK. A qualitative study on mixed experiences of discrimination and healthcare access among HIV-positive immigrants in Spain. BMC public health. 2021;21(1):385.
33. Porthe V, Vargas I, Sanz-Barbero B, Plaza-Espuna I, Bosch L, Vazquez ML. Changes in access to health care for immigrants in Catalonia during the economic crisis: Opinions of health professionals and immigrant users. Health Policy. 2016;120(11):1293-303.
34. Petrou P. The Impact of Financial Crisis, Austerity Measures, and Bailout Agreement on Cypriots' Health: The Memorandum Is Dead, Long Live the Memorandum. Value in Health Regional Issues. 2019;19:99-103.
35. Virtanen P, Laitinen I, Stenvall J. Street-level bureaucrats as strategy shapers in social and health service delivery: Empirical evidence from six countries. International Social Work. 2018.
36. Moussa M. Why Do Street-Level Bureaucrats Risk Themselves for Others? The Case of Public Health Care Workers in Egypt: fount.aucegypt.edu; 2022.
37. Edwards I, Delany C, Townsend A, ... Moral agency as enacted justice: a clinical and ethical decision-making framework for responding to health inequities and social injustice. Physical …. 2011.
38. Khokhar M. Stress, Resilience and Moral Distress among Health Care Providers During COVID-19 Pandemic2021.
39. Spilg E, Rushton C, Phillips J, Kendzerska T, ... The new frontline: exploring the links between moral distress, moral resilience and mental health in healthcare workers during the COVID-19 pandemic: Springer; 2022.
40. Virani S. Moral distress amid COVID-19: A frontline emergency nurse's perspective. Nursing, 51 (12), 39-43 doi: 101097/01NURSE000080007235132c1. 2021;15(12):39-43.
41. Shukla S, McCoyd J. HIV Health Care Providers as Street-Level Bureaucrats: Unreflective Discourses and Implications for Women's Health and Well-Being. Ethics and Social Welfare. 2019.
42. Willis M. Moral decisions, moral distress, and the psychological health of nurses: researchgate.net; 2015.
43. Doetsch J, Pilot E, Santana P, Krafft T. Potential barriers in healthcare access of the elderly population influenced by the economic crisis and the troika agreement: A qualitative case study in Lisbon, Portugal. International Journal for Equity in Health. 2017;16(1).

44. Poduval S, Howard N, Jones L, Murwill P, McKee M, Legido-Quigley H. Experiences Among Undocumented Migrants Accessing Primary Care in the United Kingdom: A Qualitative Study. International Journal of Health Services. 2015;45(2):320-33.
45. Alvarez-Galvez J, Suarez-Lledo V, Martinez-Cousinou G, Muniategui-Azkona E, Gonzalez-Portillo A. The impact of financial crisis and austerity policies in Andalusia, Spain: Disentangling the mechanisms of social inequalities in health through the perceptions and experiences of experts and the general population. International Journal for Equity in Health. 2019;18(1).
